# Supplementary material for: Monomeric α‐synuclein activates the plasma membrane calcium pump
Source: EMBO J. 2023 Nov 2;42(23):e111122. doi: 10.15252/embj.2022111122 (PMC10690453; doi:10.15252/embj.2022111122)
Supplement: Supplementary file 1 — Appendix S1 [file EMBJ-42-e111122-s004.pdf]

Appendix

Table of Contents

Supplementary Figures 2

Appendix Figure S1. aSN increases calcium export from depolarized SH-SY5Y cells 2

Appendix Figure S2. Purification of recombinant PMCAs 3

Appendix Figure S3. Supplementary PMCA activity assays 4

Appendix Figure S4. Purity of alpha-synuclein preparations 5

Appendix Figure S5. Sequence alignment of the A-TM3 loop region and the TM3 domain of PMCA2w/aΔ298-372, PMCA2w/aΔ298-383, ACA8 and SERCA1a 5

Appendix Figure S6. Supplement to figure 1A – raw images of the western blots presented in Figure 1 6

Appendix Figure S7. Supplement to the figure 1B - pull-down of aSN with PMCA immobilized by Calmodulin-sepharose 7

Supplementary methods 8

A model for aSN-dependent calcium regulation 8

Appendix Table S1. Initial values and parameters for the calcium regulation model. ... 12

Appendix Figure S8. 14

References 14

Computer Code - Transcriptomics analysis 15

Supplementary Figures

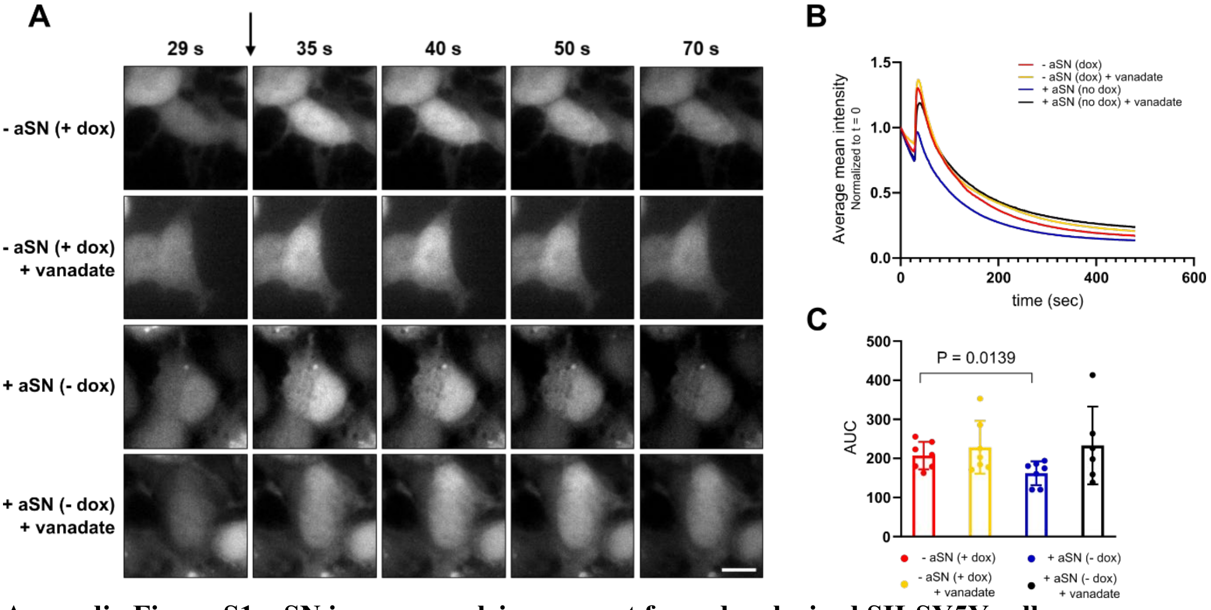

Appendix Figure S1. aSN increases calcium export from depolarized SH-SY5Y cells

Cytosolic calcium was monitored by Fluo-8 loading of SH-SY5Y cells with or without aSN. aSN expression was controlled by incubation with or without doxycycline (dox). Without dox present aSN is overexpressed and in the presence of dox aSN expression is repressed. Before recording, SERCA was inhibited by thapsigargin. Calcium influx was induced by addition of KCl. At the recording time of 30 sec. KCl was added to a final concentration of 90mM (indicated by arrows), depolarizing the SH-SY5Y cells. The calcium response was followed by time-lapse imaging.

A. Representative sequential images of Fluo-8 loaded cells at the time of depolarization. Arrow indicates time for addition of 90mM KCl and the time denoted is the experimental time. Scale bar: 10 µm.

B. Curve of average mean intensity from Fluo-8 loaded SH-SY5Y cells in: *blue* – SH-SY5Y cells without dox, expressing aSN, *black* - SH-SY5Y cells without dox, expressing aSN and treated with 1µM vanadate to inhibit ATPases, *red* - SH-SY5Y cells treated with dox, *yellow* - SH-SY5Y cells treated with dox and 1µM vanadate. The data is from 2 technical replicates with 2-4 cells per replicate, N (dox) = 8, N (dox + vanadate) = 7, N (- dox (aSN)) = 7, N (- dox (aSN) + vanadate) = 6.

C. Cytosolic Ca^{2+} level after the KCl-induced influx, quantified as the Area Under the Curve (AUC ± SEM). The response to KCl-induced influx was quantified as the Area Under Curve (AUC) from each measured SH-SY5Y cell. The data presented as mean ± SEM is from 2 technical replicates with 2-4 cells per replicate, N (dox) = 8, N (dox + vanadate) = 7, N (- dox (aSN)) = 7, N (- dox (aSN) + vanadate) = 6. The colors of the bars corresponds to the coloring of the curves in B. A two tailed students t-test was performed.

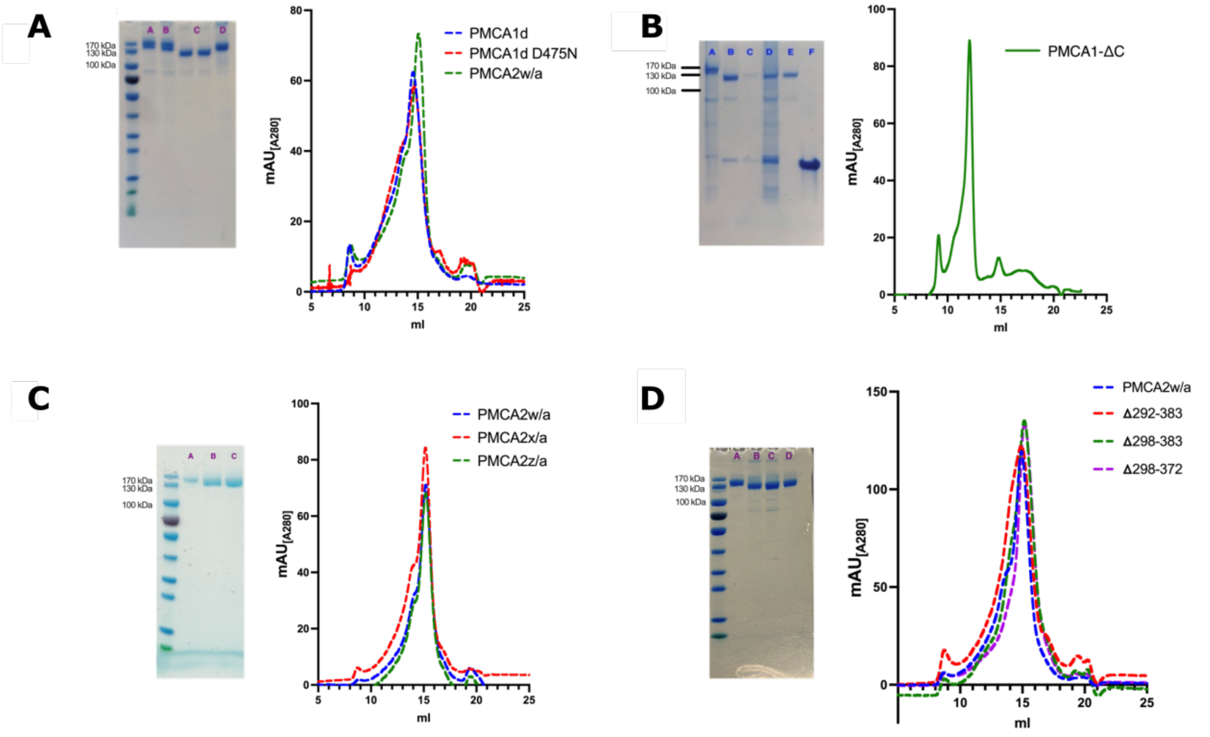

Appendix Figure S2. Purification of recombinant PMCAs

A - *Left* - SDS PAGE of purified PMCAs. (A) PMCA1d, PMCA1d D475N (inactive variant), PMCA1-ΔC, (D) PMCA2w/a. *Right* – Corresponding profiles of the size exclusion chromatography performed on the Superose 6 increase column.
B – *Left* – SDS-PAGE analysis of the purification of the PMCA1-ΔC. Full PMCA1d with internal TEV cleavage site at position 1074 was bound to CaM sepharose and washed (lane A – a sample of washed beads). After overnight incubation with His-tagged TEV flow-through 1 (FT1, lane B) was collected and beads were washed with buffer in a gravity-flow column (lane C - wash). FT1 was put through a Ni-Sepharose in a gravity-flow column to bind TEV and uncleaved PMCA1 (D – sample of washed sepharose beads). The flow-through from the nickel column was collected, concentrated to 500 µL, and subjected to size exclusion chromatography on Superdex 200 column. Lane E – peak fraction from the size-exclusion chromatography. Lane F – TEV protease. *Right* – Profile of the size exclusion chromatography of PMCA1-ΔC performed on the Superdex 200 increase column.
C – *Left* - SDS PAGE of purified PMCA2 splice variants (A-C) w/a, x/a, z/a. *Right* – Corresponding profiles of the size exclusion chromatography performed on the Superose 6 increase column.
D - *Left* - SDS PAGE of purified PMCA2w/a (A) and its deletion variants Δ292-383 (B), Δ298-383 (C), and Δ298-372 (D). *Right* – Corresponding profiles of the size exclusion chromatography performed on the Superose 6 increase column.

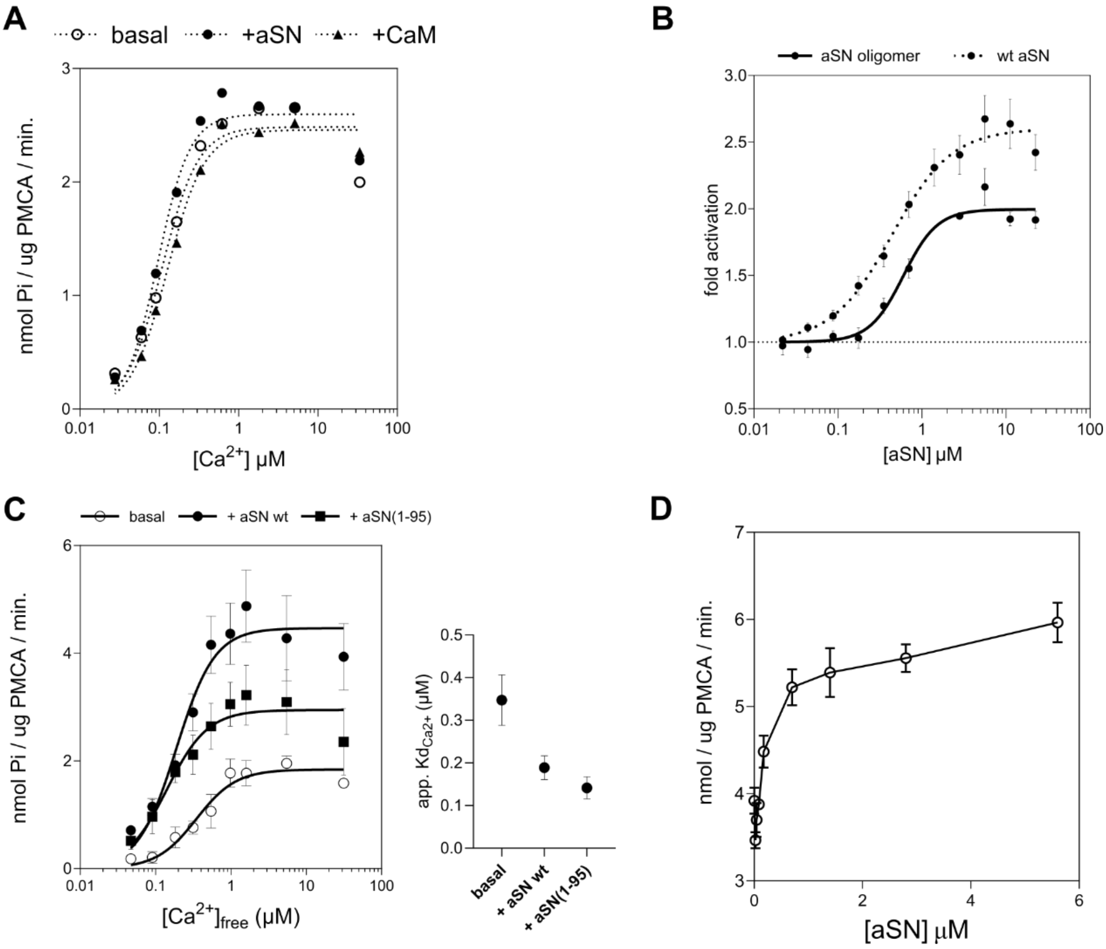

Appendix Figure S3. Supplementary PMCA activity assays

A. The activity of PMCA1-ΔC relipidated in brain PC lipids. Ca^{2+} titrations were performed in parallel in absence of the activating partner (basal activity), in the presence of 2.8 μM alpha-synuclein or in the presence of 1.2 μM CaM.

B. The effect of oligomeric aSN on the PMCA activity. PMCA1d fold activation by titrated full-length monomeric and oligomeric aSN. The assay was performed in presence of 1.8 μM free Ca^{2+} and brain lipid extract was used for the PMCA relipidation. Monomeric aSN data, presented here for comparison, is the same as in Figure 4. Oligomeric aSN experiment was performed in four independent replicates, where PMCA originated from single expression culture and aSN oligomers from two independent preparations.

All data shown as mean ± SEM.

C. The effect of full-length and C-terminally truncated $aSN_{(1\text{-}95)}$ on the calcium-dependent activity of PMCA1d. *On the left* – the calcium titration experiment was performed simultaneously in absence of activating partner (*empty circles*), in presence of the full-length monomeric a-SN (*filled circles*) and C-terminally truncated $aSN_{(1\text{-}95)}$ (*filled squares*). The pump was relipidated in brain lipid extract (BE). The lines are the best fit given by the Hill equation. *On the right* – apparent Kd_{Ca2+} values calculated from the fitted Hill plots with the apparent Kd values (μM) for Ca^{2+} as follows: PMCA1d basal (without aSN) – 0.347 ± 0.059, with aSNwt - 0.189 ± 0.028, with $aSN_{(1\text{-}95)}$ – 0.142 ± 0.026. The measurements were performed in three independent replicates, where the proteins originated from single expression culture.

D. The activity of C-terminally truncated PMCA4x (lacking the autoinhibitory domain) was measured as a function of monomeric a-SN concentration in presence of 1.8 μM free Ca^{2+}. PMCA was relipidated with brain extract (BE). The measurement was performed in independent duplicates, where proteins originated from single expression culture

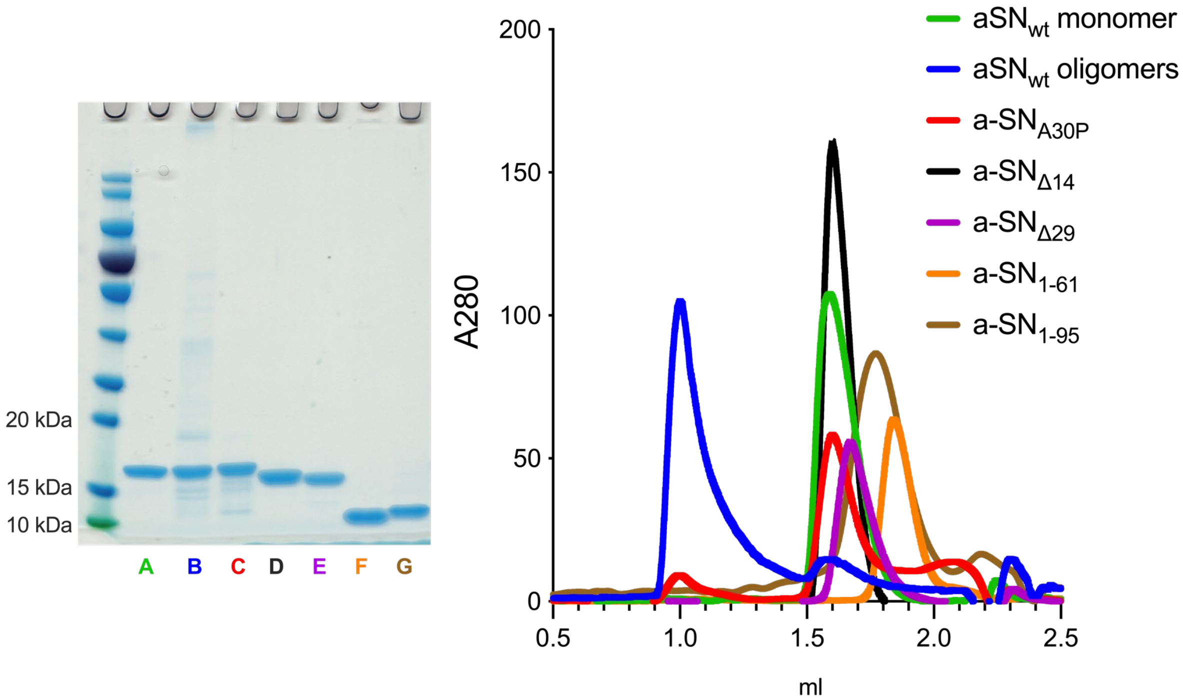

Appendix Figure S4. Purity of alpha-synuclein preparations.

On the left – SDS-PAGE analysis A – monomer; B – oligomers; C – A30P; D – Δ14; E – Δ29; F – (1-61); G – (1-95); *On the right* – size exclusion chromatography analysis performed on Superdex 200 increase 3.2/300 column. Monomeric forms were eluted at volume > 1.5 ml

A-TM3 linker region — TM3

```
PMCA2Δ298-372   273 - VTAVGVNSQTGIIFTLLGAGGEEEERKKASMHKKEKSVLQGKLTKLAVQIGKAGLVMSAI-TV
PMCA2Δ298-383   273 - VTAVGVNSQTGIIFTLLGAGGEEEE-----------SVLQGKLTKLAVQIGKAGLVMSAI-TV
ACA8            339 - VTGVGVNTEWGLLMASISEDNGEE------------TPLQVRLNGVATFIGSIGLAVAAAVLV
SERCA1a         223 - VATTGVSTEIGKIRDQMAAT------------EQDKTPLQQKLDEFGEQLSKVISLICVAVWL
```

Appendix Figure S5. Sequence alignment of the A-TM3 loop region and the TM3 domain of PMCA2w/aΔ298-372, PMCA2w/aΔ298-383, ACA8 and SERCA1a.

Positively charged residues marked blue, negatively – red. Italics show the acidic lipid binding site of PMCA2. Alignment of full sequences was performed with uniport.org, sequences from which presented fragments were taken have the following entry numbers in Uniprot: Q01814-2 (PMCA2w/a, modified for the alignment, to show both PMCA deletion variants), Q9LF79 (ACA8), and P04191 (SERCA1a).

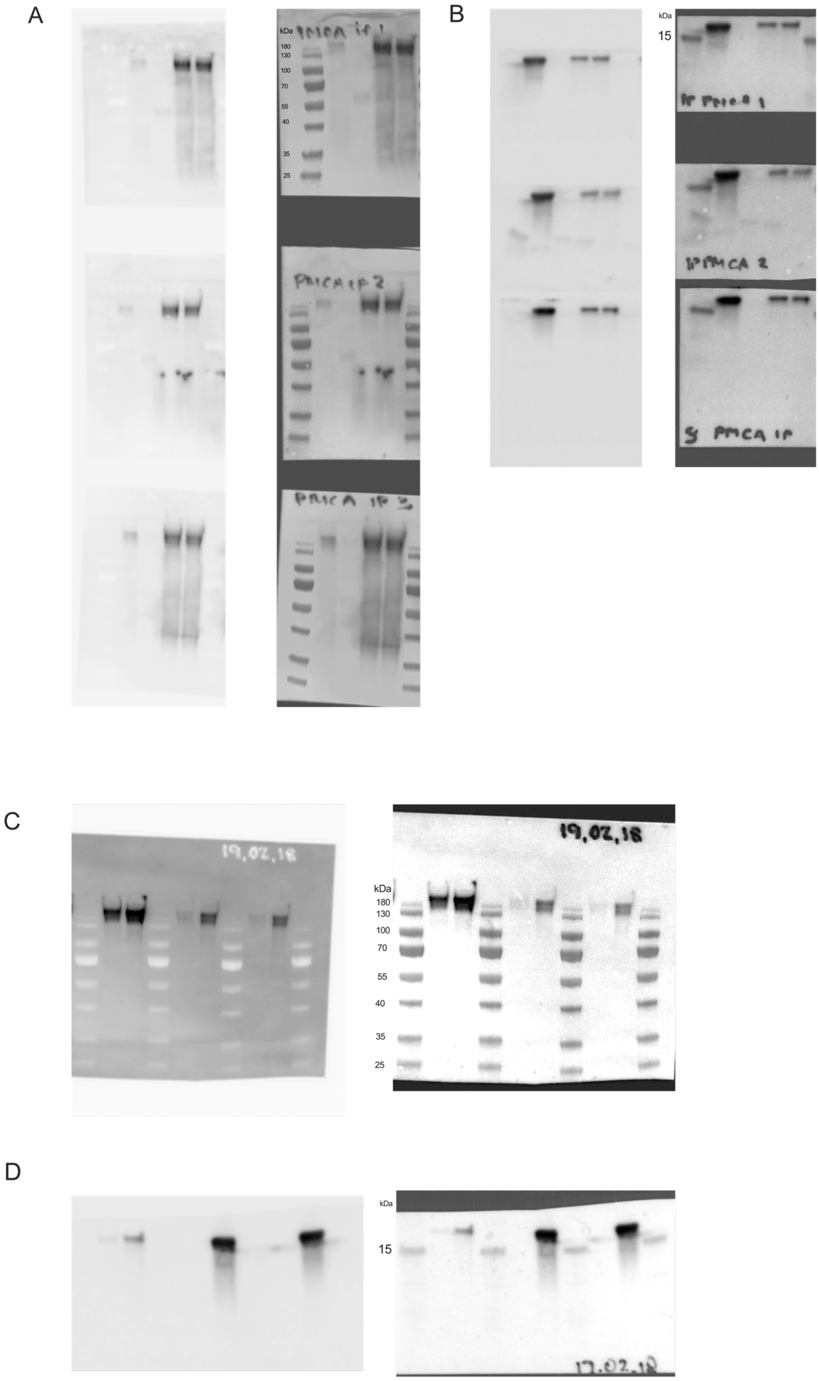

Appendix Figure S6. Raw images of the western blots presented on the Figure 1.

In all sections raw blots are presented on the left and blots merged with image displaying the molecular weight markers are on the right.

A and B. Co-IP assay, where total brain homogenates from aSN-knockout mice were incubated with exogeneous aSN and aSN-binding sepharose (ASY-1). Western blotting performed with anti-pan-PMCA (A) and anti-aSN antibodies (B).

The order of loading of samples is as follows from left: MW marker, input, CO-IP PBS, CO-IP mono, Co-IP oligo, marker

C and D. Co-IP assay, where detergent extracts of C57BL/6 mice were incubated with aSN-binding sepharose (ASY-1) to pull down endogenous aSN. Western blotting performed with anti-pan-PMCA (A) and anti-aSN antibodies (B). The order of samples is as follows: marker, input for IP with nonimmune IgG, input for IP with ASY-1, marker, IP with nonimmune IgG, IP with ASY-1, marker, IP with nonimmune IgG, IP with ASY-1, Marker.

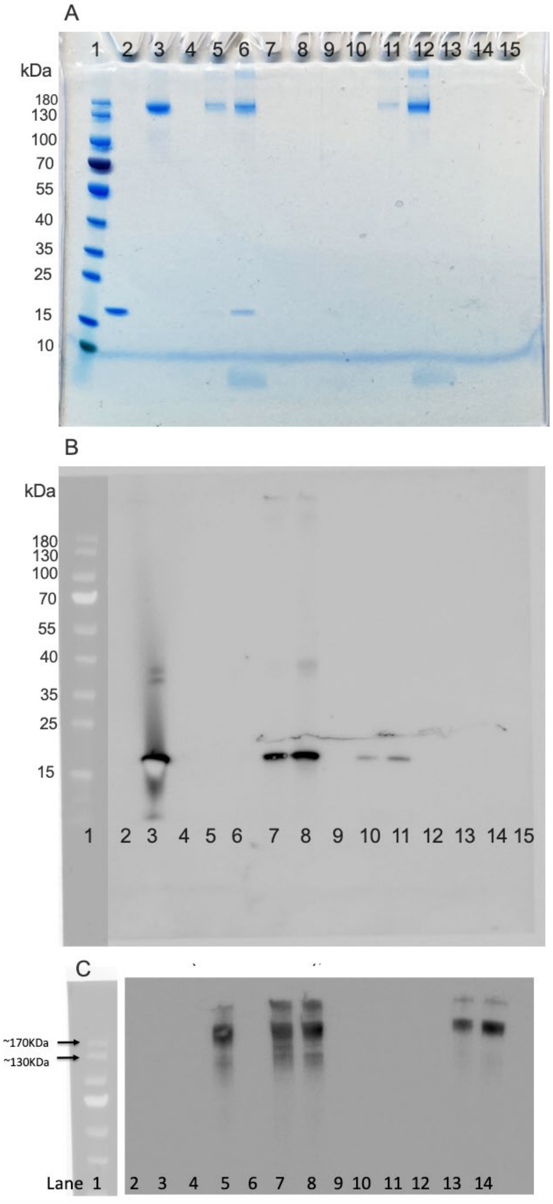

Appendix Figure S7. Pull-down of aSN with PMCA immobilized by Calmodulin-sepharose.

In this experiment pure protein preparations were used.
A. SDS-PAGE analysis, Coomassie staining
Lanes: 1 – MW marker, 2 – pure aSN, 3 – pure PMCA2w/a, 4 – empty lane, Following lanes contain samples incubated with CaM sepharose and eluted with EGTA-buffer: 5 – PMCA+aSN; 6 – PMCA2w/a+aSN+brain lipid extract (BE), 8 – aSN, 9 – aSN+BE, 11 – PMCA2w/a, 12 – PMCAw/a+BE; Lanes 7, 10, 13-15 do not contain any sample
B. Western blotting detection of aSN using anti-ASY-R1 rabbit primary antibodies and anti-rabbit HRP (Dako - P0217) as secondary antibodies
Lanes: Lanes: 1 – MW marker, 2 – pure aSN, 3-4 – empty laners, 5 – pure PMCA2w/a, 4-6 – empty lanes; Following lanes contain samples incubated with CaM sepharose and eluted with EGTA-buffer: 7 – PMCA+aSN; 8 – PMCA2w/a+aSN+brain lipid extract (BE), 10 – aSN, 11 – aSN+BE, 13 – PMCA2w/a, 14 – PMCAw/a+BE; Lanes 9, 13 and 15 do not contain any sample
C. Western blotting detection of PMCA using anti-PMCA (5F10, Abcam, ab2825) mouse primary antibodies and anti-mouse HRP secondary antibodies (Dako – P0260)
This figure shows the exact same membrane as shown on figure B, which was stripped and reprobed to detect PMCA.

Supplementary methods

A model for aSN-dependent calcium regulation

In order to conceptualize the findings of aSN-mediated PMCA activation and its influence on the Ca^{2+} concentration in the presynaptic terminal, we adapted an existing ODE-model published by Erler et al. (Erler, Meyer-Hermann et al., 2004), hereafter named the Erler-model, to take into account the new dependence of PMCA on aSN.

Here we briefly introduce the Erler model, before introducing the changes applied to take into account the aSN-dependence of PMCA.

The Erler-model describes Ca^{2+} - fluxes through PMCA, the H^+/Ca^{2+} exchanger NCX and the voltage gated Ca^{2+} channel VGCC and studies calcium dynamics during action potentials in absence and presence of external Ca^{2+} buffers like Ca^{2+} - fluorophores (Erler et al., 2004). Erler et al. made use of the Hodgkin-Huxley model (Hodgkin & Huxley, 1952, Keener & Sneyd, 2009) to describe the membrane potential dynamics during an action potential. The dynamics of the cytosolic Ca^{2+} ion concentration was described by the following differential equation:

$$\frac{d[Ca^{2+}]}{dt} = \frac{G}{z_{Ca}F}(J_{VGCC} - J_{PMCA} - J_{NCX} + J_{leak})\frac{1}{1 + T_{en} + T_{ex}}$$

Where J_{VGCC} denotes the influx of Ca^{2+} through voltage gated Ca^{2+} channels, J_{PMCA} and J_{NCX} denote Ca^{2+} effluxes mediated by PMCA and NCX respectively and J_{leak} denotes a leakage flux of Ca^{2+} ions across the membrane down its electrochemical gradient. The changes in Ca^{2+} concentration are given in $\frac{\mu M}{ms}$.
The term $\frac{G}{z_{Ca}F}$ is a conversion factor from current per unit of time to concentration per unit of time, which includes the geometry factor G in μm^{-1} representing the surface to volume ratio, the ion valence z_{Ca}, and Faraday's constant F in $\frac{As}{mol}$. As the term $\frac{1}{1+T_{en}+T_{ex}}$ is unitless, all fluxes J are defined in units of $\frac{aA}{\mu m^2}$. The term $\frac{1}{1+T_{en}+T_{ex}}$ represents calcium ion buffering through endogenous and exogenous buffers, where $T_{en}(c) = \frac{b_{en}^0 K_{en}}{(K_{en}+[Ca^{2+}])^2}$ and $T_{ex}(c) = \frac{b_{ex}^0 K_{ex}}{(K_{ex}+[Ca^{2+}])^2}$ with $K_{en} = \frac{k_{en}^-}{k_{en}^+}$ as the ratio of the rate constants, while b_{en}^0 and b_{ex}^0 describe the concentration of the total concentration of buffer proteins.

The calcium ion influx through the voltage-gated calcium channels (VGCC) is given by:

$$J_{VGCC} = \rho_V g_V(V) g_{Ca}(V_{ca} - V)$$

Where ρ_V denotes the surface density of VGCCs, g_V denotes the voltage dependent opening probability, g_{Ca} is the open pore conductivity, V is the membrane potential, and V_{Ca} is the reversal potential, described by the Nernst equation:

$$V_{Ca} = \frac{RT}{z_{Ca}F} ln\left(\frac{[Ca^{2+}]_{ext}}{[Ca^{2+}]}\right) - \Delta V_{eff}$$

Where R is the molar gas constant, T is the temperature, $[Ca^{2+}]_{ext}$ is the external calcium ion concentration and ΔV_{eff} is a correction factor for the linear approximation used for the single channel open current $g_{Ca}(V_{ca} - V)$ (Erler et al., 2004). The time dependence of voltage dependent opening probability is modeled by a single exponential approximation:

$$\frac{d}{dt} g_V = (g_\infty(V) - g_V))\frac{1}{\tau}$$

Where the opening probability reaches its asymptotic value $g_\infty(V)$ with time constant τ. The asymptotic value $g_\infty(V)$ can be described by a sigmoidal function:

$$g_\infty(V) = \frac{1}{exp\left((V_h - V)\frac{1}{\kappa}\right) + 1}$$

Where V_h is the half activation voltage and κ is the steepness at the inflection point of $g_\infty(V)$. The calcium ion efflux through PMCA and NCX are modeled as Hill equations:

$$J_{PMCA,Erler} = \rho_p I_p \frac{c^{n_p}}{c^{n_p} + H_p{}^{n_p}} \quad , \quad J_{NCX} = +\rho_x I_x \frac{c^{n_x}}{c^{n_x} + H_x{}^{n_x}} .$$

Where ρ_p and ρ_x are the specific surface densities, I_p and I_x are the universal maximum activity rates, H_p and H_x are the half activation concentrations, and n_p and n_x are the Hills coefficients of PMCA and NCX respectively. L is the leakage surface current density, which is determined by the steady state conditions, and ensures that the model can maintain equilibrium when unperturbed.

To account for the aSN concentration dependency of the PMCA activity, the flux through PMCA used by Erler et al. was replaced by another kinetic rate law, which can be derived from the following binding scheme, which allows for description of non-essential activation as seen by aSN. :

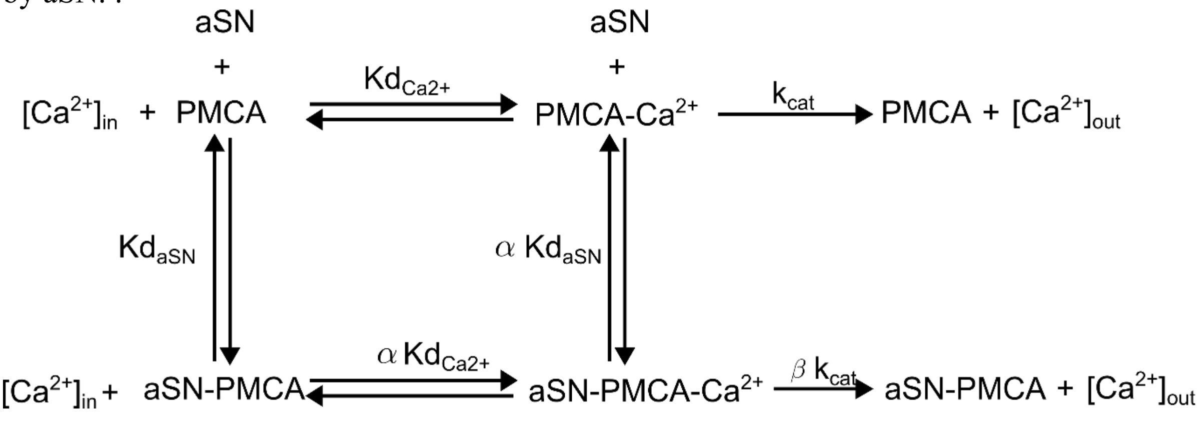

.

Where $K_{d,Ca}$ is the dissociation constant of calcium, $K_{d,aSN}$ is the dissociation constant of aSN, k_{cat} is the catalytic constant of PMCA, α is the reciprocal allosteric coupling constant, and β is the factor by which aSN affects the catalytic constant.

The resulting equation is (Baici, 2015):

$$J_{PMCA}\left(v_{max,PMCA}, \alpha, \beta, K_{d,Ca}, K_{d,aSN}\right) = \frac{v_{max,PMCA}\frac{[Ca^{2+}]}{K_{d,Ca}} + \beta\frac{v_{max,PMCA}\,[Ca^{2+}][aSN]}{\alpha K_{d,Ca}K_{d,aSN}}}{1 + \frac{[Ca^{2+}]}{K_{d,Ca}} + \frac{[aSN]}{K_{d,aSN}} + \frac{[Ca^{2+}][aSN]}{\alpha K_{d,Ca}K_{d,aSN}}} \quad \textbf{(S1)}$$

$$= v_{max,PMCA}\,\frac{\frac{[Ca^{2+}]}{K_{d,Ca}} + \beta\frac{[Ca^{2+}][aSN]}{\alpha K_{d,Ca}K_{d,aSN}}}{1 + \frac{[Ca^{2+}]}{K_{d,Ca}} + \frac{[aSN]}{K_{d,aSN}} + \frac{[Ca^{2+}][aSN]}{\alpha K_{d,Ca}K_{d,aSN}}}$$

In order to determine the kinetic parameters, the kinetic for PMCA was fitted to the PMCA1 activity in dependence of [Ca^{2+}] and [aSN] (**Figure 2A** left and **Figure 2B**), using the minimize function from scipy.optimize (VirtanenGommers et al., 2020) and the cost function:

$$\chi^2(v_{max}, \alpha, \beta, K_{d,Ca}, K_{d,aSN}) = \sum_i \left(\frac{y_i - J_{PMCA}(v_{max}, \alpha, \beta, K_{d,Ca}, K_{d,aSN})}{\sigma_i}\right)^2$$

With σ_i being the standard error of the mean corresponding to the data point y_i.

The optimal parameter set was given by:

$$v_{max,PMCA(fit)} = 1.93\,\frac{nmol\ Pi}{\mu g\ PMCA\ min}$$
$$\alpha = 0.29$$
$$\beta = 4.31$$
$$K_{d,aSN} = 5.5\ \mu M$$
$$K_{d,Ca} = 0.29\ \mu M$$

The resulting fit is shown in **Appendix Figure S8**. Note that the data points for high calcium concentrations were excluded from the fitting process.

Calculation of dependent parameters

The parameter $v_{max,PMCA(fit)}$ obtained by the fitting process is given in $\frac{nmol\ Pi}{\mu g\ PMCA\ min}$ and can not be translated directly into the units required by the model, i.e. $\frac{aA}{\mu m^2}$.

We therefore assumed that the flux mediated by PMCA in our updated version should equal the flux through the kinetic used by Erler et al. at the steady state Ca^{2+} concentration of 0.1 µM and an aSN concentration of 40 µM as measured by Wilhelm et al. (Wilhelm, Mandad et al., 2014), assuming that in the data used by Erler et al. also around 40 µM aSN were present. Thus, at $[Ca^{2+}]_{eq} = 0.1\ \mu M$ and $[aSN]_{eq} = 40\ \mu M$ the PMCA mediated fluxes should be equal, i.e.:

$$\rho_p I_p \frac{{[Ca^{2+}]_{eq}}^{n_p}}{{[Ca^{2+}]_{eq}}^{n_p} + {H_p}^{n_p}} \equiv J([Ca^{2+}]_{eq}, [aSN]_{eq}) =$$

$$v_{max,PMCA} \frac{\frac{[Ca^{2+}]_{eq}}{K_{d,Ca}} + \beta \frac{[Ca^{2+}]_{eq}[aSN]_{eq}}{\alpha K_{d,Ca} K_{d,aSN}}}{1 + \frac{[Ca^{2+}]_{eq}}{K_{d,Ca}} + \frac{[aSN]_{eq}}{K_{d,aSN}} + \frac{[Ca^{2+}]_{eq}[aSN]_{eq}}{\alpha K_{d,Ca} K_{d,aSN}}}$$

And hence:

$$v_{max,PMCA} = \rho_p I_p \frac{{[Ca^{2+}]_{eq}}^{n_p}}{{[Ca^{2+}]_{eq}}^{n_p} + {H_p}^{n_p}} \cdot \frac{1 + \frac{[Ca^{2+}]_{eq}}{K_{d,Ca}} + \frac{[aSN]_{eq}}{K_{d,aSN}} + \frac{[Ca^{2+}]_{eq}[aSN]_{eq}}{\alpha K_{d,Ca} K_{d,aSN}}}{\frac{[Ca^{2+}]_{eq}}{K_{d,Ca}} + \beta \frac{[Ca^{2+}]_{eq}[aSN]_{eq}}{\alpha K_{d,Ca} K_{d,aSN}}} .$$

The leakage flux $J_{leak,eq}$ is modeled as GHK-flux equation (Keener & Sneyd, 2009), which depends on the permeability of the membrane for Ca^{2+} (P_{Ca}), which was calculated using the steady state assumption:

$$J_{PMCA,eq} + J_{NCX,eq} + J_{VGCC,eq} + J_{leak,eq} \equiv 0$$

With $J_{PMCA,eq}$, $J_{NCX,eq}$ and $J_{VGCC,eq}$ being the fluxes mediated by PMCA, NCX and VGCC at equilibrium and

$$J_{leak,eq} = P_{Ca} \frac{z_{Ca}^2 V_{eq} F^2}{RT} \cdot \frac{[Ca^{2+}]_{eq} - [Ca^{2+}]_{ext} \cdot exp(-\frac{z_{Ca}^2 V_{eq} F}{RT})}{1 - exp(-\frac{z_{Ca}^2 V_{eq} F}{RT})},$$

It follows:

$$P_{Ca} = (J_{PMCA,eq} + J_{NCX,eq} + J_{VGCC,eq}) \cdot \frac{RT}{z_{Ca}^2 V_{eq} F^2} \cdot \frac{1 - exp(-\frac{z_{Ca}^2 V_{eq} F}{RT})}{[Ca^{2+}]_{eq} - [Ca^{2+}]_{exz} \cdot exp(-\frac{z_{Ca}^2 V_{eq} F}{RT})}$$

And finally:

$$J_{leak} = P_{Ca} \frac{z_{Ca}^2 V F^2}{RT} \cdot \frac{[Ca^{2+}] - [Ca^{2+}]_{ext} \cdot exp(-\frac{z_{Ca}^2 V F}{RT})}{1 - exp(-\frac{z_{Ca}^2 V F}{RT})} .$$

Complete ODE System

Finally, the entire system of ODEs is given by:

$$\frac{dm}{dt} = \alpha_m (1 - m) - \beta_m m$$

$$\frac{dn}{dt} = \alpha_n (1 - n) - \beta_n n$$

$$\frac{dh}{dt} = \alpha_h (1 - h) - \beta_h h$$

$$\frac{dV}{dt} = (-g_k\, n^4\,(V - V_k) - g_{Na}\, m^3\, h\,(V - V_{Na}) - g_{leak}\,(V - V_{leak}) + I_{app}) \,/\, C_m$$

$$\frac{dg_V}{dt} = (g_\infty(V) - g_V))\frac{1}{\tau}$$

$$\frac{d[Ca^{2+}]}{dt} = \frac{G}{z_{Ca}F}(J_{VGCC} - J_{PMCA} - J_{NCX} + J_{leak})\frac{1}{1+T_{en}+T_{ex}},$$

with the first four equation describing the Hodgkin-Huxley model as described by Keener and Sneyd (Keener & Sneyd, 2009), the fifth describing the change of the open probability of the voltage-gated Ca^{2+} - channels, the sixth describing the change in the cytosolic Ca^{2+} concentration.
The initial values and parameters are shown in **Appendix Table S1**.

Appendix Table S1. Initial values and parameters for the calcium regulation model.

Parameter	Value	Unit	Reference
m_0	0.05296		(Keener & Sneyd, 2009)
n_0	0.317732		(Keener & Sneyd, 2009)
h_0	0.595995		(Keener & Sneyd, 2009)
V_0	-65	mV	(Keener & Sneyd, 2009)
$g_{v,0}$	$6.236 \cdot 10^{-5}$		steady state assumption
$[Ca^{2+}]_0$	0.1	µM	(Erler et al., 2004)
G	6	µm	(Erler et al., 2004)
z_{Ca}	2		
F	96485	$\frac{As}{mol}$	
R	8.314	$\frac{J}{mol \cdot K}$	
T	310	K	
$[Ca^{2+}]_{ext}$	1500	µM	(Erler et al., 2004)
I_p	$2.7 * 10^{-21}$	C/ms	(Erler et al., 2004)
n_p	2		(Erler et al., 2004)
H_p	0.09	µM	(Erler et al., 2004)

ρ_p	9200	μm^{-2}	(Erler et al., 2004)
I_x	$4.8 * 10^{-19}$	C/ms	(Erler et al., 2004)
n_x	1		(Erler et al., 2004)
H_x	1.8	µM	(Erler et al., 2004)
ρ_x	$0.033 \cdot \rho_p$	μm^{-2}	(Erler et al., 2004)
g_{ca}	$14 \cdot 10^3$	fS	(Erler et al., 2004)
τ	1	ms	(Erler et al., 2004)
κ	6.3	mV	(Erler et al., 2004)
ΔV_{eff}	47	mV	(Erler et al., 2004)
V_h	-4	mV	(Erler et al., 2004)
ρ_v	3.1	μm^{-2}	(Erler et al., 2004)
K_{en}	0.5	µM	(Erler et al., 2004)
b_{en}^0	120	µM	(Erler et al., 2004)
K_{ex}	6	µM	(Erler et al., 2004)
b_{ex}^0	0	µM	(Erler et al., 2004)
α	0.29		**Appendix Figure S8**
β	4.31		**Appendix Figure S8**
$K_{d,aSN}$	5.5	µM	**Appendix Figure S8**
$K_{d,Ca}$	0.29	µM	**Appendix Figure S8**

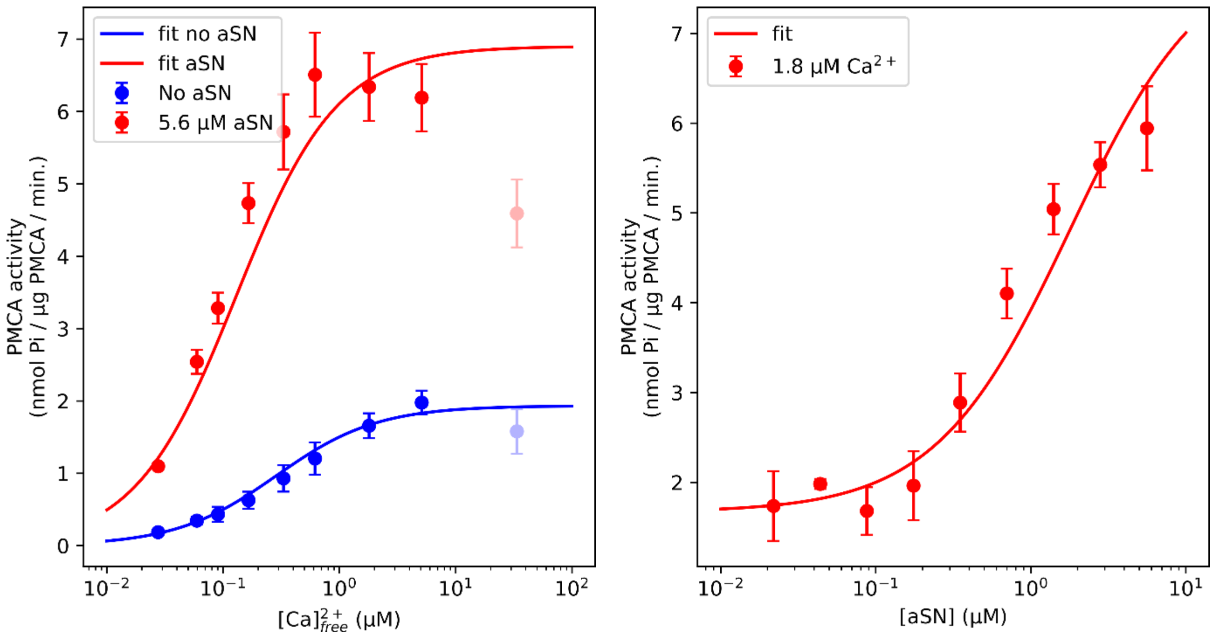

Appendix Figure S8. Monomeric alpha-synuclein acts as a non-essential activator of PMCA. The kinetic equation derived from the assumption of non-essential activation of PMCA by aSN was fitted to the Ca^{2+} and aSN dependence reported in **Figure 2**. The non-saturated data points (faded colors) were excluded from the fitting process.

References

Baici A (2015) Kinetics of Enzyme-Modifier Interactions: Selected Topics in the Theory and Diagnosis of Inhibition and Activation Mechanisms. *Vienna: Springer*

Erler F, Meyer-Hermann M, Soff G (2004) A quantitative model for presynaptic free Ca2+ dynamics during different stimulation protocols. *Neurocomputing* 61: 169-191

Hodgkin AL, Huxley AF (1952) A quantitative description of membrane current and its application to conduction and excitation in nerve. 117: 500-544

Keener J, Sneyd J (2009) Mathematical Physiology I: Cellular Physiology. *Springer, New York*

Virtanen P, Gommers R, Oliphant TE, Haberland M, Reddy T, Cournapeau D, Burovski E, Peterson P, Weckesser W, Bright J, van der Walt SJ, Brett M, Wilson J, Millman KJ, Mayorov N, Nelson ARJ, Jones E, Kern R, Larson E, Carey CJ et al. (2020) SciPy 1.0: fundamental algorithms for scientific computing in Python. *Nature Methods* 17: 261-272

Wilhelm BG, Mandad S, Truckenbrodt S, Kröhnert K, Schäfer C, Rammner B, Koo SJ, Claßen GA, Krauss M, Haucke V, Urlaub H, Rizzoli SO (2014) Composition of isolated synaptic boutons reveals the amounts of vesicle trafficking proteins. 344: 1023-1028

Computer Code – Transcriptomics analysis

The original code written in R for the purpose of the analysis of transcriptomics data.

The code relevant for the analysis displayed on the Figure 3A:

```
install.packages("tidyverse")
install.packages("BSDA")
library("tidyverse")

library("BSDA")

# Data import
PMCA3 <- read_tsv("PMCA3.csv")

PMCA1 <- read_tsv("PMCA1.csv")

PMCA4 <- read_tsv("PMCA4.csv")

asn <- read_tsv("alphasynuclein.csv")

# function grouping data by subgroup and calculating mean CRPKM within the
subgroup

CRPKMs_mean <- function(data) {
  data %>%
  group_by(Subgroup) %>%
  summarise(
    Order = mean(Order),
    CRPKMs = mean(CRPKMs),
    Group = Group)
}

# Applying the CRPKMs_mean function to the PMCA and aSN data

PMCA1_CRPKMs_mean <- CRPKMs_mean(PMCA1)

PMCA3_CRPKMs_mean <- CRPKMs_mean(PMCA3)

PMCA4_CRPKMs_mean <- CRPKMs_mean(PMCA4)

asn_CRPKMs_mean <- CRPKMs_mean(asn)

# Data import
exon_PMCA1 <- read_tsv("exon_incorporation_PMCA1.csv")

exon_PMCA3 <- read_tsv("exon_incorporation_PMCA3.csv")

exon_PMCA4 <- read_tsv("exon_incorporation_PMCA4.csv")

# function grouping data by subgroup and calculating mean exon incorporati
on value within the subgroup

exon_mean <- function(data) {
  data %>%
  group_by(EventID, Subgroup) %>%
  summarise(
```

```
    Order = mean(Order),
    Value = mean(Value, na.rm = TRUE),
    Group = Group)
}

PMCA1_exon_mean <- exon_mean(exon_PMCA1)

PMCA3_exon_mean <- exon_mean(exon_PMCA3)

PMCA4_exon_mean <- exon_mean(exon_PMCA4)

#filtering exon incorporation data for the relevant tissues - tissues with
actual PMCA expression

exsp_exon <- function(data1, data2) {
  exsp_tissue <- filter(data1, CRPKMs > 1.5)
    Tissue <- c(exsp_tissue$Subgroup)
    subset(data2, Subgroup %in% Tissue)
}

PMCA1_exsp_exon <- exsp_exon(PMCA1_CRPKMs_mean, PMCA1_exon_mean)
PMCA3_exsp_exon <- exsp_exon(PMCA3_CRPKMs_mean, PMCA3_exon_mean)
PMCA4_exsp_exon <- exsp_exon(PMCA4_CRPKMs_mean, PMCA4_exon_mean)


# filtering for relevant event IDs - events leading to the a variant at sp
lice site C variants:

#PMCA1c is achieved with HsaALTD0000577-1/2  (green)
#PMCA1a is achieved with HsaALTD0000577-2/2 (blue)
#PMCA1b is achieved without exon HsaEX0006882
#PMCA1a is achieved with exon HsaEX0006882 (yellow)
PMCA1_exsp_exon <- filter(PMCA1_exsp_exon, EventID == "HsaEX0006882")



#HsaEX0006895 with incorporation PMCA3x without PMCA3z, HsaEX0006897 with
then PMCA3a and without then PMCA3b
PMCA3_exsp_exon <- filter(PMCA3_exsp_exon,  EventID == "HsaEX0006897")

#PMCA4z is achieved without the HsaEX0006899 exon #PMCA4x is achieved with
the HsaEX0006899 exon #PMCA4a is achieved with the HsaEX0006904 exon #PMCA
4b is achieved without the HsaEX0006904 exon
PMCA4_exsp_exon <- filter(PMCA4_exsp_exon, EventID == "HsaEX0006904")

# function for calculation of mean incorporation of a specific splicing ev
ent for tissues within a designated interval of aSN expression

Event_mean_int_PMCA <- function(PMCA_data){

interval <- seq(0, 190, by=10)
exsp_asn <- list()
Event_mean_PMCA <- list()
Event_mean_all_tissues <- list()
Event_mean_brain <- list()
```

```
Event_mean_PMCA_tissue <- list()

i <- 1
while (i < length(interval)) {


  exsp_asn[[paste(interval[[i+1]])]] <- filter(asn_CRPKMs_mean, CRPKMs >=
interval[[i]] & CRPKMs < interval[[i+1]])
  Event_mean_PMCA[[paste(interval[[i+1]])]] <- subset(PMCA_data, Subgroup
%in% exsp_asn[[i]]$Subgroup)

  Event_mean_all_tissues[[i]] <- Event_mean_PMCA[[paste(interval[[i+1]])]]
%>%
    summarise(n_group = n())

  Event_mean_brain[[i]] <- Event_mean_PMCA[[paste(interval[[i+1]])]] %>%
  filter(Group == "Neural_crest" | Group == "Neural" | Group == "NPC" | Gr
oup == "Glia" | Group == "EmbrBrain") %>%
    summarise(n_group = n())

Event_mean_PMCA_tissue[[paste(interval[[i+1]])]] <- Event_mean_PMCA[[paste
(interval[[i+1]])]] %>%
  filter(Value >= 0) %>%
  group_by(EventID) %>%
  summarise(mean_PSI = mean(Value),
            sd_PSI = sd(Value),
            n_tissues = n()) %>%
  add_column(percent_brain = sum(Event_mean_brain[[i]]$n_group)/sum(Event_
mean_all_tissues[[i]]$n_group)*100) %>%
  add_column(asn_exp_interval = interval[[i+1]])


  i <- i + 1

}

Event_mean_PMCA_tissue <- data.table::rbindlist(Event_mean_PMCA_tissue)

}

Event_mean_int_PMCA1 <- Event_mean_int_PMCA(PMCA1_exsp_exon)

Event_mean_int_PMCA3 <- Event_mean_int_PMCA(PMCA3_exsp_exon)

Event_mean_int_PMCA4 <- Event_mean_int_PMCA(PMCA4_exsp_exon)


#Intervals for plotting mean incorporation of exons as a function of the a
SN expression interval

expression_interval <- c("0-10", "10-20", "20-30", "30-40", "40-50", "50-6
0", "60-70", "70-80", "80-90", "90-100", "100-110", "110-120", "120-130",
"130-140", "140-150", "150-160", "160-170", "170-180", "180-190")
```

```
PMCA1_a <- Event_mean_int_PMCA1
PMCA3_a <- Event_mean_int_PMCA3
PMCA4_a <- Event_mean_int_PMCA4

# The tissues within aSN intervals 110-120 and 130-140. These are outliers

asn_120 <- filter(asn_CRPKMs_mean, CRPKMs >= 110 & CRPKMs < 120)

asn_140 <- filter(asn_CRPKMs_mean, CRPKMs >= 130 & CRPKMs < 140)

labels <- tibble(x = c(95, 165),
                 y = c(5,0),
                 label = c(asn_120$Subgroup[1], asn_140$Subgroup[1]))

# Plotting mean incorporation of events leading to variant a at different
aSN intervals

ggplot() +
  geom_point(data = PMCA1_a, mapping = aes(x = asn_exp_interval, y = mean_
PSI, color = EventID), size = 2.5) +
  geom_point(data = PMCA4_a, mapping = aes(x = asn_exp_interval, y = mean_
PSI, color = EventID), size = 2.5) +
  geom_point(data = PMCA3_a, mapping = aes(x = asn_exp_interval, y = mean_
PSI, color = EventID), size = 2.5) +
  theme_bw() +
  theme(legend.title=element_blank()) +
  scale_color_manual(labels = c("PMCA1 \n HsaEX0006882", "PMCA3 \n HsaEX00
06897", "PMCA4 \n HsaEX0006904"), values=c("#FFCC33","#9900CC", "#0000FF")
) +
  labs(x = "aSN expression interval (CRPKMs)", y = "mean PSI \n PMCA exons
") +
  theme(axis.text.x = element_text(angle = 90, hjust = 1, vjust = 0.5)) +
  theme(legend.position="bottom") +
  scale_x_continuous(breaks = seq(10, 190, by = 10), labels = expression_i
nterval) +
  geom_label(data = labels, mapping = aes(x=x, y=y, label=label), size = 3
)+
  ggsave(width = 5, height = 4, filename = "mean_PSI_interval_PMCA_a_versi
on.png")

# Plotting with standard deviations

  ggplot() +
  geom_point(data = PMCA1_a, mapping = aes(x = asn_exp_interval, y = mean_
PSI, color = EventID), size = 2.5) +
  geom_point(data = PMCA4_a, mapping = aes(x = asn_exp_interval, y = mean_
PSI, color = EventID), size = 2.5) +
  geom_point(data = PMCA3_a, mapping = aes(x = asn_exp_interval, y = mean_
PSI, color = EventID), size = 2.5) +
  geom_errorbar(data = PMCA1_a, aes(x=asn_exp_interval, ymin=mean_PSI-sd_P
SI, ymax=mean_PSI+sd_PSI, width = 5))+
   geom_errorbar(data = PMCA3_a, aes(x=asn_exp_interval, ymin=mean_PSI-sd_
PSI, ymax=mean_PSI+sd_PSI, width = 5))+
   geom_errorbar(data = PMCA4_a, aes(x=asn_exp_interval, ymin=mean_PSI-sd_
```

```
PSI, ymax=mean_PSI+sd_PSI, width = 5))+
  theme_bw() +
  theme(axis.text.x = element_text(angle = 90, hjust = 1, vjust = 0.5)) +
  theme(legend.title=element_blank()) +
  scale_color_manual(labels = c("PMCA1 \n HsaEX0006882", "PMCA3 \n HsaEX00
06897", "PMCA4 \n HsaEX0006904"), values=c("#FFCC33","#9900CC", "#0000FF")
) +
  labs(x = "aSN expression interval (CRPKMs)", y = "mean PSI \n PMCA exons
") +
  theme(axis.text.x = element_text(angle = 90, hjust = 1, vjust = 0.5)) +
  theme(legend.position="bottom") +
    scale_x_continuous(breaks = seq(10, 190, by = 10), labels = expression
_interval) +
  geom_label(data = labels, mapping = aes(x=x, y=y, label=label), size = 3
)+
    ggsave(width = 5, height = 4, filename = "mean_PSI_interval_PMCA_a_ver
sion_error.png")

#t-test - calculate if there is a stastically significant difference betwe
en the mean PSI for tissues with aSN expression below 50 CRPKMs and above
50 CRPKMs.

interval_means <- function(PMCA_data, interval1, interval2) {
exsp_asn <- filter(asn_CRPKMs_mean, CRPKMs >= interval1 & CRPKMs < interva
l2)
  Event_mean_PMCA <- subset(PMCA_data, Subgroup %in% exsp_asn$Subgroup) %>
%
    group_by(EventID) %>%
    summarise(mean_PSI = mean(Value),
              sd_PSI = sd(Value),
              n_tissues = n())
}

#- For PMCA4
low_data_means <- interval_means(PMCA4_exsp_exon, 0, 50)

high_data_means <- interval_means(PMCA4_exsp_exon, 50, 190)


tsum.test(mean.x = (low_data_means[[1,2]]), s.x = (low_data_means[[1,3]]),
n.x = (low_data_means[[1,4]]), mean.y = (high_data_means[[1,2]]), s.y = (h
igh_data_means[[1,3]]), n.y = (high_data_means[[1,4]]))

##
##  Welch Modified Two-Sample t-Test
##
## data:  Summarized x and y
## t = -2.6275, df = 10.859, p-value = 0.02374
## alternative hypothesis: true difference in means is not equal to 0
## 95 percent confidence interval:
##  -53.460998  -4.680534
## sample estimates:
## mean of x mean of y
##  16.24105  45.31182
```

```
#for PMCA1
low_data_means_1 <- interval_means(PMCA1_exsp_exon, 0, 50)

high_data_means_1 <- interval_means(PMCA1_exsp_exon, 50, 190)

tsum.test(mean.x = (low_data_means_1[[1,2]]), s.x = (low_data_means_1[[1,3
]]), n.x = (low_data_means_1[[1,4]]), mean.y = (high_data_means_1[[1,2]]),
s.y = (high_data_means_1[[1,3]]), n.y = (high_data_means_1[[1,4]]))

##
##  Welch Modified Two-Sample t-Test
##
## data:  Summarized x and y
## t = -3.3017, df = 10.4, p-value = 0.007589
## alternative hypothesis: true difference in means is not equal to 0
## 95 percent confidence interval:
##  -73.90402 -14.53230
## sample estimates:
## mean of x mean of y
##  7.147292 51.365455

## Adjusting tables for a more convenient look for publication

PMCA1a <- PMCA1_a %>% mutate(across(is.numeric, ~ round(., 2)))

PMCA3a <- PMCA3_a %>% mutate(across(is.numeric, ~ round(., 2)))
PMCA4a <- PMCA4_a %>% mutate(across(is.numeric, ~ round(., 2)))

aSN_exp_int <- c("0-10", "10-20", "20-30", "30-40", "40-50", "50-60", "80-
90", "110-120", "120-130", "130-140", "180-190")

PMCA1a_adjusted  <- PMCA1a  %>% add_column(aSN_exp_int)
PMCA1a_adjusted[,c(7,2,3,4,5)]

PMCA4a_adjusted  <- PMCA4a  %>% add_column(aSN_exp_int)
PMCA4a_adjusted[,c(7,2,3,4,5)]

aSN_exp_int <- c("0-10", "10-20", "40-50", "50-60", "80-90", "120-130", "1
80-190")

PMCA3a_adjusted  <- PMCA3a  %>% add_column(aSN_exp_int)
PMCA3a_adjusted[,c(7,2,3,4,5)]
```

The code relevant for the analysis displayed on the Figure 3B:

```
library(tidyverse)

library(BSDA)

PMCA2 <- read_tsv("PMCA2.csv")

PMCA2_CRPKMs_mean <- PMCA2 %>%
  group_by(Subgroup) %>%
  summarise(
    Order = mean(Order),
    CRPKMs = mean(CRPKMs))

asn <- read_tsv("alphasynuclein.csv")

asn_CRPKMs_mean <- asn %>%
  group_by(Subgroup) %>%
  summarise(
    Order = mean(Order),
    CRPKMs = mean(CRPKMs))

exon_PMCA2 <- read_tsv("exon_incorporation_PMCA2.csv")

exon_PMCA2

#filtering for the relevant tissues - tissues with actual PMCA2 expression
exsp_PMCA2 <- filter(PMCA2_CRPKMs_mean, CRPKMs > 1.5)
exsp_PMCA2

#taking the expression levels into account:
PMCA2_exon_tissues <- c(exsp_PMCA2$Subgroup)
PMCA2_exon_tissues

PMCA2_exon_mean <- exon_PMCA2 %>%
  group_by(EventID, Subgroup) %>%
  summarise(
    Order = mean(Order),
    Value = mean(Value, na.rm = TRUE),
    Group = Group)

PMCA2_exon_mean

PMCA2_exon_mean <- filter(PMCA2_exon_mean, EventID == "HsaEX0006891" |  Ev
entID == "HsaEX0006892" |  EventID == "HsaEX0006893" )




ggplot(data = PMCA2_exon_mean) +
  geom_point(mapping = aes(reorder(Subgroup, Order), y = Value, color = PM
CA2_exon_mean$EventID)) +
  theme_bw() +
  theme(axis.text.x = element_text(angle = 90, hjust = 1, vjust = 0.5)) +
```

```
  theme(legend.title=element_blank()) +
  scale_color_manual(values=c("#FF6600", "#FFCC33", "#000066"))
```

```
PMCA2_exsp_exon <- subset(PMCA2_exon_mean, Subgroup %in% PMCA2_exon_tissue
s)
PMCA2_exsp_exon

ggplot(data = PMCA2_exsp_exon) +
  geom_jitter(mapping = aes(reorder(Subgroup, Order), y = Value, color = P
MCA2_exsp_exon$EventID), width = 0.1, height = 0.1) +
  theme_bw() +
  theme(axis.text.x = element_text(angle = 90, hjust = 1, vjust = 0.5)) +
  theme(legend.title=element_blank()) +
  scale_color_manual(values=c("#FF6600", "#FFCC33", "#000066")) +
  xlab('') + ylab('PSI') +
  ggsave("exons.png")

#------------------------------- The mean incorporation values for all tis
sues --------------------------###

exsp_asn <- filter(asn_CRPKMs_mean, CRPKMs > 0)
Event_mean_PMCA2 <- subset(PMCA2_exsp_exon, Subgroup %in% exsp_asn$Subgrou
p) %>%
group_by(EventID) %>%
summarise(mean_PSI = mean(Value),
            sd_PSI = sd(Value))

Event_mean_PMCA2

expression_interval <- c("0-10", "10-20", "20-30", "30-40", "40-50", "50-6
0", "60-70", "70-80", "80-90", "90-100", "100-110", "110-120", "120-130",
"130-140", "140-150", "150-160", "160-170", "170-180", "180-190")


# function for calculation of mean incorporation of a specific splicing ev
ent for tissues within a designated interval of aSN expression

Event_mean_int_PMCA <- function(PMCA_data){

interval <- seq(0, 190, by=10)
exsp_asn <- list()
Event_mean_PMCA <- list()
Event_mean_all_tissues <- list()
Event_mean_brain <- list()
Event_mean_PMCA_tissue <- list()

i <- 1
while (i < length(interval)) {


  exsp_asn[[paste(interval[[i+1]])]] <- filter(asn_CRPKMs_mean, CRPKMs >=
interval[[i]] & CRPKMs < interval[[i+1]])
  Event_mean_PMCA[[paste(interval[[i+1]])]] <- subset(PMCA_data, Subgroup
```

```
%in% exsp_asn[[i]]$Subgroup)

  Event_mean_all_tissues[[i]] <- Event_mean_PMCA[[paste(interval[[i+1]])]]
%>%
    summarise(n_group = n())

  Event_mean_brain[[i]] <- Event_mean_PMCA[[paste(interval[[i+1]])]] %>%
  filter(Group == "Neural_crest" | Group == "Neural" | Group == "NPC" | Gr
oup == "Glia" | Group == "EmbrBrain") %>%
    summarise(n_group = n())

Event_mean_PMCA_tissue[[paste(interval[[i+1]])]] <- Event_mean_PMCA[[paste
(interval[[i+1]])]] %>%
  filter(Value >= 0) %>%
  group_by(EventID) %>%
  summarise(mean_PSI = mean(Value),
            sd_PSI = sd(Value),
            n_tissues = n()) %>%
  add_column(percent_brain = sum(Event_mean_brain[[i]]$n_group)/sum(Event_
mean_all_tissues[[i]]$n_group)*100) %>%
  add_column(asn_exp_interval = interval[[i+1]])


  i <- i + 1

}

Event_mean_PMCA_tissue <- data.table::rbindlist(Event_mean_PMCA_tissue)

}

Event_mean_int_PMCA2 <- Event_mean_int_PMCA(PMCA2_exsp_exon)
Event_mean_int_PMCA2

Event_mean_int_PMCA2 %>%
  ggplot() +
  geom_point(mapping = aes(x = asn_exp_interval, y = mean_PSI, color = Eve
ntID), size = 2.5) +
  geom_errorbar(aes(x=asn_exp_interval, ymin=mean_PSI-sd_PSI, ymax=mean_PS
I+sd_PSI, width = 5))+
  theme_bw() +
  #theme(axis.text.x = element_text(angle = 90, hjust = 1, vjust = 0.5)) +
  theme(legend.title=element_blank()) +
  scale_color_manual(values=c("#FF6600", "#FFCC33", "#000066")) +
  labs(x = "aSN expression interval (CRPKMs)", y = "mean PSI")
```

```
theme(axis.text.x = element_text(angle = 90, hjust = 1, vjust = 0.5))

Event_mean_int_PMCA2 %>%
  ggplot() +
  geom_point(mapping = aes(x = asn_exp_interval, y = mean_PSI, color = Eve
ntID), size = 2.5) +
  #geom_errorbar(aes(x=asn_exp_interval, ymin=mean_PSI-sd_PSI, ymax=mean_P
```

```
SI+sd_PSI), width=0.25)+
  theme_bw() +
  #theme(axis.text.x = element_text(angle = 90, hjust = 1, vjust = 0.5)) +
  theme(legend.title=element_blank()) +
  scale_color_manual(values=c("#FF6600", "#FFCC33", "#000066")) +
  labs(x = "aSN expression interval (CRPKMs)", y = "mean PSI \n PMCA2 exon
s") +
  theme(axis.text.x = element_text(angle = 90, hjust = 1, vjust = 0.5)) +
  scale_x_continuous(breaks = seq(10, 190, by = 10), labels = expression_i
nterval)+
  theme(legend.position="bottom") +

  ggsave(width = 5, height = 4, filename = "mean_PSI_interval_PMCA2_2.png"
)
```

```
#t-test - calculate if there is a stastically significant difference betwe
en the mean PSI for tissues with aSN expression below 50 CRPKMs and above
50 CRPKMs.

interval_means <- function(PMCA_data, interval1, interval2) {
exsp_asn <- filter(asn_CRPKMs_mean, CRPKMs >= interval1 & CRPKMs < interva
l2)
  Event_mean_PMCA <- subset(PMCA_data, Subgroup %in% exsp_asn$Subgroup) %>
%
    group_by(EventID) %>%
    summarise(mean_PSI = mean(Value),
              sd_PSI = sd(Value),
              n_tissues = n())
}

# For PMCA2
low_data_means <- interval_means(PMCA2_exsp_exon, 0, 50)

high_data_means <- interval_means(PMCA2_exsp_exon, 50, 190)

# low_data_means
# high_data_means

tsum.test(mean.x = (low_data_means[[1,2]]), s.x = (low_data_means[[1,3]]),
n.x = (low_data_means[[1,4]]), mean.y = (high_data_means[[1,2]]), s.y = (h
igh_data_means[[1,3]]), n.y = (high_data_means[[1,4]]))

##
##  Welch Modified Two-Sample t-Test
##
## data:  Summarized x and y
## t = 4.9465, df = 18.681, p-value = 9.394e-05
## alternative hypothesis: true difference in means is not equal to 0
## 95 percent confidence interval:
##  22.96939 56.73360
## sample estimates:
```

```
## mean of x mean of y
## 42.673158  2.821667

## Adjusting tables for a more convenient look for publication

PMCA2exons <- Event_mean_int_PMCA2 %>% mutate(across(is.numeric, ~ round(.
, 2)))

PMCA2exons

aSN_exp_int <- c("0-10", "10-20", "20-30", "30-40", "40-50", "50-60", "80-
90", "120-130", "180-190")

PMCA2_adjusted_HsaEX0006891  <- PMCA2exons  %>%
  filter(EventID == "HsaEX0006891") %>%
  add_column(aSN_exp_int)

PMCA2_adjusted_HsaEX0006891[,c(7,2,3,4,5,1)]

PMCA2_adjusted_HsaEX0006892  <- PMCA2exons  %>%
  filter(EventID == "HsaEX0006892") %>%
  add_column(aSN_exp_int)

PMCA2_adjusted_HsaEX0006892[,c(7,2,3,4,5,1)]

PMCA2_adjusted_HsaEX0006893  <- PMCA2exons  %>%
  filter(EventID == "HsaEX0006893") %>%
  add_column(aSN_exp_int)

PMCA2_adjusted_HsaEX0006893[,c(7,2,3,4,5,1)]
```
