# Supplementary material for: Monomeric α‐synuclein activates the plasma membrane calcium pump
Source: EMBO J. 2023 Nov 2;42(23):e111122. doi: 10.15252/embj.2022111122 (PMC10690453; doi:10.15252/embj.2022111122)
Supplement: Supplementary file 2 — Expanded View Figures PDF [file EMBJ-42-e111122-s001.pdf]

Expanded View Figures

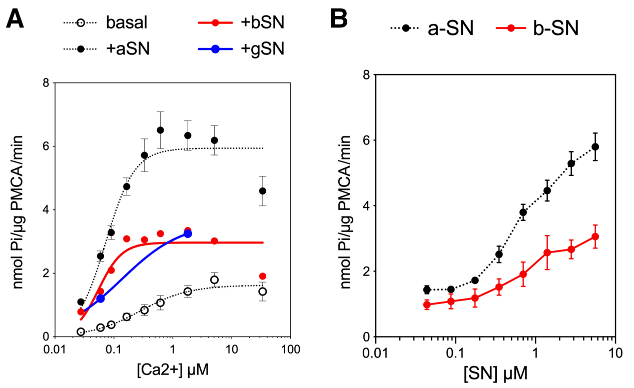

Figure EV1. PMCA1d activation by alpha-, beta-, and gamma-SN Beta- and gamma-synuclein stimulate PMCA but appear less potent than alpha-synuclein.

A Calcium titration measurement was performed with beta- (red) and gamma-synuclein (blue). PMCA1d was relipidated with brain lipid extract (BE). Data showing the activity without interaction partner and with alpha-synuclein are the same as in Fig 2, presented here for the comparison.

B Synuclein-titration experiment. Beta-synuclein titration was performed in presence of brain lipid extract (BE) in four technical replicates ($n = 4$). Data for alpha-synuclein titration are the same as in Fig 2, presented here for comparison. Data are mean ± SEM.

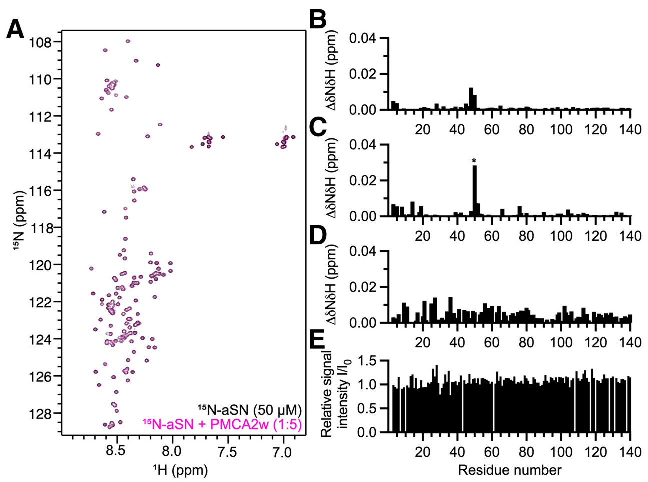

Figure EV2. NMR interaction studies.

A ^{15}N-HSQC spectra comparing ^{15}N-aSN alone (black) with that of ^{15}N-aSN added PMCA2w$_{A\text{-}TM3}$ loop (pink).

B Quantified amide chemical shift perturbations (CSPs) from (A).

C Amide CSPs caused by addition of PMCA2w$_{A\text{-}TM3}$ loop to ^{15}N-aSN in the presence of calcium (^{15}N-aSN:Ca^{2+} 1:500; ^{15}N-aSN:Ca^{2+}:PMCA2w 1:500:4). * indicates pH sensitivity observed for histidine.

D CSPs caused by full-length PMCA2w/a at a 1:1 molar ratio with ^{15}N-aSN.

E Peak intensity changes caused by addition of full-length PMCA2w/a at a 1:1 molar ratio with a^{15}N-aSN.

© 2023 The Authors
