# Supplementary material for: Monomeric α‐synuclein activates the plasma membrane calcium pump
Source: EMBO J. 2023 Nov 2;42(23):e111122. doi: 10.15252/embj.2022111122 (PMC10690453; doi:10.15252/embj.2022111122)
Supplement: Supplementary file 3 — Table EV1 [file EMBJ-42-e111122-s005.pdf]

Table EV1. VastDB Datasets used in the transcriptomics analysis.

Accession number	Link	Description
HsaEX0006882 @ hg19	https://vastdb.crg.eu/event/HsaEX0006882@hg19	Alternative splicing event of *ATP2B1* gene leading to PMCA1a variant
HsaEX0006897 @ hg19	https://vastdb.crg.eu/event/HsaEX0006897@hg19	Alternative splicing event of *ATP2B3* gene leading to PMCA3a variant
HsaEX0006904 @ hg19	https://vastdb.crg.eu/event/HsaEX0006904@hg19	Alternative splicing event of *ATP2B4* gene leading to PMCA4a variant
HsaEX0006891 @ hg19	https://vastdb.crg.eu/event/HsaEX0006891@hg19	Alternative splicing event of *ATP2B2* gene leading to PMCA2 variant containing Exon A1 (PMCA2w)
HsaEX0006892 @ hg19	https://vastdb.crg.eu/event/HsaEX0006892@hg19	Alternative splicing event of *ATP2B2* gene leading to PMCA2 variants containing Exon A2 (PMCA2w)
HsaEX0006893 @ hg19	https://vastdb.crg.eu/event/HsaEX0006893@hg19	Alternative splicing event of *ATP2B2* gene leading to PMCA2 variants containing Exon A3 (PMCA2w, PMCA2x)
ENSG00000145335 @ hg19	https://vastdb.crg.eu/gene/ENSG00000145335@hg19	Expression values of human *SNCA* gene, encoding alpha-synuclein
