# Supplementary material for: Monomeric α‐synuclein activates the plasma membrane calcium pump
Source: EMBO J. 2023 Nov 2;42(23):e111122. doi: 10.15252/embj.2022111122 (PMC10690453; doi:10.15252/embj.2022111122)
Supplement: Supplementary file 4 — PDF+ [file EMBJ-42-e111122-s003.pdf]

Article

TRANSPARENT PROCESS
OPEN ACCESS

THE EMBO JOURNAL

Monomeric α-synuclein activates the plasma membrane calcium pump

Antoni Kowalski[1,2,3,4,*,†], Cristine Betzer[2,5,‡], Sigrid Thirup Larsen[1,2], Emil Gregersen[2,5,§], Estella A Newcombe[3], Montaña Caballero Bermejo[1,2,6], Viktor Wisniewski Bendtsen[1,2], Jorin Diemer[7], Christina V Ernstsen[8], Shweta Jain[9], Alicia Espiña Bou[1,2], Annette Eva Langkilde[10], Lene N Nejsum[8], Edda Klipp[7], Robert Edwards[9], Birthe B Kragelund[3], Poul Henning Jensen[2,5,**] & Poul Nissen[1,2,***]

Abstract

Alpha-synuclein (aSN) is a membrane-associated and intrinsically disordered protein, well known for pathological aggregation in neurodegeneration. However, the physiological function of aSN is disputed. Pull-down experiments have pointed to plasma membrane Ca^{2+}-ATPase (PMCA) as a potential interaction partner. From proximity ligation assays, we find that aSN and PMCA colocalize at neuronal synapses, and we show that calcium expulsion is activated by aSN and PMCA. We further show that soluble, monomeric aSN activates PMCA at par with calmodulin, but independent of the autoinhibitory domain of PMCA, and highly dependent on acidic phospholipids and membrane-anchoring properties of aSN. On PMCA, the key site is mapped to the acidic lipid-binding site, located within a disordered PMCA-specific loop connecting the cytosolic A domain and transmembrane segment 3. Our studies point toward a novel physiological role of monomeric aSN as a stimulator of calcium clearance in neurons through activation of PMCA.

Keywords alpha-synuclein; calcium; calmodulin; plasma membrane Ca^{2+}-ATPase; presynapse
Subject Category Neuroscience
DOI 10.15252/embj.2022111122 | Received 14 March 2022 | Revised 19 September 2023 | Accepted 11 October 2023 | Published online 2 November 2023
The EMBO Journal (2023) 42: e111122

Introduction

The alpha-synuclein protein (aSN, 140 residues and 14.5 kDa molecular weight) is well recognized and highly studied for its pathological role in neurodegeneration. Oligomerization, fibrillization, and abnormal aggregation in neurons are linked to synucleinopathies, e.g., Lewy body dementia or Parkinson's disease (PD) (Burre *et al*, 2018; Oliveira *et al*, 2021). However, the normal function of native aSN remains an open question. Importantly, aSN is highly abundant in the presynaptic region of neurons, where concentrations reach 5–50 μM (Bodner *et al*, 2009; Theillet *et al*, 2016; Perni *et al*, 2017). It is likely involved in homeostasis of synaptic vesicle release (Sulzer & Edwards, 2019) although mechanisms remain unclear, but a chaperone function of SNARE proteins has been proposed (Burre *et al*, 2010). A tetrameric form was isolated from cells after *in vivo* cross-linking (Dettmer *et al*, 2013, 2015a); however, the intact aSN appears structurally disordered and monomeric in cells (Fauvet *et al*, 2012; Theillet *et al*, 2016). aSN is also a peripheral membrane protein adopting an α-helical structure of the positively charged N-terminal region 1–95 through interaction with acidic phospholipids (Dikiy & Eliezer, 2012). The membrane interaction of aSN was suggested to play a role in clustering of synaptic vesicles (Jo *et al*, 2000; Lautenschlager *et al*, 2018), regulation of the presynapse size (Vargas *et al*, 2017), and neurotransmitter release (Burre *et al*, 2010). A disease-related mutation A30P—strongly linked to the inherited form of PD—diminishes the lipid-binding properties

1 Department of Molecular Biology and Genetics, Aarhus University, Aarhus, Denmark
2 Danish Research Institute of Translational Neuroscience – DANDRITE, Aarhus University, Aarhus, Denmark
3 REPIN and Structural Biology and NMR Laboratory, Department of Biology, University of Copenhagen, Copenhagen, Denmark
4 Department of Molecular Neurochemistry, Medical University of Lodz, Lodz, Poland
5 Department of Biomedicine, Aarhus University, Aarhus, Denmark
6 Department Biochemistry and Molecular Biology and Genetics, IBMP, University of Extremadura, Badajoz, Spain
7 Theoretical Biophysics, Humboldt-Universität zu Berlin, Berlin, Germany
8 Department of Clinical Medicine, Aarhus University, Aarhus N, Denmark
9 Departments of Neurology and Physiology, University of California San Francisco, San Francisco, CA, USA
10 Department of Drug Design and Pharmacology, University of Copenhagen, Copenhagen, Denmark
*Corresponding author. Tel: +45 26785911; E-mail: antoni.kowalski@dandrite.au.dk
**Corresponding author. Tel: +45 28992056; E-mail: phj@biomed.au.dkphj@mbg.au.dk
***Corresponding author. Tel: +45 28992295; E-mail: pn@mbg.au.dk
†Present address: ImmunAware ApS, Hørsholm, Denmark
‡Present address: Region Midtjylland, Regionshospitalet Gødstrup, Herning, Denmark
§Present address: Department of Clinical Medicine, Aarhus University, Aarhus N, Denmark

© 2023 The Authors. Published under the terms of the CC BY 4.0 license

(Jensen *et al*, 1998). The C-terminal region, comprising residues 96–14, appears as constitutively disordered, has a strong negative charge, and binds calcium ions with low affinity (Eliezer *et al*, 2001; Nielsen *et al*, 2001; Lautenschlager *et al*, 2018).

Regulation of intracellular calcium homeostasis is essential for the proper functioning of cells. Resting concentration of free Ca^{2+} in the cytosol of a healthy cell is around 100 nM, while the extracellular concentration is in the millimolar range. In a calcium signaling event, rapid calcium influxes via calcium channels increase intracellular calcium concentrations and must be followed by efficient recovery by specialized proteins. The key players are (i) the plasma membrane calcium ATPase (PMCA) and (ii) the sodium-calcium exchanger (NCX), both removing Ca^{2+} to the extracellular space, and (iii) the sarco/endoplasmic reticulum Ca^{2+}-ATPase (SERCA) filling intracellular calcium stores. Furthermore, calcium-binding proteins, in particular calmodulin (CaM), function as a calcium buffer and calcium-dependent regulators of multiple proteins (Cali *et al*, 2018).

Increasing evidence reveals a link between calcium dysregulation and propagation of synucleinopathies. Voltage-gated Ca_V1 channels and $Ca_V1.3$ mRNA are upregulated as an early feature of PD in areas not associated with overt loss of neurons or Lewy body formation (Hurley *et al*, 2013, 2015). Fibrillar oligomers of aSN, which are formed with the disease development, can enhance the Ca^{2+}-permeability of plasma membrane (Cali *et al*, 2014; Di Scala *et al*, 2016; Rcom-H'cheo-Gauthier *et al*, 2016) and have been found to activate SERCA (Betzer *et al*, 2018), thus contributing to disturbances in calcium homeostasis. The SERCA interaction was identified by aSN pull-down experiments, and PMCA was also identified from these experiments (Betzer *et al*, 2015).

Neurons have a large complexity and a highly polarized architecture; calcium signaling events in these cells, therefore, are highly localized. Dramatic, but extremely local calcium influxes are related to signals leading to vesicular exocytosis in the presynaptic termini. In the presynaptic bouton, the signaling events take place in nanodomains within 50 nm from Ca^{2+} channels (Augustine *et al*, 2003) and free calcium concentrations can rise locally by > 1,000-fold (Long *et al*, 2008). Terminating rather than dissipating a local signal causes a high demand for an efficient and flexible calcium removal system with fine-tuning to a required level of resting concentrations. PMCA plays a key role in this process.

PMCA is a transmembrane protein of 130–140 kDa and belongs to the P2B subfamily of P-type ATPases. Being a high affinity and low-capacity active transporter, it can fine-tune the resting free calcium ion concentration in cytosol (Carafoli, 1994; Strehler *et al*, 2007b). In humans and other mammals, four PMCA isoforms (PMCA1-4) are encoded by separate genes (Strehler *et al*, 2007a). PMCA1 is considered a "housekeeping" pump and together with PMCA4 ubiquitously expressed in all tissues. PMCA2 and 3 have specific expression patterns and are mostly found in excitable tissues and often referred to as neuron-specific isoforms (Domi *et al*, 2007; Strehler & Thayer, 2018). Through alternative RNA splicing at two different sites ("A" and "C"), the four isoforms can be produced in more than 20 different variants (Strehler & Zacharias, 2001). Splicing at site C affects the length of the C-terminal tail with CaM-binding sites (variants denoted a through d), and splicing at site A impacts the length of the first intracellular loop (variants denoted w, x, y, z), which leads from the A-domain to the transmembrane segment 3 (TM3) (Strehler, 2015). The different splice variants are in many cases tissue-specific and differ in the degree of activation and autoinhibition (Kessler *et al*, 1992; Caride *et al*, 1999).

PMCA is regulated through interactions with protein partners as well as phospholipids. A classical regulatory feedback mechanism happens via the C-terminal autoinhibitory domain of PMCA. In the resting cell, the domain interacts with the cytoplasmic domains and autoinhibits calcium transport. Upon an increase in intracellular calcium, calcium-bound CaM binds to the autoinhibitory domain resulting in a rise in PMCA activity. The degree and calcium threshold of activation may depend on how many CaM-binding sites—one or two—are present within the autoinhibitory domain (Tidow *et al*, 2012). Furthermore, PMCA regulation by acidic phospholipids has been mapped to two binding sites—one in the autoinhibitory domain, the other at the cytosolic loop between the A-domain and TM3. The mechanism of how acidic lipids regulate the pump is not understood in detail; however, they appear to be both modulators of activation by CaM as well as stand-alone activators (Niggli *et al*, 1981a; Zvaritch *et al*, 1990; Brodin *et al*, 1992; Pinto Fde & Adamo, 2002; Tidow *et al*, 2012; Lopreiato *et al*, 2014; Penniston *et al*, 2014). Hence, the (auto)regulatory regions also correspond to the sites of variations by alternative splicing.

Here, we show that aSN in its soluble, monomeric form acts as a very potent activator of human PMCAs. The effect relies on the presence of acidic phospholipids and is independent of the CaM-binding autoinhibitory domain. Our findings suggest that the activation mechanism is based on interactions of the N-terminal segment of aSN, negatively charged lipids, and a phospholipid binding site of PMCAs. We propose that aSN complements CaM in local compartments such as the presynapse and that aSN unlike CaM provides a pool of activated PMCA through a Ca^{2+}-independent mechanisms that can respond immediately to calcium spikes. The findings provide a new perspective on the physiological role of native aSN in the presynapse and demonstrate how the lipid environment affects the critical calcium extruding activity of PMCA.

Results

aSN co-localizes with PMCA and stimulates calcium extrusion

Co-immunoprecipitation experiments with brain homogenate from aSN-knockout mice supplemented with purified aSN monomer revealed aSN binding, and a similar experiment with aSN oligomer showed the same result indicating no discrimination on aSN conformation (Fig 1A, left). Co-immunoprecipitation and western blotting of endogenous aSN and PMCA from detergent extracts of total brain homogenates from *wild-type* C57BL/6 mice show that endogenous PMCA interacts with endogenous aSN (Fig 1A, right). PMCA appears with multiple bands, or a smear on western blots from the two co-IPs, likely due to the varying amounts of post-translational modifications of PMCA (Tolosa de Talamoni *et al*, 1993). Moreover, a pull-down assay performed with purified proteins showed aSN binding to PMCA immobilized on Calmodulin sepharose (Fig 1B).

The interaction between aSN and PMCA was investigated further in primary hippocampal neurons derived from newborn C57BL/6 mice and with neurons from aSN-KO mice as negative controls. Primary hippocampal neurons were fixed and analyzed after 14 days

 © 2023 The Authors

in culture by immunofluorescence labeling of synapses by synaptophysin and an aSN/PMCA proximity ligation assay (PLA). The PLA is used for *in situ* detection of protein interactions and is based on two primary antibodies from different species, here ASY-1, a polyclonal aSN antibody generated in rabbit, and 5F10, a monoclonal pan PMCA antibody generated in mouse. The secondary antibodies were labeled with oligonucleotides that enzymatically can be ligated when in proximity and then amplified, hence generating concatemeric sequences. These sequences can bind fluorescently labeled oligonucleotides yielding a red fluorescent signal when aSN and PMCA are located within approximately 40 nm. PMCA and aSN were found to be in this proximity of each other and located to the synapses (Fig 1C).

The functional effect of PMCA calcium transport was investigated in primary hippocampal neurons from aSN-KO neurons transiently transfected with either aSN and mCherry, or mCherry alone as negative control. SERCA was inhibited by thapsigargin (4 μM) before recording. Cellular calcium responses were monitored by the calcium-sensing dye Fura2-AM upon depolarization by 8 mM KCl in the extracellular medium. Neurons expressing aSN expelled calcium to a markedly higher degree than the mCherry expressing neurons (Fig 1D and E). Vanadate is a well-known and potent inhibitor of

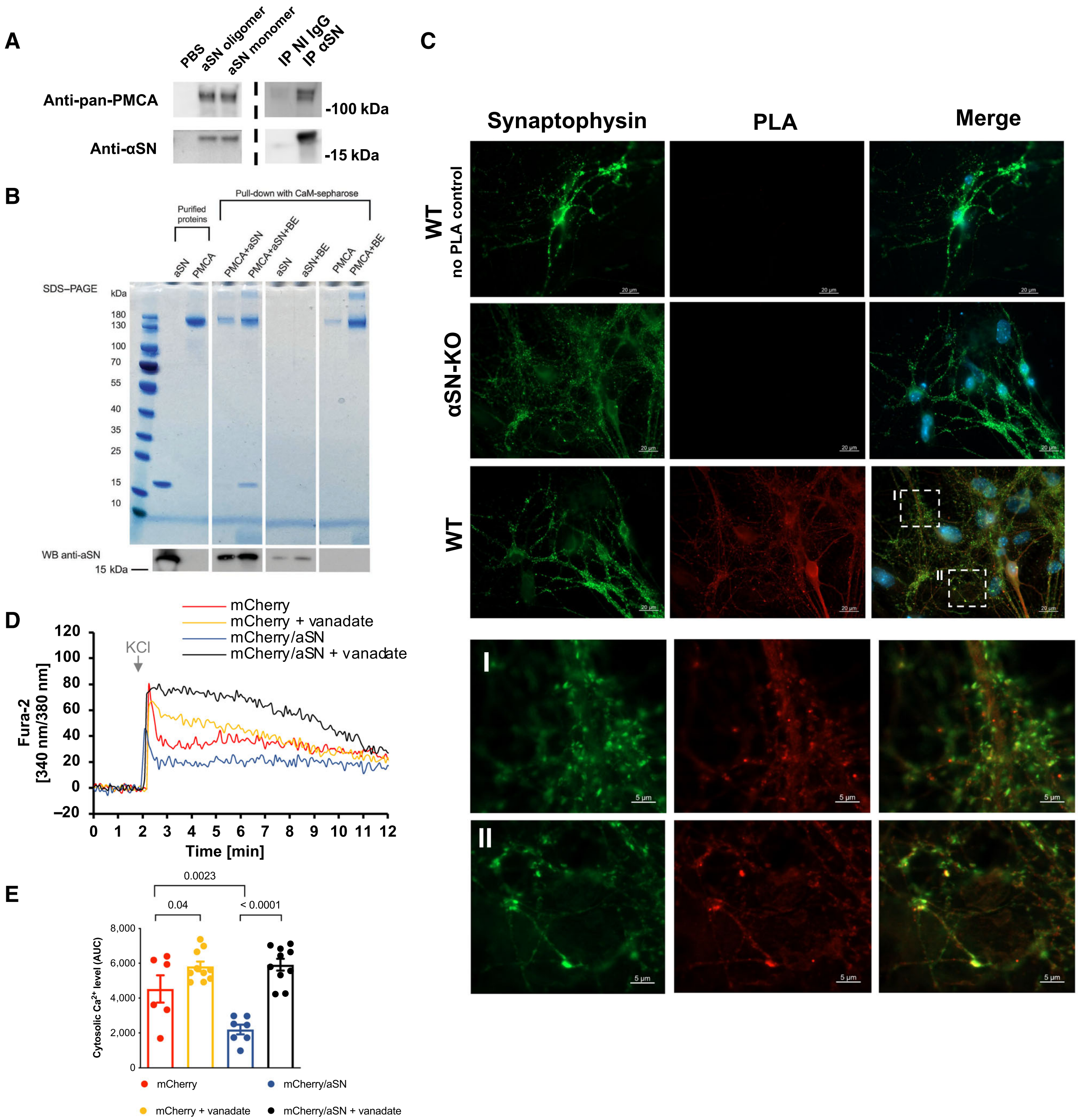

Figure 1.

◀ **Figure 1. aSN co-localizes with PMCA and stimulates calcium extrusion.**

A Co-immunoprecipitation of PMCA with aSN. *Left*: In detergent extracts of total brain homogenates from aSN-knockout (aSN-KO) mice, PMCA is co-immunoprecipitated by both exogenous aSN monomer and *in vitro* formed aSN oligomers using aSN binding sepharose (ASY-1). Negative control without exogenous aSN (PBS) confirms no unspecific antibody binding. *Right*: In detergent extracts of total brain homogenate from C57BL/6 mice, endogenous aSN is pulled down together with the endogenous PMCA by aSN specific antibody (ASY-1) and not by control non-immune antibody (NI IgG). Cropped blots presented here are representative. The experiment was performed in triplicates. Images of full blots are presented in the Appendix Fig S6A–D.

B Pull-down assay of aSN with PMCA immobilized on Calmodulin Sepharose 4B. PMCA was preincubated with aSN (1:30 molar ratio) in absence or presence of brain lipid extract (BE). Coomassie stained SDS–PAGE analysis (*top*, *colored image*) shows aSN co-elutes with PMCA. Low level of unspecific binding of aSN to CaM-sepharose beads was not detected by Coomassie staining, but was detectable by western blotting (*bottom*, *black-and-white image*). Full images of Coomassie-stained SDS–PAGE gel and western blots are presented in the Appendix Fig S7A–C.

C Proximity ligation assay (PLA) of aSN and PMCA primary hippocampal neurons. PLA of PMCA and aSN in primary hippocampal neurons made from WT or aSN-KO mice show that the proteins *in situ* are in close proximity. *Left column*: synapses visualized by synaptophysin labeling. *Middle column*: red fluorescence signal represents positive PLA result, meaning proximity of aSN and PMCA of $<$ 40 nm. *Right column*: Merged PLA and synaptophysin images show PMCA and aSN interaction localized to the synapses. Scalebar 20 μm. *I and II*: Zoomed-in images of boxed areas of WT primary hippocampal neurons. Scalebar 5 μm.

D, E aSN increases calcium export from depolarized primary neurons. Cytosolic calcium was monitored by Fura-2-AM loaded into DIV8 neurons. Before recording, SERCA was inhibited by thapsigargin. Calcium influx was induced by the addition of KCl. At the recording time of 2 min., KCl was added to depolarize the neurons and the calcium response was followed over time. (D) Representative curves of single neurons expressing; *blue*: mCherry and aSN, *black*: mCherry and aSN treated with 1 μM vanadate to inhibit ATPases, *red*—mCherry alone, *yellow*—mCherry alone and treated with 1 μM vanadate. (E) Cytosolic Ca^{2+} level after the KCl-induced influx, quantified as the area under the curve (AUC ± SEM). The response upon K^+ induced depolarization was quantified as the Area Under Curve (AUC) from each measured neuron in the 2–12 min. interval. N (mCherry/aSN) = 7, N (mCherry/aSN + vanadate) = 10, N (mCherry) = 6, and N (mCherry + vanadate) = 10. The colors of the bars correspond to the top figure. Data presented as mean ± SEM. Statistical analysis is conducted as multiple comparisons with one-way ANOVA combined with Sidak *post hoc* test.

P-type ATPases (Cantley *et al*, 1978; Bond & Hudgins, 1980; Dupont & Bennett, 1982) and was used in this experiment as a PMCA inhibitor, since SERCA was inhibited by thapsigargin from the start. Vanadate decreased the calcium expulsion of aSN mCherry neurons to the same level as mCherry-only neurons treated with vanadate. This supports that increased efflux induced by aSN is driven by PMCA.

Calcium extrusion experiments similar to those in neurons were also attempted in three different cell lines, SH-SY5Y, PC12, and Chromaffin cells, to investigate further variations of background conditions. In the human neuroblastoma cell line SH-SY5Y, with doxycycline controllable wild-type aSN expression, the response to K^+ dependent depolarization had great variability, as have been observed previously (Morton *et al*, 1992). For cells with a clear response to K^+ dependent depolarization, the SH-SY5Y cells overexpressing aSN exhibited increased calcium expulsion compared to SH-SY5Y with no aSN expression (Appendix Fig S1A–C). In PC12 cells, the response to K^+-dependent depolarization gave a good signal, but no difference between control cells and cells overexpressing aSN was observed (data not shown). This can have many causes, but we did not explore it further. For Chromaffin cells, we did not detect a significant response to K^+-dependent depolarization. Hence, in particular primary hippocampal neurons, and to some extend also SH-SY5Y cells with inducible overexpression of wild-type aSN, show aSN-stimulated PMCA activity.

Monomeric aSN activates human PMCA in a lipid-dependent manner and independent of the autoinhibitory domain of PMCA

The measurements of calcium-dependent ATPase activity of PMCA were performed with two human PMCA—ubiquitous PMCA1 and neuron-specific PMCA2 isoforms (specifically the splice variants PMCA1d and PMCA2w/a). The experiments were performed with PMCAs relipidated either with porcine brain phosphatidylcholine (BPC, neutral lipids) or with bovine brain lipid extract Folch fraction I (Sigma-Aldrich, from here on referred to as brain extract lipids, BE), of which 60% are negatively charged phosphatidylinositol and phosphatidylserine lipids (Folch *et al*, 1957; Boura & Hurley, 2012). We observed monomeric aSN causing a strong increase in the activity of both PMCA isoforms, however only for PMCAs relipidated with BE, not BPC. Ca^{2+} titration revealed that the elevation of ATPase activity is also accompanied by a three- to five-fold increase in the apparent calcium affinity (Fig 2A, left, center; Table 1). Additionally, the activation is gradually abolished by increasing the content of neutral BPC in the relipidation mixture (Fig 2A, right). To rule out potential, unspecific effects of other ATPases or impurities in the PMCA preparations, we prepared an inactive variant of PMCA ($PMCA1d_{D475N}$), in which the reactive aspartyl residue in the active site was replaced by asparagine. In terms of purity, these preparations were no different from the wild type, as confirmed by SDS–PAGE and size exclusion chromatography (Appendix Fig S2A–D), and they showed no ATPase activity, neither with nor without aSN (Fig 2A left, 2B).

To further investigate the acidic phospholipid dependence, we performed aSN titration comparing effects of wild-type aSN and the A30P variant with reduced membrane-binding ability (Jensen *et al*, 1998). In BE, the activation of PMCA was significantly attenuated by the A30P mutation. As could be expected, the aSN variant was ineffective in the neutral environment of BPC (Fig 2B).

Next, we examined the potential interplay between aSN and the well-known protein activator of PMCA, namely Calmodulin (CaM). For both PMCA isoforms, the susceptibility for activation by CaM and aSN appears to be lipid-dependent, with the acidic lipids promoting the effect of aSN and neutral lipids promoting the effect of CaM (Fig 2C). To confirm that the action of aSN was independent of the autoinhibitory domain, we designed a truncated construct (PMCA1-ΔC), lacking 184 residues of the C-terminus including the autoinhibitory domain with the CaM-binding site(s) and a long, intrinsically disordered linker region (Fig 2D, top). Foreseeably, loss of the C-terminal tail resulted in a constitutively active protein, insensitive to CaM (Appendix Fig S3A) and with basal activity higher than that of the full-length wild type (Fig 2D, bottom left). However, the stimulation by aSN was preserved and appeared like for the full-length pump, indicating the aSN effect to be independent

 © 2023 The Authors

of the autoinhibitory mechanism associated with the C-terminal tail (Fig 2D, bottom right). The apparent Ca^{2+} affinity of PMCA1d was increased by the truncation itself, and aSN strengthened that effect (Table 1). Again, the activation was lipid-dependent, occurring in the PMCA1-ΔC variant reconstituted in BE and not in BPC (Appendix Fig S3A).

Additionally, we examined the oligomeric form of the full-length aSN, which was previously established to have a great activating effect on SERCA (Betzer *et al*, 2018). For PMCA, however, the effect of oligomers was smaller than that of the monomer, occurring only at higher concentrations and to a lower fold of activation (Appendix Fig S3B). The activation might simply be explained by dissociating monomer from the oligomer, which we have found to be present in oligomer preparations (Appendix Fig S4). Moreover, we measured the PMCA activity in presence of two other synuclein isoforms, beta- and gamma-synuclein. Both stimulated the pump, but to a lesser extent (Fig EV1A and B).

PMCA alternative splicing events correlate with aSN expression level

PMCA is extensively regulated by alternative splicing, which results in over 20 variants, differing in distribution, kinetic properties, and CaM sensitivity (Strehler & Zacharias, 2001; Krebs, 2015). We

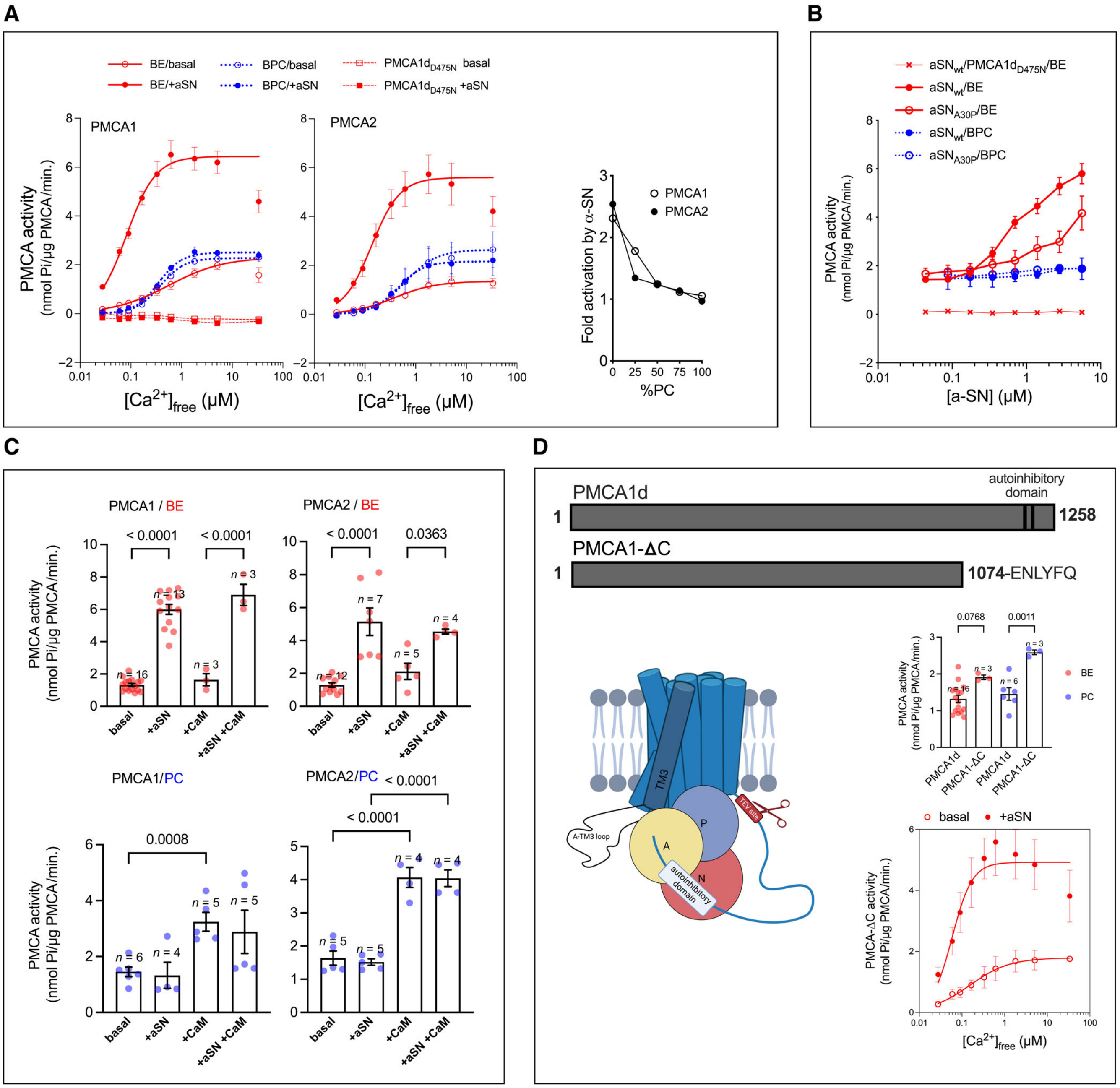

Figure 2.

Figure 2. Monomeric alpha-synuclein activates human PMCA in a lipid-dependent and autoinhibitory domain-independent manner.

A aSN stimulates plasma membrane calcium ATPase activity in an acidic, but not neutral lipid environment. *Left and center*: aSN activates PMCA1d and PMCA2w/a in the presence of brain extract (BE, *red solid line*), but not in presence of brain PC (BPC, *blue dotted line*). PMCA activity was measured as a function of free Ca^{2+} concentration ($[Ca^{2+}]_{free}$). *Empty symbols* represent basal activity and *filled symbols* the activity in presence of 5.6 μM aSN. The lines are the best fit given by the Hill equation with K_d values listed in Table 1. To rule out nonspecificity of ATP hydrolysis observed in wild-type PMCA, an inactive D475N mutant of PMCA1d was tested in presence of BE (*squares*, *dashed red line*). Data in the calcium titration experiment are mean ± SEM; for some points, the error bars are smaller than symbols. Measurements were performed in at least triplicates, and PMCA1d and PMCA2w/a originated from three or more independent expression cultures, except the inactive mutant, which was expressed one time. *Right*: Fold activation of PMCAs by aSN decreases with the increasing amount of neutral lipids. Brain PC was titrated in BE. *Empty symbols*—PMCA1d, *filled symbols*—PMCA2w/a. Data are from a single experiment for each of the PMCA isoforms.

B Compared to wild-type, A30P aSN has reduced ability to activate PMCA in the presence of acidic lipids. PMCA1d activity was measured as a function of aSN (wild-type or A30P) concentration in presence of 1.8 μM free Ca^{2+}. PMCA1d was relipidated either with BE (*red*) or BPC (*blue*). Neither of the aSN variants activated PMCA in a neutral lipid environment. The inactive PMCA1d (D475N) was not stimulated by aSN in the presence of BE. Data are mean ± SEM. For some of the points, the error bars are smaller than the symbols. Measurements were performed in at least triplicates, and PMCA1d originated from three or more independent expression cultures, except the inactive mutant, which was from single expression culture.

C Neutral or acidic lipids create a condition for PMCA being activated exclusively by CaM or aSN. Bars display the PMCA1d (*left*) and PMCA2w/a (*right*) activity without activator, with aSN, with CaM, or both. Dots represent individual measurements. PMCA was relipidated in BE (*top*, *red dots*) or BPC (*bottom*, *blue dots*). *P* values were obtained with ordinary one-way ANOVA with multiple comparisons.

D The activation by aSN is independent of PMCA's autoinhibitory domain. *Top*: Comparison of two PMCA1 variants—full-length PMCA1d with CaM-binding sites and PMCA1-ΔC, where the C-terminal tail, containing the autoinhibitory domain with CaM-binding sites, was removed by cleavage with TEV protease. TEV proteolytic cleavage site was introduced by site-directed mutagenesis at residue 1,074. *Bottom left*: Schematic representation of PMCA1d with TEV cleavage site introduced by site-directed mutagenesis. *Middle right*: Basal activity of PMCA1d and PMCA1-ΔC in presence of either BE or BPC. Dots represent individual measurements. *P* values were obtained with ordinary one-way ANOVA with multiple comparisons. *Bottom right*: The activity of PMCA1-ΔC with (*filled symbols*) and without 2.8 μM aSN (*empty symbols*) as a function of increasing $[Ca^{2+}]_{free}$. PMCA1-ΔC was relipidated with BE. The lines are the best fit given by the Hill equation with K_d values listed in Table 1. Except for cases of Ca^{2+} or aSN titration, the fixed concentrations were: $[Ca^{2+}]_{free}$: 1.8 μM or aSN: 5.6 μM. Relevant no-calcium or no-aSN backgrounds were subtracted. Data are expressed as mean ± s.e.m. Calculation of K_d values and statistical analysis was conducted in GraphPad Prism. Subfigure 2D (*bottom left*) as well as 3C, 3D (*bottom right*), 4A, 4C, and 4E were created with Biorender.com.

Table 1. $K_d^{app}(Ca^{2+})$ values (μM) of PMCAs in different lipid environments. Value ± SEM given by the Hill equation.

$K_d^{app}(Ca^{2+})$, microM	Brain lipid extract (BE) basal	Brain lipid extract (BE) + aSN	Brain PC basal	Brain PC + aSN
PMCA1d	0.56 ± 0.02	0.082 ± 0.005	0.38 ± 0.02	0.35 ± 0.02
PMCA1-ΔC	0.16 ± 0.01	0.06 ± 0.01	0.11 ± 0.01	0.10 ± 0.01
PMCA2w/a	0.38 ± 0.05	0.13 ± 0.01	0.62 ± 0.03	0.44 ± 0.02

wondered if PMCA splice variants could vary also in how they respond to aSN. We used available transcriptomics data from the VastDB database, http://vastdb.crg.eu/ (Tapial *et al*, 2017), containing information on alternative splicing events. The level of alternative splicing events in different tissues are indicated by PSI values ("percent spliced in"), and we asked whether specific splicing events correlate with aSN expression levels. First, we analyzed the splice site C, which is located at the C-terminal region of PMCA and contains the autoinhibitory domain that interacts with CaM. Alternative splicing at this site gives variants that may differ in the degree of autoinhibition or rate of activation by CaM. The splice variant "a," with a shortened autoinhibitory domain, is known to have higher basal activity and to be less sensitive to CaM stimulation (Caride *et al*, 2007). The available data regarded PMCA1, PMCA3, and PMCA4, and the analysis displayed in Fig 3A shows that in the case of PMCA1 and PMCA4, incorporation leading to the variant "a" positively correlates with the expression level of aSN with only a few outliers. Only PMCA3 did not correlate and the PSI value of the "a" variant was relatively high regardless of aSN expression interval. The results suggest that in tissues expressing aSN at high levels, PMCA is alternatively spliced in a way that favors less CaM-sensitive variants. The PMCA "a" variant is mainly expressed in brain tissues and indicates a positive correlation between PMCA and aSN. Besides brain tissues, aSN is also expressed in high amounts in melanocytes and bone marrow, which account for the two outliers in the plot.

The other splice site—termed A—is located at the loop between the A-domain and TM3 (from here on referred to as "A-TM3 loop"). This loop is much longer in PMCAs compared to other P-type ATPases. Among isoforms, PMCA1 is not being spliced in this region, while in PMCA2 alternative exon composition leads to four different variants named "z," "x," "y," and "w," with all of them except "y" having been detected in humans (Strehler *et al*, 2007b; Di Leva *et al*, 2008). Figure 3B displays the analysis of the PMCA2 splicing events, where three exons can be incorporated leading to variants "w" (full length containing sequences marked in orange, yellow, and blue) and "x" (containing the blue sequence). The "z" variant occurs when none of the three exons are incorporated (Fig 3C). We observed incorporation of the exons leading to variants with a longer A-TM3 loop insert in tissues with low aSN expression and barely any incorporation of these exons for tissue with high expression of aSN. This suggests that low aSN expressing tissues would preferentially transcribe the longest PMCA2w, and tissues richer in aSN would express more PMCA2x and z.

The extended, variable A-TM3 loop of PMCA is predicted to be structurally disordered; a trait which was confirmed using circular dichroism (CD) spectroscopy analysis of a recombinantly expressed $PMCA2w_{A\text{-}TM3}$ loop (Fig 3D). Near TM3, the loop contains several positively charged residues that mediate the activation by acidic phospholipids (Brodin *et al*, 1992; Pinto Fde & Adamo, 2002; Brini *et al*, 2010). We speculated about the importance of the loop length in the interaction with disordered aSN and performed the free calcium titration experiment with PMCA2 variants w/a, x/a, and z/a. All PMCA2 constructs used in the experiment were transformed anew into yeast cells derived from one colony and were expressed,

 © 2023 The Authors

purified, and tested simultaneously. The activity assays show that all three variants are activated by aSN (Fig 3E). The most remarkable difference is that PMCA2w/a has the lowest maximum activity among variants when activated by aSN. It was also the only one with a substantial increase in the apparent calcium affinity induced by aSN, the other having high affinity also in the basal activity state. PMCA2z/a, having the shortest loop region, showed the highest basal activity. The results implicate the A-TM3 loop in aSN interactions.

Indirect interaction between aSN and PMCA occurs between the N-terminal region of aSN and the acidic phospholipid binding site of PMCA

To determine whether the activation of PMCA was triggered by a direct protein–protein interaction of aSN with the $PMCA_{A\text{-}TM3}$ loop, we performed interaction studies using NMR spectroscopy. We incubated recombinant $PMCA2w_{A\text{-}TM3}$ loop with ^{15}N-labeled aSN, however observing no chemical shift perturbations (CSPs; Fig EV2A), in

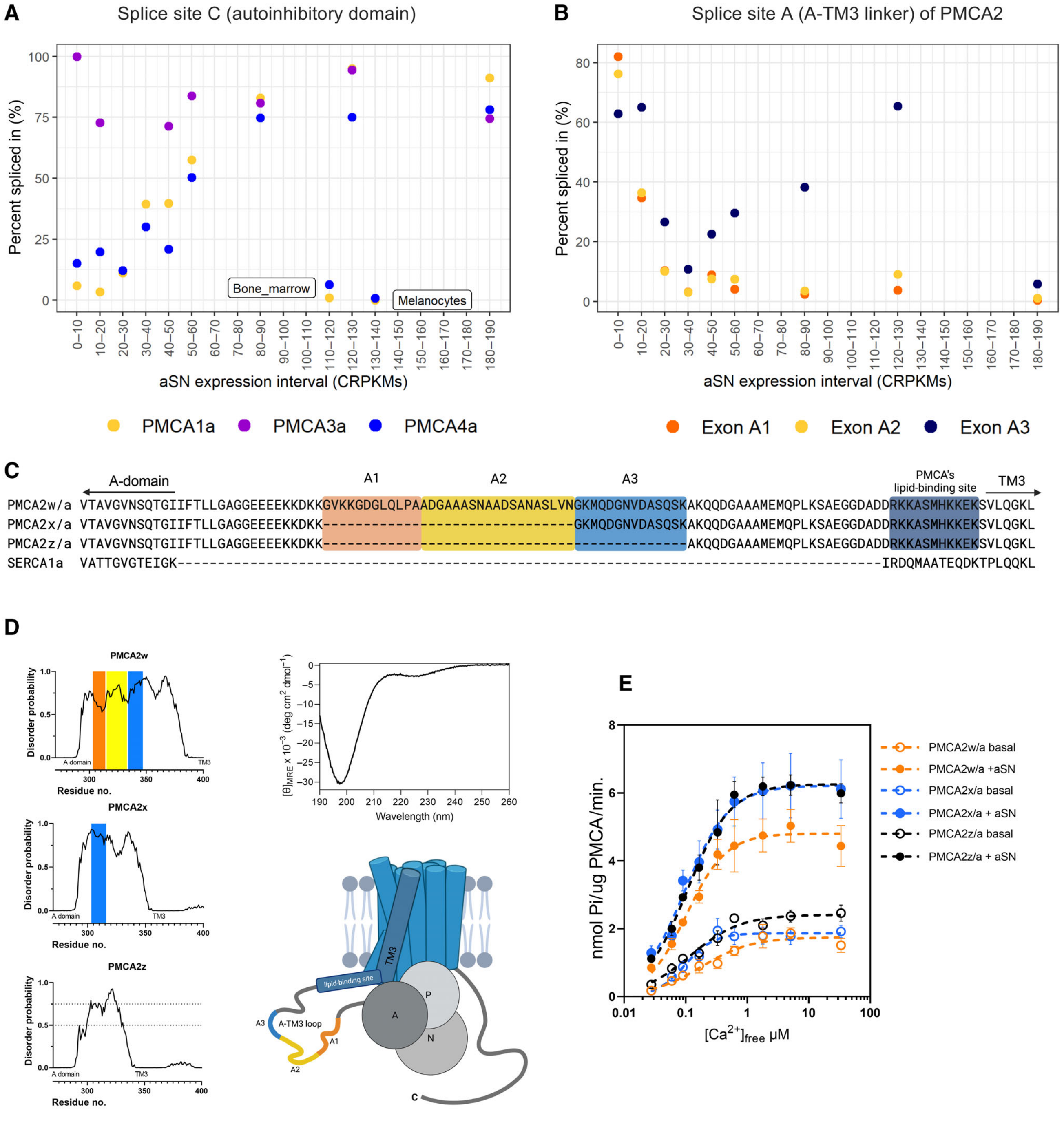

Figure 3.

Figure 3. Alternative splicing events of PMCA correlate with aSN expression levels and can modulate activating effect of aSN.

A, B PMCA's alternative splicing events correlate with aSN expression levels. The analysis of transcriptomics data from the VastDB database. (A) Exon incorporation for exons at splice site C for PMCA 1, 3, and 4. The average value of percent spliced in (PSI) for the exon leading to splice variant "a" as a function of aSN tissue expression. Expression levels are provided using cRPKM metric (corrected reads per kilobase of target transcript sequence per million of total reads). (B) Exon incorporation for exons at splice site A for PMCA2. The average value of PSI for the three possible exons as a function of aSN tissue expression. The color code for the exons (orange, yellow, blue) is consistent with corresponding PMCA regions marked on the subfigures (C) and (D). For VastDB, entry numbers of analyzed events refer to the method section.

C Sequence alignment of the extended A-TM3 loop of PMCA2w, x, and z variants. Fragments A1, A2, and A3 of the A-TM3 loop correspond to alternative exons incorporations. Color code corresponds to respective alternative splicing events presented in fig B. Sequence of the corresponding region of SERCA1a for comparison.

D Structural disorder in the A-TM3 loop of PMCA2. Disorder predictions: *Top left*—PMCA2w, *middle left*—PMCA2x, *bottom left*—PMCA2z, and *top right*—far-UV CD analysis of PMCA2w$_{A\text{-}TM3}$ loop with minimum below 200 nm reflecting a high degree of disorder. *Bottom right*—schematic representation of PMCA2w/a with the intrinsically disordered loop region between domain A and TM3.

E Comparison of aSN impact on three splice variants of human PMCA2. Ca^{2+}-dependent activity of PMCA2w, x, and z, with and without 5.6 μM aSN. Measurements were performed in four replicates. PMCA proteins used in the study were prepared in a manner, where transformation, expression culture, and protein purification were performed in parallel. Purification is reported in the Appendix Fig S2C. The lines are the best fit given by the Hill equation with the apparent K_d values (μM) for Ca^{2+} as follows: PMCA2w/a basal (without aSN)—0.18 ± 0.04, with aSN 0.102 ± 0.009; PMCA2x/a basal—0.10 ± 0.01, with aSN 0.097 ± 0.009; PMCA2z/a basal 0.13 ± 0.02, with aSN 0.106 ± 0.008.

both the absence (Fig EV2B) and presence of Ca^{2+} (Fig EV2C). Further, we found no indications of a direct interaction between full-length PMCA2w/a and aSN, as no CSPs (Fig EV2D) or changes in NMR peak intensities (Fig EV2E) could be seen in the aSN spectra. This suggests the interaction between aSN and PMCA is dependent on a more complex environment and likely is occurring via a mechanism facilitated by the membrane.

To map the potential interaction site on the aSN sequence (1–140), we performed a comparison of shortened aSN variants, truncated N-terminally (Δ14, Δ29) to compromise membrane binding (Cholak *et al*, 2020) or C-terminally (1–95, 1–61) to compromise low-affinity calcium binding and to remove the NAC binding region (Skaanning *et al*, 2020) respectively (Fig 4A). Most notably, the Δ14 variant had a greatly weakened effect on PMCA and Δ29 had almost lost its ability to activate PMCA, displaying only minor effects above 5 μM concentration. The C-terminally truncated (1–95) variant generally maintained function although to a lesser extent, activating to a lower fold (Fig 4B, Appendix Fig S3C). The (1–61) variant of aSN did not reach saturation within the examined concentration range, which suggests greatly reduced apparent affinity to the PMCA association.

The disordered C-terminal tail of aSN has calcium-binding properties (Lautenschlager *et al*, 2018) with a relevant affinity of ~ 20–50 μM (Newcombe *et al*, 2021), and thus we asked if it would impact the apparent calcium affinity of PMCA. Here, we compared activation of PMCA by the full length and C-terminally truncated (1–95) aSN in the Ca^{2+} titration experiment. However, despite a lower-fold activation, the C-truncated construct did not differ in shifting PMCA's apparent K_d for Ca^{2+} (Appendix Fig S3C).

Within certain limitations, deletions of large fragments of the lengthy A-TM3 loop of PMCA, including its binding site for acidic, negatively charged lipids, can retain basal activity (Pinto Fde & Adamo, 2002). Guided by this, we designed constructs and expressed and purified deletion variants of PMCA2, where we removed large parts of the loop. Sequences of the targeted region of PMCA2w/a and three designed variants (Δ292–383, Δ298–383, Δ298–372) are displayed in Fig 4C. The activity assay in the presence of BE (Fig 4D) showed differences in the basal activity and response to aSN. We observed a complete loss of ATPase activity of the Δ292–383 variant. Keeping the six N-terminally located residues of the loop resulted in the Δ298–383 variant with a basal activity preserved (0.84 ± 0.02 nmol Pi/μg PMCA/min.), but not response to aSN titration. Preserving further the 11-residue-long binding site for negatively charged lipids at the C-terminal end of the loop close to TM3 (Δ298–372) boosted basal activity (3.4 ± 0.5 nmol Pi/μg PMCA/min) and reinstalled the activation by aSN to the level observed in wild type PMCA2w/a (which has however a lower basal activity of 2.3 ± 0.3 nmol Pi/μg PMCA/min). This points to the binding site for negatively charged lipids as crucial for the interaction with aSN, and we propose the aSN-PMCA interaction to be mediated by acidic phospholipids involving the N-terminal region (1–95) of aSN and the PMCA binding site for negatively charged lipids (Fig 4E).

Figure 4. The aSN-PMCA interaction involves the N-terminal region of aSN and the acidic phospholipid binding site of PMCA mediated by acidic phospholipids.

A Schemes of aSN and its N- and C-terminal truncation variants. Bars represent facultatively (lipid-mediated) helical regions, lines—constitutively disordered regions.

B Membrane anchoring of aSN is important for the activation of PMCA. PMCA1d fold activation by titrated full-length aSN and truncated variants. The experiment was performed in presence of 1.8 μM free Ca^{2+} and brain lipid extract was used for the PMCA relipidation. Measurements were performed in four or more replicates. Purified PMCA1d originated from at least two independent expression cultures. Data are expressed as mean ± SEM.

C Sequence comparison of full-length PMCA2w/a and its deletion variants. The PMCA2w/a deletion variants are missing large fragments of the A-TM3 loop: Δ292–383, Δ298–383 Δ298–372. The blue background indicates the acidic phospholipid-binding site.

D The PMCA's acidic phospholipid binding site preceding the TM3 is crucial for the activation by the aSN. *Top*—The specific activity of PMCA in the presence of 1.8 μM free Ca^{2+}—aSN titration experiment. PMCA2w/a and variants were relipidated in the BE and the full-length monomeric aSN was used as the activator. Measurements were performed in four replicates. PMCA proteins used in the study were prepared in a manner where transformation, expression culture, and protein purification were performed in parallel. Purification is reported in the Appendix Fig S2D. *Bottom*—baseline-corrected data from the top graph displayed as fold activation of PMCA by aSN.

E Proposed mechanism of the PMCA-aSN interaction. In the presence of acidic membrane phospholipids (*red*), the N-terminal region of aSN anchors it to the plasma membrane *vis-à-vis* acidic lipids, which mediate the interaction with the acidic lipid-binding site of PMCA, leading to activation of PMCA.

 © 2023 The Authors

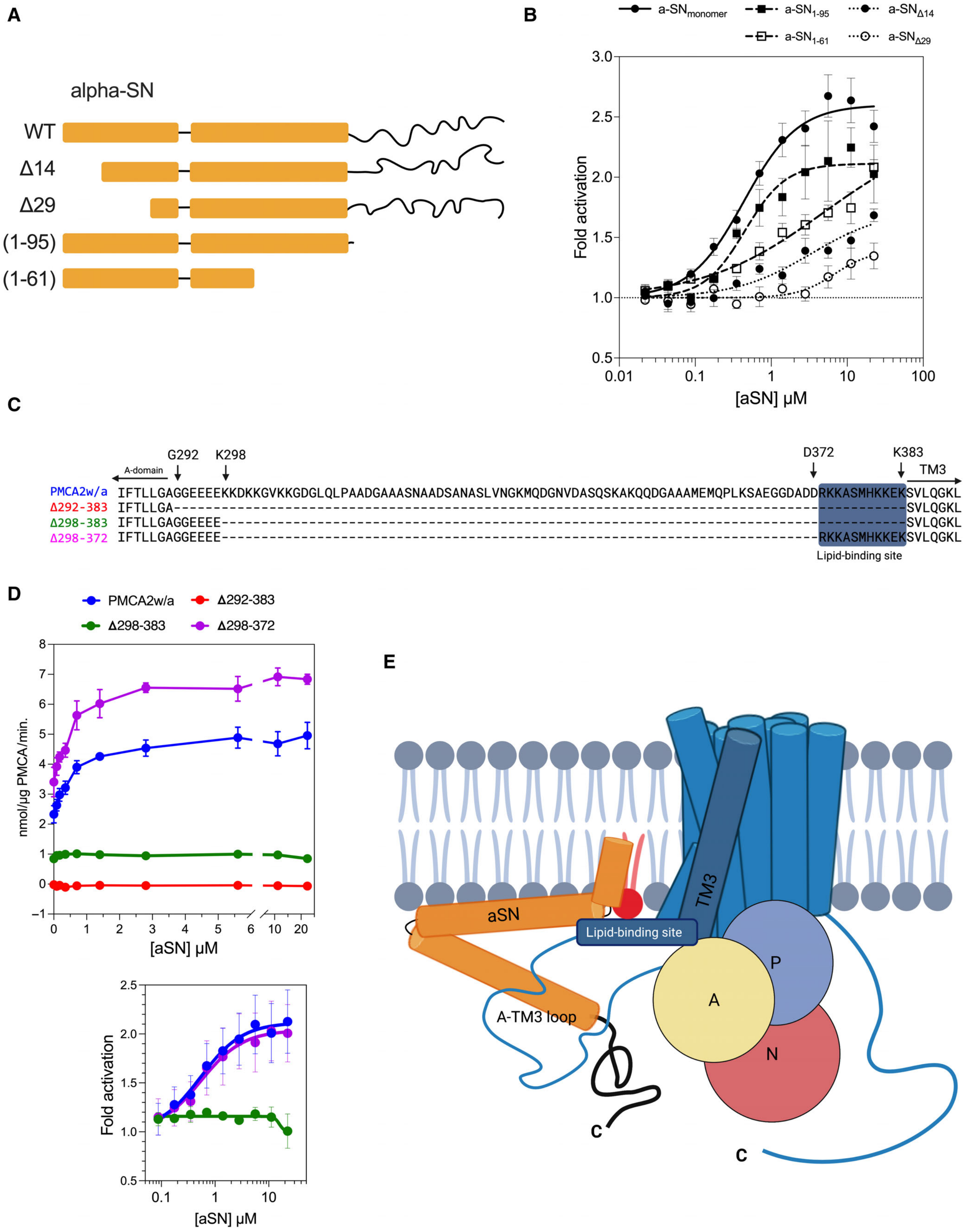
A
alpha-SN
WT
Δ14
Δ29
(1-95)
(1-61)
B
a-SNmonomer
a-SN1-95
a-SNΔ14
a-SN1-61
a-SNΔ29
Fold activation
[aSN] μM
C
A-domain
G292
K298
D372
K383
TM3
PMCA2w/a IFTLLGAGGEEEEKKDKKGVKKGDGLQLPAADGAAASNAADSANASLVNGKMQDGNVDASQSKAKQQDGAAAMEMQPLKSAEGGDADDRKKASMHKKEKSVLQGKL
Δ292-383 IFTLLGA-SVLQGKL
Δ298-383 IFTLLGAGGEEEE-SVLQGKL
Δ298-372 IFTLLGAGGEEEE-RKKASMHKKEKSVLQGKL
Lipid-binding site
D
PMCA2w/a
Δ292-383
Δ298-383
Δ298-372
nmol/μg PMCA/min.
[aSN] μM
Fold activation
[aSN] μM
E
TM3
aSN
Lipid-binding site
A-TM3 loop
P
A
N
C
C

Figure 4.

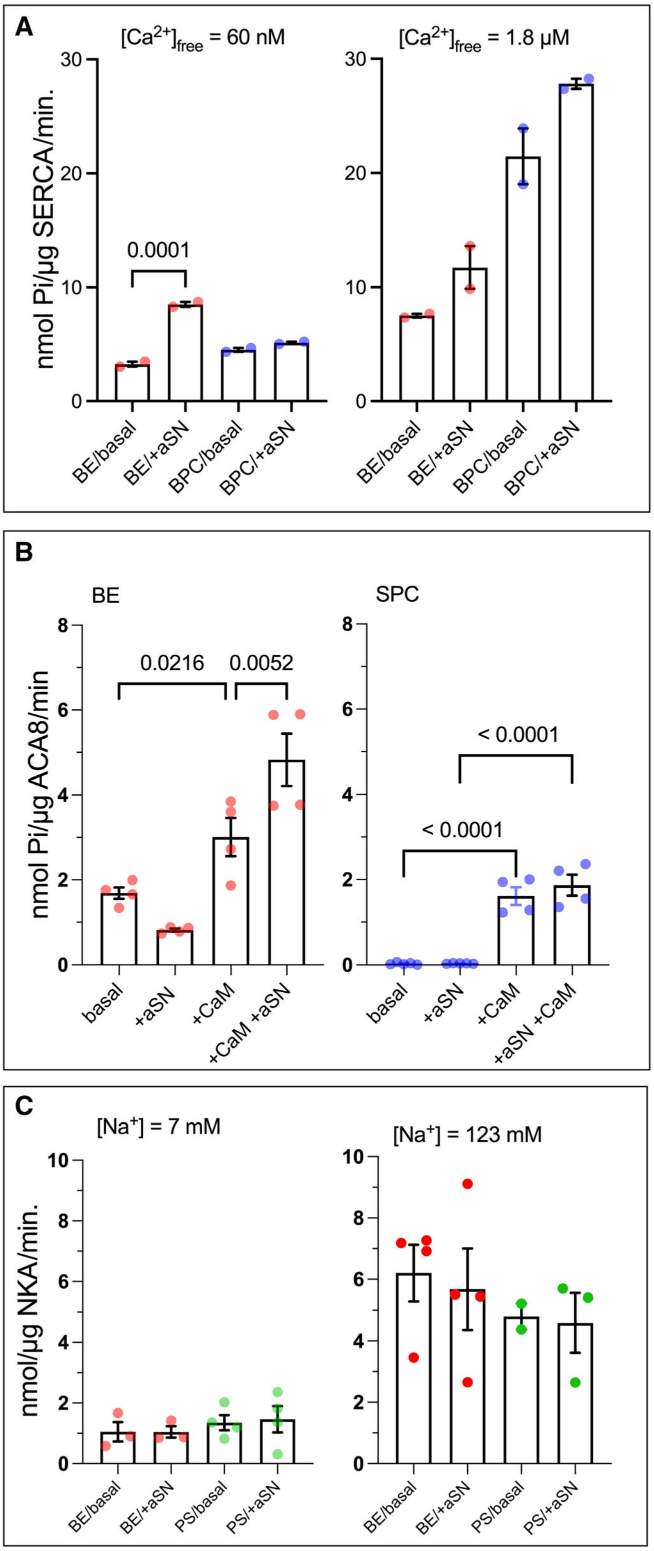

Figure 5. Activation by aSN appears specific to mammalian calcium pumps.

A In presence of acidic lipids, aSN activates rabbit SERCA1a at low $[Ca^{2+}]_{free}$ (60 nM). Bars show SERCA activity without or with aSN when the pump is relipidated in BE (*red dots*) or brain BPC (*blue dots*). $[Ca^{2+}]_{free}$ is 60 nM (*on the left*) and 1.8 μM (*on the right*). Experiments were performed in technical duplicates.

B Plant Ca^{2+} ATPase, ACA8 is not activated by aSN. Bars show the activity of ACA8 in presence of 1.8 μM Ca^{2+} and conditions: without protein partners, with aSN, with CaM, or with both. PMCA was relipidated in BE (*left*, *red dots*) or soy PC (SPC) (*right*, *blue dots*).

C Human Na.K ATPase (NKA) is not activated by aSN. Bars show the activity of NKA in the presence of 7 or 63 nM Na^{+} (right and left graphs, respectively) without or with aSN. NKA was relipidated either in phosphatidylserine (PS, *green dots*) or brain extract (BE, *red dots*). When aSN or CaM are present, the concentrations are 5.6 or 1.2 μM, respectively. No-substrate background values were subtracted. Experiments were performed in technical replicates, where purified proteins originated from single expression culture, data shown as mean ± SEM. Statistical analysis is conducted as multiple comparisons with one-way ANOVA combined with Sidak *post hoc* test.

The activation by aSN is specific to mammalian calcium pump

PMCA activation by aSN is observed both for the ubiquitous PMCA1 and the more tissue-specific PMCA2 and was also verified for the C-terminally truncated PMCA4x isoform (Appendix Fig S3D), which likely colocalizes with aSN in synaptic terminals of retinal neurons (Krizaj *et al*, 2002; Bodis-Wollner *et al*, 2014; Cali *et al*, 2017). The broad impact on PMCA isoforms raised the question whether the stimulatory effect of aSN is at all specific to PMCA.

Interestingly, we found that rabbit SERCA1a also seems to be activated by the aSN monomer in the same lipid-dependent manner as PMCA (Fig 5A). From the two experiments performed, the effect was only predominant in low (60 nM) free Ca^{2+} concentration, whereas at high 1.8 μM free Ca^{2+}, SERCA was activated to a lower fold. This indicates that aSN may also modulate the apparent Ca^{2+} affinity of SERCA. With continuous ER extending into presynaptic compartments (Wu *et al*, 2017; Singh *et al*, 2021), this is also of relevance to aSN function, and most likely it is also related to the previously reported activation by aSN oligomers (Betzer *et al*, 2018).

Next, we turned to a plant homolog of PMCA, namely the autoinhibited calcium ATPase 8 of *Arabidopsis thaliana* (ACA8), belonging to the same P2B subfamily of P-type ATPase (Axelsen & Palmgren, 2001), but not coexisting with aSN, which is only found in vertebrates. Structurally, ACA8 and PMCA differ in the localization of the autoinhibitory domain, which is situated on the N-terminus of ACA8 and the C-terminus in PMCAs, and ACA8 is lacking the long, disordered loop for the A-TM3 loop with the putative lipid-binding site in PMCA (Brodin *et al*, 1992; Pinto Fde & Adamo, 2002; Brini *et al*, 2010). In the presence of BE, ACA8 is not activated by aSN, but rather inhibited, and the effect is reversed by CaM. This may suggest an interplay between aSN, CaM, and acidic lipids, that results in a different effect, albeit weak, for the plant pump (Fig 5B). In the presence of soy PC, the pump is autoinhibited and can be activated by CaM, but not aSN. Furthermore, the human Na,K-ATPase (α1 isoform) was not activated by aSN (Fig 5C). These results suggest the specificity of the aSN interaction for mammalian Ca^{2+}-ATPases.

A mathematical model describes the effect of aSN activation of PMCA on Ca^{2+} levels

To conceptualize the findings on lipid-mediated aSN binding to PMCA and to assess the influence on the calcium concentration in

© 2023 The Authors

the presynaptic terminal cytosol, we developed a mathematical model for the presynaptic calcium ion regulation. It was based on a quantitative model for presynaptic calcium regulation, which integrates the classical Hodgkin-Huxley model for action potential propagation in order to simulate calcium dynamics in response to membrane potential changes (Hodgkin & Huxley, 1952; Erler *et al*, 2004; Keener & Sneyd, 2009). The model presented by Erler *et al* considers Ca^{2+} fluxes through PMCA (ATP-driven Ca^{2+} pump), the sodium-calcium exchanger (NCX), voltage-gated calcium channels (VGCC), and passive fluxes through unknown transport pathways and leakage across the membrane (Fig 6A). We adapted the model such that the PMCA-mediated Ca^{2+} flux depends on the cytosolic aSN concentration, by fitting a kinetic equation for non-essential activation as described in the Supplements (Appendix Fig S8, Equation S1) (Baici, 2015).

Using this model, we investigated the effect of different levels of aSN on the steady state Ca^{2+} concentration (Fig 6B) and the increase in cytosolic calcium in response to one or several action potentials (Fig 6C and D). The simulations showed an increase in the steady-state calcium concentration from 0.1 μM to around 0.25 μM, with the increase being most prominent at aSN concentrations below 10 μM. During an action potential, not only the height but also the duration of the calcium wave were increased. The predominant increase of the calcium wave duration (or width) was caused by an increase of the calcium clearance time (post-peak width, Fig 6D).

To investigate the influence of aSN on the calcium concentration during an action potential spike train, we analyzed the mean calcium concentration during this process. In general, these simulations resulted in an oscillating increase of the calcium concentrations as observed *in vitro* (Chamberland *et al*, 2020) (Fig 6C). Figure 6D (lower right panel) shows the maximum of the mean calcium concentration during an action potential spike train (as visualized in Fig 6C). In response to decreased aSN concentrations, the average calcium concentration increased because of the larger calcium waves at aSN concentrations below 10 μM (Fig 6D). Hence, the presence of monomeric aSN not only stabilizes the steady-state concentration of Ca^{2+}, but also affects the calcium dynamics in response to one or several action potentials.

Discussion

Here we show a strong, activating effect of soluble, monomeric aSN on PMCAs and to a lower degree also SERCA, which conversely was found earlier to be activated by oligomeric aSN (Betzer *et al*, 2018). Our results suggest that a physiological role of aSN is PMCA stimulation for calcium clearance by increasing V_{max} and/or apparent calcium affinity. Due to the very specific localization of aSN, this mechanism would be restricted to specialized cells or cellular compartments like presynaptic termini of neurons. This is supported by our proximity ligation study showing an intracellular colocalization of PMCA and aSN in nerve terminals, and by experiments in primary neurons, where we show that aSN increases calcium expulsion after depolarization. Furthermore, the aSN-stimulated PMCA activity can respond instantly to calcium influx, unlike the calcium-dependent mechanism of CaM activation that requires several binding steps before going into effect.

The charge of phospholipids in the PMCA environment has a great influence on its regulation, and acidic lipids alone can activate PMCA to some extent via specific interaction sites (Carafoli, 1994; Lopreiato *et al*, 2014; Strehler, 2015). In a similar way to the inhibition by tau protein (Berrocal *et al*, 2017), we found that the effect of aSN on PMCA is enabled by negatively charged and not neutral phospholipids in the membrane. The opposite lipid dependence is observed for CaM, consistent with earlier findings (Niggli *et al*, 1981a,b; Lopreiato *et al*, 2014). The composition of the local lipid environment could potentially switch PMCA modes from being responsive either to aSN or CaM.

With its strong effect and accumulation at presynaptic compartments, aSN would allow for a rapid, stimulated response to incoming calcium signals and integration with other calcium-sensitive presynaptic functions such as neurotransmitter release. This, together with aSN activating PMCA independently of the autoinhibitory PMCA C-terminal tail, suggests aSN complements CaM as the activator of PMCA in this specific neuronal compartment.

Alternative splicing of PMCA generates many variants among isoforms and has been for a long time proposed to be a mechanism of fine-tuning calcium handling in cells affecting expression, localization, and membrane trafficking to specific compartments (Strehler & Zacharias, 2001; Antalffy *et al*, 2011; Krebs, 2015). Splice site "A" variants of PMCA (w, x, y, z) differ in the size of the lengthy and disordered A-TM3 loop (Fig 3C). That region was not resolved in a cryo-EM structure of PMCA1 (Gong *et al*, 2018), and its impact on the enzymatic properties is not clear, but the proximity to the membrane interface and cytosolic Ca^{2+} entry suggests a modulatory role of the A-TM3 loop, and its involvement in PMCA-lipid interaction and membrane trafficking has been proposed (Adamo & Penniston, 1992; Antalffy *et al*, 2011; Enyedi & Strehler, 2011). Indeed, our mutational studies indicate a modulation of the Ca^{2+} transport activity. In PMCA, the deletion of the intrinsically disordered A-TM3 loop (deleting residues 298–383 in PMCA2w/a) retained basal enzymatic function, but the protein was unresponsive to aSN. However, the stimulation was preserved in a Δ298–372 variant, where 11 residues upstream of the TM3 are maintained. This fragment, highly specific in sequence to PMCA, is rich in positive charge and was previously shown to interact with acidic lipids (Adamo & Penniston, 1992; Pinto Fde & Adamo, 2002), further discussed below. Additionally, this variant showed increased basal activity, which may indicate a role of the A-TM3 loop in some level of autoinhibition that complements the autoinhibitory domain at the C-terminal end of PMCA.

The correlations found between aSN expression level and splicing events at the "C" site of PMCA isoforms 1 and 4 suggest that an "aSN/CaM balance" of PMCA activation can be managed by alternative splicing, leading to the synthesis of less CaM-sensitive variants in tissues destined for aSN activity. Variations obtained at the splice site "A" of PMCA2 suggest preference for variant "w" in low-aSN-expressing tissues and shorter variants "x" and "z" being expressed together with increasing aSN level. In the activity assay, the PMCA2 splice variants responded differently to aSN; PMCA2w/a was the only ones having their apparent calcium affinity increased by aSN, but was conversely stimulated to the lowest maximum activity and had relatively low apparent calcium affinity in a basal (non-activated) state. The longest A-TM3 loop of PMCA2w (Fig 2D) can be associated with the furthest flexibility in the regulation of both affinity and rate. For PMCA2x and -z, only the rate was stimulated.

© 2023 The Authors

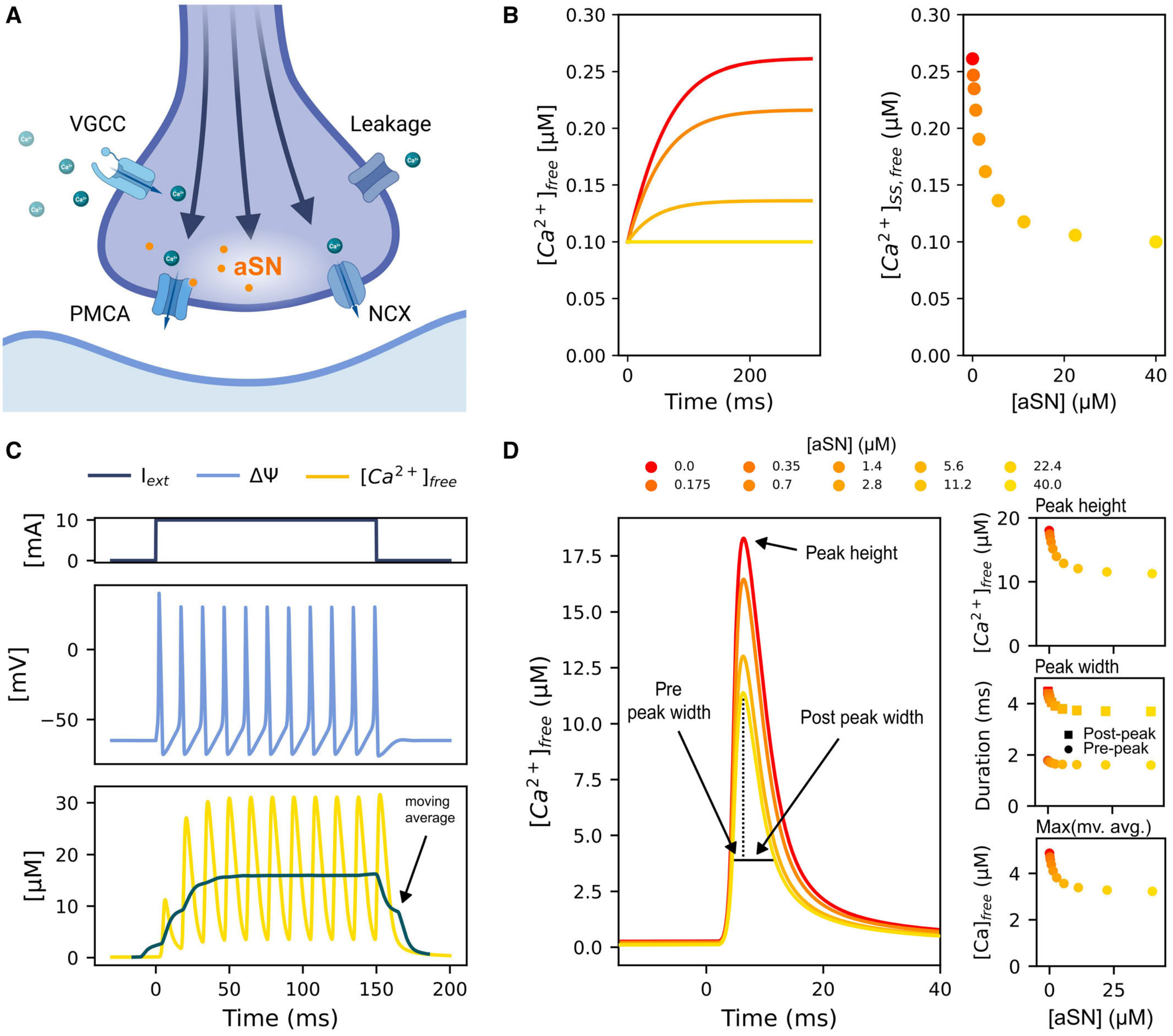

Figure 6. Mathematical model of calcium dynamics in the presynaptic terminal predicts increased calcium accumulation in response to decreased aSN concentration.

A Schematic of a presynaptic terminal. The schematic displays the relevant transport mechanisms of calcium regulation, which were integrated in the mathematical model. Those include voltage-gated calcium channels (VGCC), the aSN-activated Ca^{2+}-ATPase PMCA, NCX, and calcium leakage channels.

B Decreased aSN concentration results in increased calcium concentration. Decreasing the aSN-concentration in the presynaptic terminal results in an increase in the calcium concentration over time (left panel), leading to a new (aSN-dependent) steady state (right panel).

C Oscillatory behavior of the calcium concentration in the presynaptic terminal. The model is able to generate action potential spike trains, during which the calcium concentration in the presynaptic terminal displays oscillatory behavior around an increasing mean concentration (moving average).

D The reduction of aSN leads to an increase in the height and the width of a single action potential. The subfigure shows the effect of the aSN concentration on system features such as single peak height, peak width, and the maximum of the moving average concentration during a spike train (as defined in C). *Main panel*: The action potential generated at different aSN concentrations. A reduction in aSN leads to an increase in the height and the width. Peak height, pre-peak width, and post-peak width are indicated by arrows. *Upper right panel*: Action potential peak height at different aSN concentrations. The peak height describes the maximum calcium ion concentration reached in response to a single action potential. *Middle right panel*: Changes in pre-peak width and post-peak width. The peak width measures the duration of the calcium ion concentration transient. *Lower right panel*: The maximum of the moving average (mv. avg.) concentration during an action potential spike train (20 Hz) increases as a consequence of the increase in height and width of a single action potential in response to reduced aSN concentrations. The moving average of the calcium concentration is found by averaging over the calcium concentration in response to a 20 Hz action potential spike train with 400 ms duration.

 © 2023 The Authors

These two variants are typically expressed in aSN-abundant tissues (Fig 3), where higher capacity for calcium extrusion is required, such as in neurons.

The specific function of soluble, monomeric aSN is debated (Cheng *et al*, 2011; Sulzer & Edwards, 2019), but preferential association to membranes with acidic phospholipids is a recurring theme (Davidson *et al*, 1998; Jo *et al*, 2000). The ability to activate PMCA appears strongly related to its lipid-binding properties. The aSN A30P mutant form, associated with familial early-onset PD, is known for reduced ability to bind acidic phospholipids (Jo *et al*, 2002), and we find here that it correlates with weak PMCA activation. Similar dependence emerges from the characteristics of the N-terminus of aSN. Monomeric aSN interacts with acidic phospholipids via the ~ 95 residues long N-terminal part adopting amphipathic helical segments (Ulmer *et al*, 2005; Uversky & Eliezer, 2009). The aSN binding to lipid bilayers has been proposed to be maintained by avidity between the 1 and 14 region, strongly interacting and anchoring into the lipid bilayer, and the downstream amphipathic helix having weak surface binding capacity (Cholak *et al*, 2020). Indeed, here we observed a gradual loss of the PMCA activating properties by deletion of 1–14 or 1–29 residues, with the Δ(1–29) construct being almost incapable of activating PMCA (Fig 4B). Moreover, the importance of the N-terminus is also indicated by the observation that both beta- and gamma-synuclein can activate PMCA, as this is a highly conserved region among synuclein isoforms (Hayashi & Carver, 2022).

From a structural and mechanistic point of view, we propose that PMCA is activated by a dynamic *vis-à-vis* interaction between the N-terminus of the monomeric aSN and the PMCA binding site for negatively charged lipids, located at the C-terminal side of the disordered A-TM3 loop, and mediated by acidic lipids (Fig 4E). The bulk part of the loop can play a tuning role together with the disordered C-terminal tail of aSN. With medium-affinity Ca^{2+}-binding properties (K_d ~ 20–50 μM) (Lautenschlager *et al*, 2018; Newcombe *et al*, 2021), this region of aSN could potentially increase the local concentration of Ca^{2+} at the cytosolic entry site of PMCA. It appears to play a role in stimulation of the PMCA rate but does not have a visible impact on apparent calcium affinity.

The lipid-binding site of PMCA is likely functionalized by the many positively charged residues in the 11-residue sequence defining it (RKKASMHKKEK, see also Appendix Fig S5). The equivalent sequence in the plant ortholog ACA8 (MASISEDNGEE, residue 352–362) is negatively charged, as is the N-terminal part of the A-TM3 loop in the Δ298–383 deletion construct of PMCA2 (TLLGAGGEEEE). Both ACA8 and PMCA2-Δ298–383 show significant basal activity in presence of acidic lipids but are not activated by aSN. The corresponding region in SERCA (RDQMAATEQDK, residues 236–246 of rabbit SERCA1a) shows a mixed positive and negative charge, which may explain a mild activation by aSN and negatively charged lipids. We note that aSN contains several K-rich motifs in its N-terminal region (1–61) that have resemblance to the acidic-lipid binding sequence of the disordered loop of PMCA; an observation that together with the absence of a direct interaction in simple systems (Fig EV2) and a neurotoxic effect of their mutation (Dettmer *et al*, 2015b) strengthens the suggestion of a lipid-mediated interaction as the mechanism of aSN activation of PMCA in neurons.

The function of aSN oligomers and fibrils has been thoroughly studied, and among a plethora of cytotoxic capacities, they have been linked to calcium dysregulation by over-activation of SERCA (Danzer *et al*, 2007; Rcom-H'cheo-Gauthier *et al*, 2016; Betzer *et al*, 2018). We show here that native, monomeric aSN exerts a strongly activating effect on PMCA, whereas oligomeric aSN only activates weakly, which perhaps can be ascribed to the small amount of released monomer (Appendix Fig S4).

The mathematical modeling of presynaptic calcium levels shows aSN activation of PMCA affects both the steady-state concentration of Ca^{2+} and the calcium dynamics (Fig 6). At low levels of aSN in the presynapse, the basal Ca^{2+} concentration is increased and Ca^{2+} homeostasis is disrupted during sustained neuronal activity. The accumulation of Ca^{2+} only happens at monomeric aSN concentration below 10 μM ensuring that small fluctuations in aSN concentrations in the presynapse do not impact calcium regulation.

This novel function of aSN is relevant to calcium homeostasis of neurons and specifically presynaptic compartments, where PMCA plays a key role in calcium homeostasis; however, potentially it affects also the extracellular environment, where the exchange of each calcium ion for two protons by PMCA leads to transient alkalinization of nanodomains at the synaptic cleft with consequences to postsynaptic NMDA receptor fluxes (Chen & Chesler, 2015; Feghhi *et al*, 2021). Furthermore, the activated proton import of PMCA may be important for neutralization of an acidified environment at the synaptic cleft with large release activity of acidified neurotransmitter vesicles.

The role of aSN in hematopoietic system has been discussed (Pei & Maitta, 2019). The interaction with PMCA can be potentially important to calcium homeostasis in erythrocytes, where aSN is also very abundant (Barbour *et al*, 2008). Erythrocytes lack ER and mitochondria, and their calcium homeostasis depends solely on PMCA, so aSN could contribute to the very efficient calcium clearance mechanism of erythrocytes, which maintains resting calcium on a very low, 30–60 nM level (Bogdanova *et al*, 2013).

In the presynapse, PMCA-dependent control of the local calcium-depletion zones could be enforced by aSN and acidic lipids. This may for instance have importance for synaptic vesicle release and recycling where PMCA was proposed to functionally separate simultaneous calcium signals (Krick *et al*, 2021). Oligomerization and aggregation/fibrillation of aSN on the other hand would impair this function and lead to impaired calcium handling. Our finding can then also have implications regarding aSN pathology. Lipid-related dysfunctions have been linked to PD (Fanning *et al*, 2020). Early manifestations of PD could result from calcium dyshomeostasis caused by aggregating aSN having loss of function on PMCA activation, or by lipid-affinity-affecting mutations like A30P, which indeed leads to early neuronal dysfunctions (Kruger *et al*, 2001), or changes in the distribution and content of acidic plasma membrane lipids. Furthermore, impaired control of extracellular pH can be affected and cause neurotoxic effects. These findings merit a focus on the coupled function of PMCA, SERCA, aSN, and calcium homeostasis in neurodegenerative disorders.

Materials and Methods

Co-immunoprecipitation assay

Brains from C57BL/6 mice (Janvier Labs) or aSN-KO (Sncatm1Rosl [C57BL/6, The Jackson Laboratory]) were homogenized in 7× w/v

© 2023 The Authors

homogenization buffer (320 mM sucrose, 4 mM HEPES–NaOH, 2 mM EDTA, and complete protease inhibitor mix [Roche], pH 7.4) using a loose-fitting glass-Teflon homogenizer (10 up-and-down strokes, 700 rpm). Debris was removed from the homogenate by centrifugation for 10 min at 1,000 *g* in a Sorvall RC 5C plus centrifuge. The resulting supernatant was centrifuged for 1 h at 100,000 *g*. The supernatant was removed, and the pellet was resuspended in the original volume (7×) of RIPA (50 mM Tris pH 7.4, 159 mM NaCl, 1% Triton X-100, 2 mM EDTA, 0.5% sodium deoxycholate, 0.1% SDS) for 3 h, whereafter samples were spun at 20,000 *g*, for 30 min at 4°C. Protein concentration was measured using the bicinchoninic acid assay. aSN oligomer or monomer (2 μg/ml) was mixed with 0.5 mg/ml aSN-KO mouse brain homogenate diluted in PBS, 0.5% Triton X-100 and incubated overnight at 4°C.

aSN binding (ASY-1) or control (non-immune IgG) was performed as previously described (Betzer *et al*, 2015). The samples were incubated for 2 h with rotation. The Sepharose beads were isolated and washed twice with PBS, 0.5% Triton X-100, and Co-IP proteins were eluted by incubation in a non-reducing SDS loading buffer at room temperature. Proteins were resolved on 10–16% gradient SDS–PAGE under reducing conditions followed by immunoblotting for PMCA (primary antibody: 5F10 anti-pan-PMCA, Abcam, ab2825, secondary antibody: anti-mouse-HRP, Dako) and aSN (primary antibody: anti-Syn-1, BD Transduction Laboratory, 610787 secondary antibody: anti-mouse-HRP, Dako, P0260). The interaction between endogenous aSN and the endogenous PMCA was studied in the extracts from C57BL/6 mice as described above.

Primary hippocampal neuronal cultures and cytosolic Ca^{2+} measurements

Primary hippocampal neurons were cultured from newborn (P0) aSN-KO mice (Sncatm1Rosl [C57BL/6], The Jackson Laboratory). Hippocampi were dissected in ice-cold Hank's balanced salt solution, dissociated in 20 U/ml papain in Hibernate A medium (Gibco) supplemented with 1xB27 and 0.3 g/l L-glutamine for 20 min at 37°C, washed twice, and triturated in plating medium (MEM [Gibco] supplemented with 5 g/l glucose, 0.2 g/l $NaHCO_3$, 0.1 g/l transferrin, 0.25 g/l insulin, 0.3 g/l L-glutamine, and 10% fetal bovine calf serum [heat-inactivated]). Hippocampal neurons were seeded on Matrigel® matrix (Corning®)-coated coverslips. After 24 h, the medium was changed to growth medium (MEM supplemented with 5 g/l glucose, 0.2 g/l $NaHCO_3$, 0.1 g/l Transferrin, 0.075 g/l L-glutamine, 1× B-27 supplement, 2 μM cytosine arabinoside and 5% fetal bovine calf serum [heat-inactivated]).

At culture day 6 (DIV 6), the neurons were transiently transfected by lipofectamine 3000 with Bicistronic vectors coding for mCherry and aSN or mCherry under synapsin promotor according to manufacturer's instructions yielding varying expression levels in individual neurons. At culture day 8, cytosolic Ca^{2+} levels in primary neurons were determined using the Ca^{2+}-sensitive fluorescent indicator Fura-2-AM (Molecular Probes/Invitrogen). Cells were loaded with Fura-2 in sterile-filtered HEPES-buffered saline (HBS: 20 mM HEPES, 150 mM NaCl, 5 mM KCl, 1 mM $CaCl_2$, 1 mM $MgCl_2$, 10 mM glucose, pH 7.4) containing 2.5 μM Fura-2 AM, 0.04% pluronic acid, F127 for 30 min at 37°C, 5% CO_2. The Fura-2-containing medium was replaced with fresh HBS without Fura-2 and incubated additionally for 30 min. After dye loading and prior microscopic analysis, the culture was moved into a recording buffer with thapsigargin to inhibit contribution from SERCA for 5 min., and an area containing 1–3 transfected neurons was found. The fluorescence was measured on an Olympus Scan^R high-content microscope using excitation wavelengths at 340 and 380 nm and emission at 510 nm. The cytosolic Ca^{2+} levels in single cells were measured by placing a region of interest (ROI) outside the nucleus. The recording was started and at the recording time of 2 min KCl was added to depolarize the neurons. The calcium response was followed over time until 12 min. The response upon K^+-induced depolarization was quantified as the area under curve (AUC) from each measured neuron from the 2–12 min. measurement interval.

Cytosolic Ca^{2+} measurements in SH-SY5Y cells

A doxycycline controlled SH-SY5Y human cell line that stably overexpresses wt human aSN (SH-SY5Y ASYN) was used (Vekrellis *et al*, 2009). In the presence of doxycycline, aSN expression is suppressed. SH-SY5Y ASYN cells were cultured in the presence of 1 μM doxycycline similarly to previously described (Reimer *et al*, 2022).

SH-SY5Y ASYN cells were split and incubated with or without 1 μM doxycycline for 3 days, then split and seeded onto polylysine-coated coverslips still with or without 1 μM doxycycline and cultured for an additional 2 days, whereafter calcium extrusion experiments were performed using the fluorescent calcium binding dye Fluo-8 AM (ATT Bioquest).

The cells on the coverslips were loaded with 2.5 μM Fluo-8 in loading buffer (140 mM NaCl, 11.5 mM glucose, 5.9 mM KCl, 1.8 mM $CaCl_2$, 1.4 mM $MgCl_2$, 1.2 mM NaH2PO4, 5 mM $NaHCO_3$, 10 mM Hepes pH 7.4, 2.5 μM Fluo-8 AM) for 30–60 min. at 37°C, 5% CO_2. Following Fluo-8 loading, coverslips were washed once in experiment buffer (140 mM NaCl, 11.5 mM glucose, 5.9 mM KCl, 1.8 mM $CaCl_2$, 1.4 mM $MgCl_2$, 1.2 mM NaH_2PO_4, 5 mM $NaHCO_3$, 10 mM Hepes pH 7.4).

Coverslips were then mounted in an imaging chamber and covered with 300 μl pre-heated (37°C) recording buffer (140 mM NaCl, 11.5 mM glucose, 5.9 mM KCl, 1.8 mM $CaCl_2$, 1.4 mM $MgCl_2$, 1.2 mM NaH_2PO_4, 5 mM $NaHCO_3$, 10 mM Hepes pH 7.4, 4 μM thapsigargin) and placed in a 37°C pre-heated OkoLab imaging chamber on the microscope stage.

Imaging was performed on a Nikon T*i* Eclipse inverted microscope equipped with an OkoLab heating chamber, Perfect Focus 3 system, a Plan Apo 60× (NA 1.40) oil objective, a Zyla sCMOS5.5 Megapixel camera (Andor), fluorescence illumination system CoolLED-pE-300^{white}, and the fluorescence filter set for GFP. The system was controlled by NIS Elements software from Nikon. Imaging was performed similar to previously described (Ernstsen *et al*, 2022). Images were acquired every second for 8 min in total. Binning of 2 was used. Following baseline imaging for 30 s, 600 μl depolarization buffer (140 mM NaCl, 11.5 mM glucose, 132 mM mM KCl, 5.7 mM $CaCl_2$, 1.4 mM $MgCl_2$, 1.2 mM NaH_2PO_4, 5 mM $NaHCO_3$, 10 mM Hepes pH 7.4, 4 μM thapsigargin) was added into the center of the imaging chamber, while imaging continued. Two technical replicates were performed. A square region of interest was placed in the cytoplasm of cells (2–4 cells per replicate)

 © 2023 The Authors

for all frames in the time-lapse sequences (481 frames) of the different conditions and mean fluorescence intensity was measured using ImageJ (Schindelin *et al*, 2012). The response to K+ induced depolarization was quantified as the Area Under Curve (AUC) for each cell.

Proximity ligation assay (PLA)

Proximity ligation assay (PLA) enables *in situ* detection of protein–protein interaction using single-stranded oligonucleotides conjugated to specie-specific antibodies. If the oligonucleotides are in close proximity (within 40 nm), they can be ligated to form circular DNA, which can be amplified by multiplying the binding site for fluorescently labeled complementary oligonucleotides and thus multiplying the fluorescent readout.

Primary hippocampal neurons were cultured from newborn (P0) C57BL/6 mice (Janvier Labs) as described for aSN-KO. At culture day 14 (DIV14), the neurons were fixed in 4% paraformaldehyde for 30 min at room temperature (RT) followed by a wash in PBS and 10 min permeabilization in 0.1% Triton X-100, 50 mM glycine, 3 mM $CaCl_2$, 2 mM $MgCl_2$, pH 7.4. Unspecific binding was blocked by 3% bovine serum albumin in PBS for 1 h followed by incubation with the primary antibody for 1.5 h [anti-AS (1 μg/ml, ASY-1, Lindersson *et al*, 2004; Kragh *et al*, 2009), and anti-PMCA 5F10 (1 μg/ml, Abcam, ab2825)]. The Duolink procedure was conducted according to the manufacturer's instructions with the Duolink® *In Situ* Red Starter Kit Mouse/Rabbit (Duolink®, Sigma-Aldrich), with secondary antibodies contained in the kit. After PLA staining, the neurons were labeled with synaptophysin (primary—antibody—guinea pig anti-synaptophysin 1, (Synaptic Systems #101004), secondary: goat anti-guinea pig, Alexa Fluor® 488 nm (Abcam, ab150185) to visualize the synapses where synuclein normally is located and DAPI for nuclei. Images were obtained using a Zeiss Observer Z1 inverted microscope equipped with ApoTome.2.

Expression and purification of α-synuclein

Recombinant human aSN wild type and variants were produced in *Escherichia coli*, and purified as previously described (Huang *et al*, 2005). Monomeric and oligomeric forms of aSN were produced and isolated as previously described (Betzer *et al*, 2015). Concentration of all aSN preparations was confirmed by BCA assay. The proteins were stored in −80°C in a buffer containing 20 mM Tris–HCl, pH 7.5, 150 mM KCl. Recombinant ^{15}N-aSN for NMR experiments and aSN(Δ1-14) was prepared as previously described (Cholak *et al*, 2020) and aSN(1–61) as in (Skaanning *et al*, 2020). Prior to the experiments, proteins were subjected to size exclusion chromatography on Superdex 200 increase 10/300 column (GE Healthcare) in the buffer containing 50 mM Tris–HCl pH 7.2, 150 mM KCl. Fractions were pooled, concentrated to 1–1.5 mg/ml 5 kDa MWCO Vivaspin protein concentrator (Sartorius), flash-frozen in liquid nitrogen, and stored in −80°C for further experiments.

Size exclusion chromatography profiles and SDS–PAGE analysis are presented in Appendix Fig S2.

PMCA expression constructs and site-directed mutagenesis

Codon-optimized genes encoding for human PMCA1d and PMCA2w/a (Genscript) were cloned into pEMBL-yex4 expression plasmids by homologous recombination in *S. cerevisiae* as described (Drew *et al*, 2008).

PMCA1d and PMCA2w/a constructs were subjected to site-directed mutagenesis using QuikChange site-directed mutagenesis kit according to the manufacturer's protocol (Agilent). To obtain an inactive variant D475N (the autophosphorylation site), mutagenetic primers were designed using PrimerX online tool (https://www.bioinformatics.org/primerx). To obtain C-terminally truncated PMCA1, an internal TEV cleavage site was introduced to the original construct. Primers for the insertion of codon-optimized TEV site sequence and the large deletions in PMCA2 were designed according to described methods (Liu & Naismith, 2008). The integrity of all constructs was verified by DNA sequencing (Eurofins).

Expression of recombinant PMCAs in *S. cerevisiae*

All circular vectors were introduced into yeast cells using PEG/LiAc/ssDNA-mediated transformation (Gietz & Schiestl, 2007). All recombinant PMCAs were expressed in *S. cerevisiae* strain K616, depleted of the native calcium ATPases (*MATα pmr1::HIS3 pmc1::TRP1 cnb::LEU2 ura3-1*) (Cunningham & Fink, 1994). All culture media were supplemented with 10 mM $CaCl_2$, as it is a necessary survival condition for the strain to maintain internal stores. For expression culture, 100 ml of uracil-deficient 2% glucose SD medium was inoculated with a colony of transformed cells and allowed to grow for 24 h in 30°C in a 120 rpm shaking incubator. The preculture was used for inoculation of 9 L of -Ura SD medium with 1% glucose and 40 mg/L adenine hemisulfate, starting from OD_{450} of 0.05. After 22 h, when all glucose in the medium was used and the culture reached OD_{450} of ~ 5, the expression was induced by 2% galactose, and the culture was supplemented with YP medium and 40 mg/L adenine hemisulfate. Cells were harvested after 18–20 h by centrifugation, washed with water and TEKS buffer (50 mM Tris–HCl pH 7.6, 2 mM EDTA, 100 mM KCl, 0.6 M D-sorbitol), and resuspended in TESin buffer (50 mM Tris–HCl, 2 mM EDTA, 0.6 M D-sorbitol with the addition of PMSF and protease inhibitor. The typical yield was ~ 15 g of cells per liter of culture.

Purification of PMCAs

The cells were disrupted in a pulverisette grinder with an equal volume of 0.5 mm glass beads (4°C, 450 rpm, 4 cycles of 3 min millings, and 1 min pause). The material was centrifuged for 20 min at 2,000 *g* to remove beads and uncracked cells. The supernatant (S1) was centrifuged for 30 min at 20,000 *g* for further removal of cell debris. The supernatant (S2) was subjected to ultracentrifugation for 3 h at 150,000 *g*. Pelleted membranes were suspended in solubilization buffer (20% glycerol, 50 mM Tris–HCl pH 7.2, 150 mM KCl, BME, PMSF, protease inhibitors) and homogenized in a Dounce glass homogenizer. Membranes were flash-frozen in liquid nitrogen and stored at −80°C until further processing.

Membranes were diluted with solubilization buffer to 5 mg/ml total protein concentration (measured with Bradford assay) and solubilized in 1% DDM and 0.2% cholesteryl hemisuccinate for 1 h by stirring in 4°C, followed by ultracentrifugation at 150,000 *g* for 1 h to pellet unsolubilized material. The supernatant was supplemented with 4 mM $CaCl_2$ and stirred for 2 h in 4°C with CaM-Sepharose beads (GE Healthcare), pre-equilibrated with binding buffer (50 mM

© 2023 The Authors

Tris–HCl pH 7.2, 150 mM KCl, 0.017% w/v DDM, 10% glycerol, 2 mM $CaCl_2$). The beads were packed onto a gravity flow column and washed with 10–15 column volumes of binding buffer. The protein was eluted a buffer containing 50 mM Tris–HCl pH 7.2, 150 mM KCl, 0.017% w/v DDM, 10% glycerol, 2 mM EGTA. EGTA stock solution (200 mM) was beforehand pH-adjusted with KOH to ~ 7.5. The fractions containing the protein of interest were pooled and concentrated to ~ 3 mg/ml using a 100 kDa MWCO Vivaspin protein concentrator (GE Healthcare). The concentrated protein was subjected to size exclusion chromatography on Superose 6 increase 10/300 column (GE Healthcare) in the buffer containing 50 mM Tris–HCl pH 7.2, 150 mM KCl, 0.017% w/v DDM, 10% glycerol. Fractions were pooled, concentrated to 1–1.5 mg/ml, flash-frozen in liquid nitrogen, and stored in −80°C for further experiments. Size exclusion chromatography profiles and SDS–PAGE analysis of all PMCA variants used in the study are presented the Appendix Fig S2.

To produce C-terminally truncated PMCA1, the PMCA1d construct with a tobacco etch virus (TEV) protease cleavage site inserted next to H1074 just before the autoinhibitory domain was used. The protein was cleaved with TEV protease while bound to the CaM sepharose beads. After overnight incubation with the protease at 4°C, the C-terminally truncated PMCA was collected in the flow-through fraction and run over a Ni^{2+} affinity column to bind the His-tagged protease. The flow-through was concentrated to ~ 3 mg/ml (100 kDa MWCO) and subjected to size exclusion chromatography on Superdex 200 increase 10/300 column (GE healthcare) in size exclusion buffer containing 50 mM Tris–HCl pH 7.2, 150 mM KCl, 0.017% w/v DDM, 10% glycerol. Peak fractions of PMCAs were pooled, concentrated to 1–1.5 mg/ml using a 100 kDa MWCO Vivaspin protein concentrator (Sartorius), aliquoted, and flash-frozen in liquid N_2 and stored at −80°C for further procedures. The purity of the proteins was analyzed by SDS–PAGE (Appendix Fig S2).

Pull-down assay

CaM-Sepharose beads (GE Healthcare) were pre-equilibrated with binding buffer (40 mM BisTris/HEPES pH 7.2, 100 mM KCl, 0.017% w/v DDM, 3 mM $MgCl_2$, 2 mM $CaCl_2$). PMCA was preincubated with aSN in the mixture of 80 µl total volume containing 0.6 mg/ml PMCA2w/a, 1.5 mg/ml aSN, and 2.5 mg/ml brain lipid extract (BE). Both PMCA and aSN stock solutions were in their final storage buffers, BE stock solution contained 50 mM Tris–HCl pH 7.2, 150 mM KCl, 1% DDM. Simultaneously, the preincubation mixture without lipids was also prepared. Control samples without PMCA or aSN were prepared. After 30 min. preincubation in room temperature, pre-equilibrated CaM-sepharose beads were added and the mixture was diluted to 1 ml with the binding buffer. For the samples where lipids were present, the binding buffer contained 100 µg/ml brain lipid extract. Samples were incubated with agitation in the room temperature for 60 min and washed four times with respective binding buffer. Each wash cycle consisted of 2 min. centrifugation at 900 *g* to pellet the beads, removal of the supernatant, and addition of 1 ml of the fresh buffer. Finally, 40 µl of the elution buffer (40 mM BisTris/HEPES pH 7.2, 100 mM KCl, 0.017% w/v DDM, 3 mM $MgCl_2$, 2 mM EGTA) was added to the beads and after short centrifugation, supernatant was collected, and analyzed by SDS–PAGE and western blotting. We used primary antibodies: rabbit ASY-R1 (Jensen *et al*, 2000), mouse 5F10 anti-pan-PMCA (Abcam, b2825); secondary antibodies: anti-rabbit-HRP (DAKO–P0217), anti-mouse-HRP (DAKO–P0260).

Expression and purification of CaM

Mammalian CaM in the pET15b vector was transformed into *E. coli* strain C41 and grown in LB media at 37°C. Induction was performed with IPTG at an OD_{600} of 0.6. After 16 h of growth at 21°C, cells were harvested by centrifugation at 3,000 *g*, resuspended in lysis buffer (20 mM Tris–HCl pH 7.5, 1 mM EDTA, complete protease inhibitor), and lysed using sonication. The lysate was centrifuged for 1 h at 20,000 *g*. The supernatant was mixed 1:1 with wash buffer (20 mM Tris–HCl pH 7.5, 10 mM $CaCl_2$) and loaded onto a pre-equilibrated (wash buffer) gravity flow phenyl sepharose column. The column was washed with 5 CV wash buffer, 5 CV high salt wash buffer (20 mM Tris–HCl pH 7.5, 500 mM NaCl, 10 mM $CaCl_2$), and 5 CV wash buffer. CaM was eluted in elution buffer (50 mM Tris–HCl pH 7.5, 150 mM NaCl, 10 mM EGTA). Before use in activity assays, CaM buffer was exchanged to 20 mM Tris–HCl pH 7.5, 150 mM KCl using PD MiniTrap G25 desalting column (GE Healthcare), aliquoted, and stored in −80°C.

Expression and purification of ACA8

ACA8 expression and membrane isolation was performed similarly to the PMCA isoforms. ACA8 was solubilized in a buffer containing 50 mM Tris–HCl pH 7.6, 100 mM KCl, 20% glycerol, 3 mM $MgCl_2$, 5 mM β-mercaptoethanol (βME), 7.5 mg/ml DDM, 1 mM PMSF, and 1 µg/ml of chymostatin, pepstatin A, and leupeptin. Purification was achieved through Ni^{2+} affinity followed by size exclusion chromatography. ACA8 was bound to a Ni^{2+}-affinity column in low imidazole buffer (50 mM Tris–HCl pH 7.5, 10 mM imidazole, 200 mM KCl, 10% glycerol, 3 mM $MgCl_2$, 5 mM βME, 0.2 mM LMNG). On the column, the ACA8 His-tag was cleaved off with thrombin and ACA8 was eluted in low imidazole buffer. ACA8 was subjected to size exclusion chromatography on a Superose 6 increase 10/300 column in a SEC buffer (50 mM Tris–HCl pH 7.5, 200 mM KCl, 3 mM $MgCl_2$, 10% glycerol, 0.02 mM LMNG, 5 mM βME). The protein was stored in −80°C in SEC buffer for further experiments.

Expression and purification of PMCA2wA-TM3 loop

Escherichia coli cells (BL21 DE3) were transformed with a modified pET24b plasmid containing N-terminal His_6-SUMO tagged $PMCA2w_{A\text{-}TM3}$ loop (L289-K383) using heat shock transformation. Pre-cultures (10 ml LB medium, 50 µg/ml kanamycin) were grown overnight and used to inoculate 1 L of ZYM-5052 media (Studier, 2005), supplemented with 50 µg/ml kanamycin. Cells were grown for 3 h at 37°C with shaking, then transferred to a shaking incubator at 16°C, and grown for a further 24 h. The cells were pelleted at 4,000 *g* and kept at −20°C for at least 24 h prior to lysis in column binding buffer (50 mM Tris pH8, 150 mM NaCl, 10 mM Imidazole), supplemented with complete protease inhibitor (Sigma), at 20 kpsi using a French pressure cell disruptor (Constant Systems, Daventry, UK). Lysate was cleared by centrifugation at 20,000 *g*, then passed through a 0.45 µm filter before being incubated with

© 2023 The Authors

equilibrated Ni-Sepharose fast flow resin (GE Healthcare) for 30 min. Lysate was passed through the column via gravity flow, followed by wash buffer (5× CV) (50 mM Tris pH8, 1 M NaCl, 10 mM Imidazole) 5× CV binding buffer, and eluted with elution buffer (10 ml; 50 mM Tris pH 8, 150 mM NaCl, 250 mM Imidazole). Purified His_6-SUMO-PMCA2w was made up to 50 ml (with 50 mM Tris pH 8, 150 mM NaCl) and cleaved with ULP1 (0.1 mg) ON at 4°C. The cleaved protein was purified by collecting the flow-through from a second Ni-Sepharose column and concentrated using Amicon spin filters (Millipore). Protein was stored at −20°C.

NMR experiments

All NMR spectra were recorded at 5°C on a Bruker 800 MHz spectrometer equipped with a cryogenic probe and Z-field gradient using Bruker Topspin v4.0.7, recording ^{15}N,^{1}H-HSQC spectra to address interaction and using the backbone assignments obtained in (Cholak *et al*, 2020). ^{15}N-HSQC spectra were obtained in HEPES buffer (20 mM HEPES pH 7.4, 150 mM NaCl) of 50 μM ^{15}N-aSN alone and in the presence of $PMCA2w_{A\text{-}TM3}$ loop (molar ratio 1:5) and with further addition of 25 mM $CaCl_2$ (molar ratios 1:4:500). Spectra of 9.5 μM ^{15}N-aSN alone and with full-length PMCA2w/a (molar ratio 1:1) were obtained in Tris buffer (50 mM Tris pH 7.5, 150 mM KCl, 1.5% (w/v) DDM). Combined amide (N, H^N) chemical shift perturbations (CSPs) were measured by CCP-analysis version 2.5. The spectral analyses were done in CcpNMR analysis (Vranken *et al*, 2005).

Far-UV CD spectropolarimetry

Far UV CD spectra were recorded at 5°C on a JASCO J-815 spectropolarimeter on 12.8 μM $PMCA2w_{A\text{-}TM3}$ loop dissolved in 10 mM Na_2HPO_4, 200 mM NaF pH 7.4. The spectrum was recorded from 260 to 190 nm, averaging 10 scans (D.I.T 2 s; data pitch 0.2 nm; band width 1 nm; scan rate 50 mm/min; path length 1 mm). A spectrum of the buffer was recorded using identical settings and subtracted.

SERCA and Na, K-ATPase preparations

SERCA1a from rabbit skeletal muscle was kindly donated by Thomas Lykke-Møller Sørensen, Anne Lillevang, and Claus Olesen. The protein preparation was performed according to previously established procedures (Andersen *et al*, 1985; Sorensen *et al*, 2004). Na, K-ATPase was kindly donated by Michael Habeck and Natalya Fedosova. The protein was expressed and purified as described earlier (Habeck *et al*, 2017).

ATPase activity assay

The ATPase activity was tested by monitoring the release of free inorganic phosphate from ATP in a molybdenum blue assay (Baginski *et al*, 1967).

Purified PMCA requires relipidation to regain activity; therefore, the samples were preincubated with phospholipids before activity assay. Lipid stock solutions were prepared from powdered lipids as follows: brain lipid extract (from bovine brain Type I, Folch Fraction I, Sigma-Aldrich), brain PC (Avanti), or soy PC (Avanti) was dissolved to a concentration of 20 mg/ml in a buffer containing 50 mM Tris pH 7.5, 150 mM KCl, and 1.5% (w/v) DDM. After cycles of heating (45–50°C), sonicating, and vortexing, a translucent gel was obtained. Stocks were aliquoted and stored in −20°C. Shortly before the assay lipid solution was added to PMCA sample in PMCA:lipid w/w ratio 1:5 and mixed by gentle pipetting. After 15 min, the relipidated protein was added to a final concentration of 2.5 μg/ml to a reaction buffer containing 50 mM KNO_3, 5 mM NaN_3, 0.25 mM NaMob, 40 mM BisTris/HEPES pH 7.2, 3 mM $MgSO_4$, 1 mM EGTA, and $CaCl_2$ in concentration needed for obtaining the desired concentration of free calcium ions. The free calcium concentration was calculated using Maxchelator Ca/Mg/ATP/EGTA Calculator v1.0 using constants from NIST database (https://somapp.ucdmc.ucdavis.edu/pharmacology/bers/maxchelator). aSN and/or CaM were added to the reaction mixture in desired concentrations and corresponding protein storage buffer was added to control samples in equal volume. The reactions were started by adding 3 mM ATP, carried out at 37°C, and stopped after 7 or 11 min by adding a stop solution. Stop solution was prepared shortly before every assay by mixing ice-cold solutions A (17 μM ascorbic acid, 0.1% SDS in 0.5 M HCl) and B (0.7 μM ammonium molybdate VI tetrahydrate) in 5:1 proportion. The absorbance was measured with Victor 3 96-well plate reader (PerkinElmer) at 860 nm.

Mathematical modeling of aSN-dependent calcium regulation

The *solve_ivp* function from the *scipy* (Virtanen *et al*, 2020) package was used for numerical integration of the model equations to simulate the model behavior. Simulation results were visualized using the *matplotlib* (Hunter, 2007) library to create informative plots and figures. To optimize the parameters of the kinetic equation used for PMCA, the *scipy.minimize* function was employed, minimizing the discrepancy of the equation to the experimental data. With respect to the modeling work presented here, data handling tasks were performed using the *pandas* (McKinney, 2010; Data ref: Reback *et al*, 2022) and *numpy* (Harris *et al*, 2020) libraries.

A detailed method with parameter table (Appendix Table S1) can be found in the Appendix Supplementary Methods.

Transcriptomics data analysis

Data on gene expression for PMCA1-4 and aSN were used. Data on tissue expression and exon incorporation from VastDB for the genes ATP2B1, ATP2B2, ATP2B3, ATP2B4, and SNCA were imported in R. For the PMCA exons of relevance, the "percent spliced-in" (PSI) was correlated to aSN tissue expression. For tissues in specified aSN expression intervals (ranging from 0 to 190 interval size of 10), the average incorporation of the exons was determined, and the mean PSI was plotted as a function of the aSN expression interval. The code written in R for the analysis is included in the Appendix S1. Links to analyzed VastDB datasets are provided in the Expanded View Table EV1.

Protein disorder prediction

Protein disorder prediction was performed using NetSurfP 2.0 software developed by the Technical University of Denmark and available at https://services.healthtech.dtu.dk/service.php?NetSurfP-2.0.

© 2023 The Authors

Statistical analysis

Statistics were performed using GraphPad Prism and P values < 0.05 were considered significant. The statistical method used is described in the figure legends.

Data availability

All plasmids and DNA primers used in this study are available from the Lead Contact (Poul Nissen) without restriction. Reagents used in the study were of general use and from commercial sources. This study includes no data deposited in external repositories. The transcriptomics data are publicly available at https://vastdb.crg.eu/. All original code written in R for the analysis is available in this paper's Appendix S1. The mathematical modeling data are available at https://ford.biologie.hu-berlin.de/jorin/pmca_asn/-/tree/main/. The remaining data reported in this paper, including DNA sequences used in the study, will be shared by the lead contact upon request.

Expanded View for this article is available online.

Acknowledgements

This work was supported by a "Mobility plus, 3rd edition" fellowship grant of the Polish Ministry of Science and Higher Education to AK, a postdoctoral research grant from the Extremadura Province (PO17009) to MCB, by a PhD stipend from the Aarhus Graduate School of Science at Aarhus University to STL, by a collaborative grant from H. Lundbeck A/S to PHJ, by Lundbeck Foundation grants R223-2015-4222 for PHJ and R248-2016-2518 for Danish Research Institute of Translational Neuroscience-DANDRITE, Nordic-EMBL Partnership for Molecular to PN and PHJ, and by funds from a project 2 research grant from the Independent Research Fund Denmark (7014-00328B) and the Brainstruc research center (R155-2015-2666) and a professorship grant (R310-2018-3713) of the Lundbeck Foundation to PN. Further support was given by the European Union's Horizon 2020 research and innovation program under the Marie Sklodowska-Curie grant agreement No. 101023654, awarded to EAN. and the Novo Nordisk Foundation (#NNF18OC0033926 to BBK). All NMR data were recorded at cOpenNMR, an infrastructure facility funded by the Novo Nordisk Foundation (#NNF18OC0032996). The mathematical modeling work was funded by the Deutsche Forschungsgemeinschaft (DFG, German Research Foundation) under Germany's Excellence Strategy—The Berlin Mathematics Research Center MATH+ (EXC-2046/1, project ID: 390685689) to EK.

Acknowledgments

We thank Torben Heick Jensen and Thomas Gonatopoulos-Pournatzis for advice on transcriptomics data analysis. We are grateful for biosamples and valuable discussion to Michael Habeck for Na,K-ATPase, Thomas Lykke-Møller Sørensen, Anne Lillevang, and Claus Olesen for SERCA. We thank Joseph A Lyons and Magnus Kjærgaard for valuable discussions on membrane proteins and intrinsically disordered proteins. We are grateful to Anna Marie Nielsen, Tetyana Klymchuk, Benedicte Vestergaard, and Jacob H Martinsen for technical assistance, and we thank Mogens Høgh Jensen, Jesper Ferkinghoff-Borg, and Mathias S. Heltberg for valuable input on enzymatic network modeling.

Author contributions

Antoni Kowalski: Data curation; formal analysis; validation; investigation; visualization; methodology; writing – original draft; writing – review and editing. **Cristine Betzer:** Validation; investigation; visualization; writing – review and editing. **Sigrid Thirup Larsen:** Data curation; software; formal analysis; validation; investigation; visualization; writing – review and editing. **Emil Gregersen:** Validation; investigation; visualization; writing – review and editing. **Estella A Newcombe:** Investigation; visualization; methodology; writing – review and editing. **Montaña Caballero Bermejo:** Investigation. **Annette Eva Langkilde:** Resources; formal analysis; supervision; writing – review and editing. **Birthe B Kragelund:** Resources; data curation; formal analysis; supervision; funding acquisition; validation; methodology; writing – review and editing. **Poul Henning Jensen:** Conceptualization; resources; data curation; formal analysis; supervision; funding acquisition; validation; methodology; project administration; writing – review and editing. **Poul Nissen:** Conceptualization; resources; data curation; formal analysis; supervision; funding acquisition; validation; methodology; writing – original draft; project administration; writing – review and editing. **Shweta Jain:** Investigation. **Robert Edwards:** Resources; data curation; formal analysis; funding acquisition; investigation; methodology. **Lene N Nejsum:** Resources; data curation; formal analysis; funding acquisition; investigation; methodology. **Christina V Ernstsen:** Investigation. **Viktor Wisniewski Bendtsen:** Software; formal analysis; investigation; methodology. **Jorin Diemer:** Data curation; software; formal analysis; investigation; methodology; writing – review and editing. **Edda Klipp:** Resources; data curation; software; formal analysis; supervision; funding acquisition; validation; methodology; project administration. **Alicia Espiña Bou:** Investigation.

Disclosure and competing interests statement

Poul Nissen is a member of the Advisory Editorial Board of The EMBO Journal. This has no bearing on the editorial consideration of this article for publication.

References

Adamo HP, Penniston JT (1992) New Ca^{2+} pump isoforms generated by alternative splicing of rPMCA2 mRNA. *Biochem J* 283: 355–359

Andersen JP, Lassen K, Moller JV (1985) Changes in Ca^{2+} affinity related to conformational transitions in the phosphorylated state of soluble monomeric Ca^{2+}-ATPase from sarcoplasmic reticulum. *J Biol Chem* 260: 371–380

Antalffy G, Caride AJ, Paszty K, Hegedus L, Padanyi R, Strehler EE, Enyedi A (2011) Apical localization of PMCA2w/b is enhanced in terminally polarized MDCK cells. *Biochem Biophys Res Commun* 410: 322–327

Augustine GJ, Santamaria F, Tanaka K (2003) Local calcium signaling in neurons. *Neuron* 40: 331–346

Axelsen KB, Palmgren MG (2001) Inventory of the superfamily of P-type ion pumps in *Arabidopsis*. *Plant Physiol* 126: 696–706

Baginski ES, Foa PP, Zak B (1967) Microdetermination of inorganic phosphate, phospholipids, and total phosphate in biologic materials. *Clin Chem* 13: 326–332

Baici A (2015) *Kinetics of enzyme-modifier interactions: selected topics in the theory and diagnosis of inhibition and activation mechanisms*. Vienna: Springer

Barbour R, Kling K, Anderson JP, Banducci K, Cole T, Diep L, Fox M, Goldstein JM, Soriano F, Seubert P *et al* (2008) Red blood cells are the major source of alpha-synuclein in blood. *Neurodegener Dis* 5: 55–59

Berrocal M, Corbacho I, Sepulveda MR, Gutierrez-Merino C, Mata AM (2017) Phospholipids and calmodulin modulate the inhibition of PMCA activity by tau. *Biochim Biophys Acta Mol Cell Res* 1864: 1028–1035

 © 2023 The Authors

Betzer C, Movius AJ, Shi M, Gai WP, Zhang J, Jensen PH (2015) Identification of synaptosomal proteins binding to monomeric and oligomeric alpha-synuclein. *PLoS One* 10: e0116473

Betzer C, Lassen LB, Olsen A, Kofoed RH, Reimer L, Gregersen E, Zheng J, Cali T, Gai WP, Chen T *et al* (2018) Alpha-synuclein aggregates activate calcium pump SERCA leading to calcium dysregulation. *EMBO Rep* 19: e44617

Bodis-Wollner I, Kozlowski PB, Glazman S, Miri S (2014) alpha-Synuclein in the inner retina in parkinson disease. *Ann Neurol* 75: 964–966

Bodner CR, Dobson CM, Bax A (2009) Multiple tight phospholipid-binding modes of alpha-synuclein revealed by solution NMR spectroscopy. *J Mol Biol* 390: 775–790

Bogdanova A, Makhro A, Wang J, Lipp P, Kaestner L (2013) Calcium in red blood cells-a perilous balance. *Int J Mol Sci* 14: 9848–9872

Bond GH, Hudgins PM (1980) Inhibition of red cell Ca^{2+}-ATPase by vanadate. *Biochim Biophys Acta* 600: 781–790

Boura E, Hurley JH (2012) Structural basis for membrane targeting by the MVB12-associated beta-prism domain of the human ESCRT-I MVB12 subunit. *Proc Natl Acad Sci USA* 109: 1901–1906

Brini M, Di Leva F, Ortega CK, Domi T, Ottolini D, Leonardi E, Tosatto SCE, Carafoli E (2010) Deletions and mutations in the acidic lipid-binding region of the plasma membrane Ca^{2+} pump: a study on different splicing variants of isoform 2. *J Biol Chem* 285: 30779–30791

Brodin P, Falchetto R, Vorherr T, Carafoli E (1992) Identification of two domains which mediate the binding of activating phospholipids to the plasma-membrane Ca^{2+} pump. *Eur J Biochem* 204: 939–946

Burre J, Sharma M, Tsetsenis T, Buchman V, Etherton MR, Sudhof TC (2010) alpha-Synuclein promotes SNARE-complex assembly in vivo and in vitro. *Science* 329: 1663–1667

Burre J, Sharma M, Sudhof TC (2018) Cell biology and pathophysiology of alpha-synuclein. *Cold Spring Harb Perspect Med* 8: a024091

Cali T, Ottolini D, Brini M (2014) Calcium signaling in Parkinson's disease. *Cell Tissue Res* 357: 439–454

Cali T, Brini M, Carafoli E (2017) Regulation of cell calcium and role of plasma membrane calcium ATPases. *Int Rev Cell Mol Biol* 332: 259–296

Cali T, Brini M, Carafoli E (2018) The PMCA pumps in genetically determined neuronal pathologies. *Neurosci Lett* 663: 2–11

Cantley LC Jr, Cantley LG, Josephson L (1978) A characterization of vanadate interactions with the (Na,K)-ATPase. Mechanistic and regulatory implications. *J Biol Chem* 253: 7361–7368

Carafoli E (1994) Biogenesis: plasma membrane calcium ATPase: 15 years of work on the purified enzyme. *FASEB J* 8: 993–1002

Caride AJ, Elwess NL, Verma AK, Filoteo AG, Enyedi A, Bajzer Z, Penniston JT (1999) The rate of activation by calmodulin of isoform 4 of the plasma membrane Ca^{2+} pump is slow and is changed by alternatives splicing. *J Biol Chem* 274: 35227–35232

Caride AJ, Filoteo AG, Penniston JT, Strehler EE (2007) The plasma membrane Ca^{2+} pump isoform 4a differs from isoform 4b in the mechanism of calmodulin binding and activation kinetics: implications for Ca^{2+} signaling. *J Biol Chem* 282: 25640–25648

Chamberland S, Timofeeva Y, Evstratova A, Norman CA, Volynski K, Tóth K (2020) Slow-decaying presynaptic calcium dynamics gate long-lasting asynchronous release at the hippocampal mossy fiber to CA3 pyramidal cell synapse. *Synapse* 74: e22178

Chen HY, Chesler M (2015) Autocrine boost of NMDAR current in hippocampal CA1 pyramidal neurons by a PMCA-dependent, perisynaptic, extracellular pH shift. *J Neurosci* 35: 873–877

Cheng F, Vivacqua G, Yu S (2011) The role of alpha-synuclein in neurotransmission and synaptic plasticity. *J Chem Neuroanat* 42: 242–248

Cholak E, Bugge K, Khondker A, Gauger K, Pedraz-Cuesta E, Pedersen ME, Bucciarelli S, Vestergaard B, Pedersen SF, Rheinstadter MC *et al* (2020) Avidity within the N-terminal anchor drives alpha-synuclein membrane interaction and insertion. *FASEB J* 34: 7462–7482

Cunningham KW, Fink GR (1994) Calcineurin-dependent growth control in *Saccharomyces cerevisiae* mutants lacking PMC1, a homolog of plasma membrane Ca^{2+} ATPases. *J Cell Biol* 124: 351–363

Danzer KM, Haasen D, Karow AR, Moussaud S, Habeck M, Giese A, Kretzschmar H, Hengerer B, Kostka M (2007) Different species of alpha-synuclein oligomers induce calcium influx and seeding. *J Neurosci* 27: 9220–9232

Davidson WS, Jonas A, Clayton DF, George JM (1998) Stabilization of alpha-synuclein secondary structure upon binding to synthetic membranes. *J Biol Chem* 273: 9443–9449

Dettmer U, Newman AJ, Luth ES, Bartels T, Selkoe D (2013) *In vivo* cross-linking reveals principally oligomeric forms of alpha-synuclein and beta-synuclein in neurons and non-neural cells. *J Biol Chem* 288: 6371–6385

Dettmer U, Newman AJ, Soldner F, Luth ES, Kim NC, von Saucken VE, Sanderson JB, Jaenisch R, Bartels T, Selkoe D (2015a) Parkinson-causing alpha-synuclein missense mutations shift native tetramers to monomers as a mechanism for disease initiation. *Nat Commun* 6: 7314

Dettmer U, Newman AJ, von Saucken VE, Bartels T, Selkoe D (2015b) KTKEGV repeat motifs are key mediators of normal alpha-synuclein tetramerization: their mutation causes excess monomers and neurotoxicity. *Proc Natl Acad Sci USA* 112: 9596–9601

Di Leva F, Domi T, Fedrizzi L, Lim D, Carafoli E (2008) The plasma membrane Ca^{2+} ATPase of animal cells: structure, function and regulation. *Arch Biochem Biophys* 476: 65–74

Di Scala C, Yahi N, Boutemeur S, Flores A, Rodriguez L, Chahinian H, Fantini J (2016) Common molecular mechanism of amyloid pore formation by Alzheimer's beta-amyloid peptide and alpha-synuclein. *Sci Rep* 6: 28781

Dikiy I, Eliezer D (2012) Folding and misfolding of alpha-synuclein on membranes. *Biochim Biophys Acta Biomembranes* 1818: 1013–1018

Domi T, Di Leva F, Fedrizzi L, Rimessi A, Brini M (2007) Functional specificity of PMCA isoforms? *Ann N Y Acad Sci* 1099: 237–246

Drew D, Newstead S, Sonoda Y, Kim H, von Heijne G, Iwata S (2008) GFP-based optimization scheme for the overexpression and purification of eukaryotic membrane proteins in *Saccharomyces cerevisiae*. *Nat Protoc* 3: 784–798

Dupont Y, Bennett N (1982) Vanadate inhibition of the Ca^{2+}—dependent conformational change of the sarcoplasmic reticulum Ca^{2+}-ATPase. *FEBS Lett* 139: 237–240

Eliezer D, Kutluay E, Bussell R Jr, Browne G (2001) Conformational properties of alpha-synuclein in its free and lipid-associated states. *J Mol Biol* 307: 1061–1073

Enyedi A, Strehler EE (2011) Regulation of apical membrane enrichment and retention of plasma membrane Ca ATPase splice variants by the PDZ-domain protein NHERF2. *Commun Integr Biol* 4: 340–343

Erler F, Meyer-Hermann M, Soff G (2004) A quantitative model for presynaptic free Ca^{2+} dynamics during different stimulation protocols. *Neurocomputing* 61: 169–191

Ernstsen CV, Login FH, Schelde AB, Therkildsen JR, Moller-Jensen J, Norregaard R, Praetorius H, Nejsum LN (2022) Acute pyelonephritis: increased plasma membrane targeting of renal aquaporin-2. *Acta Physiol (Oxf)* 234: e13760

Fanning S, Selkoe D, Dettmer U (2020) Parkinson's disease: proteinopathy or lipidopathy? *NPJ Parkinsons Dis* 6: 3

Fauvet B, Mbefo MK, Fares MB, Desobry C, Michael S, Ardah MT, Tsika E, Coune P, Prudent M, Lion N *et al* (2012) alpha-Synuclein in central

nervous system and from erythrocytes, mammalian cells, and *Escherichia coli* exists predominantly as disordered monomer. *J Biol Chem* 287: 15345–15364

Feghhi T, Hernandez RX, Stawarski M, Thomas CI, Kamasawa N, Lau AWC, Macleod GT (2021) Computational modeling predicts ephemeral acidic microdomains in the glutamatergic synaptic cleft. *Biophys J* 120: 5575–5591

Folch J, Lees M, Sloane Stanley GH (1957) A simple method for the isolation and purification of total lipides from animal tissues. *J Biol Chem* 226: 497–509

Gietz RD, Schiestl RH (2007) High-efficiency yeast transformation using the LiAc/SS carrier DNA/PEG method. *Nat Protoc* 2: 31–34

Gong D, Chi X, Ren K, Huang G, Zhou G, Yan N, Lei J, Zhou Q (2018) Structure of the human plasma membrane Ca(2+)-ATPase 1 in complex with its obligatory subunit neuroplastin. *Nat Commun* 9: 3623

Habeck M, Kapri-Pardes E, Sharon M, Karlish SJ (2017) Specific phospholipid binding to Na,K-ATPase at two distinct sites. *Proc Natl Acad Sci USA* 114: 2904–2909

Harris CR, Millman KJ, van der Walt SJ, Gommers R, Virtanen P, Cournapeau D, Wieser E, Taylor J, Berg S, Smith NJ *et al* (2020) Array programming with NumPy. *Nature* 585: 357–362

Hayashi J, Carver JA (2022) beta-Synuclein: an enigmatic protein with diverse functionality. *Biomolecules* 12: 142

Hodgkin AL, Huxley AF (1952) A quantitative description of membrane current and its application to conduction and excitation in nerve. *J Physiol* 117: 500–544

Huang C, Ren G, Zhou H, Wang CC (2005) A new method for purification of recombinant human alpha-synuclein in *Escherichia coli*. *Protein Expr Purif* 42: 173–177

Hunter JD (2007) Matplotlib: a 2D graphics environment. *Comput Sci Eng* 9: 90–95

Hurley MJ, Brandon B, Gentleman SM, Dexter DT (2013) Parkinson's disease is associated with altered expression of Ca(V)1 channels and calcium-binding proteins. *Brain* 136: 2077–2097

Hurley MJ, Gentleman SM, Dexter DT (2015) Calcium CaV1 channel subtype mRNA expression in Parkinson's disease examined by in situ hybridization. *J Mol Neurosci* 55: 715–724

Jensen PH, Nielsen MS, Jakes R, Dotti G, Goedert M (1998) Binding of alpha-synuclein to brain vesicles is abolished by familial Parkinson's disease mutation. *J Biol Chem* 273: 26292–26294

Jensen PH, Islam K, Kenney J, Nielsen MS, Power J, Gai WP (2000) Microtubule-associated protein 1B is a component of cortical Lewy bodies and binds alpha-synuclein filaments. *J Biol Chem* 275: 21500–21507

Jo EJ, McLaurin J, Yip CM, St George-Hyslop P, Fraser PE (2000) alpha-Synuclein membrane interactions and lipid specificity. *J Biol Chem* 275: 34328–34334

Jo E, Fuller N, Rand RP, St George-Hyslop P, Fraser PE (2002) Defective membrane interactions of familial Parkinson's disease mutant A30P alpha-synuclein. *J Mol Biol* 315: 799–807

Keener J, Sneyd J (2009) *Mathematical Physiology I: Cellular Physiology*, Vol. 8/1. New York: Springer

Kessler F, Falchetto R, Heim R, Meili R, Vorherr T, Strehler EE, Carafoli E (1992) Study of calmodulin binding to the alternatively spliced C-terminal domain of the plasma membrane Ca^{2+} pump. *Biochemistry* 31: 11785–11792

Kragh CL, Lund LB, Febbraro F, Hansen HD, Gai W-P, El-Agnaf O, Richter-Landsberg C, Jensen PH (2009) α-Synuclein aggregation and Ser-129 phosphorylation-dependent cell death in oligodendroglial cells. *J Biol Chem* 284: 10211–10222

Krebs J (2015) The plethora of PMCA isoforms: alternative splicing and differential expression. *Biochim Biophys Acta* 1853: 2018–2024

Krick N, Ryglewski S, Pichler A, Bikbaev A, Gotz T, Kobler O, Heine M, Thomas U, Duch C (2021) Separation of presynaptic Cav2 and Cav1 channel function in synaptic vesicle exo- and endocytosis by the membrane anchored Ca(2+) pump PMCA. *Proc Natl Acad Sci USA* 118: e2106621118

Krizaj D, Demarco SJ, Johnson J, Strehler EE, Copenhagen DR (2002) Cell-specific expression of plasma membrane calcium ATPase isoforms in retinal neurons. *J Comp Neurol* 451: 1–21

Kruger R, Kuhn W, Leenders KL, Sprengelmeyer R, Muller T, Woitalla D, Portman AT, Maguire RP, Veenma L, Schroder U *et al* (2001) Familial parkinsonism with synuclein pathology: clinical and PET studies of A30P mutation carriers. *Neurology* 56: 1355–1362

Lautenschlager J, Stephens AD, Fusco G, Strohl F, Curry N, Zacharopoulou M, Michel CH, Laine R, Nespovitaya N, Fantham M *et al* (2018) C-terminal calcium binding of alpha-synuclein modulates synaptic vesicle interaction. *Nat Commun* 9: 712

Lindersson E, Beedholm R, Højrup P, Moos T, Gai W, Hendil KB, Jensen PH (2004) Proteasomal inhibition by α-synuclein filaments and oligomers. *J Biol Chem* 279: 12924–12934

Liu H, Naismith JH (2008) An efficient one-step site-directed deletion, insertion, single and multiple-site plasmid mutagenesis protocol. *BMC Biotechnol* 8: 91

Long AA, Kim E, Leung HT, Woodruff E 3rd, An L, Doerge RW, Pak WL, Broadie K (2008) Presynaptic calcium channel localization and calcium-dependent synaptic vesicle exocytosis regulated by the fuseless protein. *J Neurosci* 28: 3668–3682

Lopreiato R, Giacomello M, Carafoli E (2014) The plasma membrane calcium pump: new ways to look at an old enzyme. *J Biol Chem* 289: 10261–10268

McKinney W (2010) Data structures for statistical computing in Python. In *Proceedings of the 9th Python in Science Conference* https://doi.org/10.25080/majora-92bf1922-00a

Morton AJ, Hammond C, Mason WT, Henderson G (1992) Characterisation of the L- and N-type calcium channels in differentiated SH-SY5Y neuroblastoma cells: calcium imaging and single channel recording. *Mol Brain Res* 13: 53–61

Newcombe EA, Fernandes CB, Lundsgaard JE, Brakti I, Lindorff-Larsen K, Langkilde AE, Skriver K, Kragelund BB (2021) Insight into calcium-binding motifs of intrinsically disordered proteins. *Biomolecules* 11: 1173

Nielsen MS, Vorum H, Lindersson E, Jensen PH (2001) Ca^{2+} binding to alpha-synuclein regulates ligand binding and oligomerization. *J Biol Chem* 276: 22680–22684

Niggli V, Adunyah ES, Carafoli E (1981a) Acidic phospholipids, unsaturated fatty acids, and limited proteolysis mimic the effect of calmodulin on the purified erythrocyte Ca^{2+} – ATPase. *J Biol Chem* 256: 8588–8592

Niggli V, Adunyah ES, Penniston JT, Carafoli E (1981b) Purified (Ca^{2+}-Mg^{2+})-ATPase of the erythrocyte membrane. Reconstitution and effect of calmodulin and phospholipids. *J Biol Chem* 256: 395–401

Oliveira LMA, Gasser T, Edwards R, Zweckstetter M, Melki R, Stefanis L, Lashuel HA, Sulzer D, Vekrellis K, Halliday GM *et al* (2021) Alpha-synuclein research: defining strategic moves in the battle against Parkinson's disease. *NPJ Parkinsons Dis* 7: 65

Pei Y, Maitta RW (2019) Alpha synuclein in hematopoiesis and immunity. *Heliyon* 5: e02590

Penniston JT, Padanyi R, Paszty K, Varga K, Hegedus L, Enyedi A (2014) Apart from its known function, the plasma membrane Ca(2)(+)ATPase can

 © 2023 The Authors

regulate Ca(2)(+) signaling by controlling phosphatidylinositol 4,5-bisphosphate levels. *J Cell Sci* 127: 72–84

Perni M, Galvagnion C, Maltsev A, Meisl G, Muller MBD, Challa PK, Kirkegaard JB, Flagmeier P, Cohen SIA, Cascella R *et al* (2017) A natural product inhibits the initiation of alpha-synuclein aggregation and suppresses its toxicity (vol 114, pg E1009, 2017). *Proc Natl Acad Sci USA* 114: E2543

Pinto Fde T, Adamo HP (2002) Deletions in the acidic lipid-binding region of the plasma membrane Ca^{2+} pump. A mutant with high affinity for Ca^{2+} resembling the acidic lipid-activated enzyme. *J Biol Chem* 277: 12784–12789

Rcom-H'cheo-Gauthier AN, Osborne SL, Meedeniya AC, Pountney DL (2016) Calcium: alpha-synuclein interactions in alpha-synucleinopathies. *Front Neurosci* 10: 570

Reback J, jbrockmendel, McKinney W, den Bossche JV, Augspurger T, Roeschke M, Hawkins S, Cloud P, gfyoung, Sinhrks *et al* (2022) pandas-dev/pandas: Pandas 1.4.2. *Zenodo* (https://zenodo.org/records/6408044) [DATASET]

Reimer L, Haikal C, Gram H, Theologidis V, Kovacs G, Ruesink H, Baun A, Nielsen J, Otzen DE, Li J-Y *et al* (2022) Low dose DMSO treatment induces oligomerization and accelerates aggregation of α-synuclein. *Sci Rep* 12: 3737

Schindelin J, Arganda-Carreras I, Frise E, Kaynig V, Longair M, Pietzsch T, Preibisch S, Rueden C, Saalfeld S, Schmid B *et al* (2012) Fiji: an open-source platform for biological-image analysis. *Nat Methods* 9: 676–682

Singh N, Bartol T, Levine H, Sejnowski T, Nadkarni S (2021) Presynaptic endoplasmic reticulum regulates short-term plasticity in hippocampal synapses. *Commun Biol* 4: 241

Skaanning LK, Santoro A, Skamris T, Martinsen JH, D'Ursi AM, Bucciarelli S, Vestergaard B, Bugge K, Langkilde AE, Kragelund BB (2020) The non-fibrillating N-terminal of alpha-synuclein binds and co-fibrillates with heparin. *Biomolecules* 10: 1192

Sorensen TL, Clausen JD, Jensen AM, Vilsen B, Moller JV, Andersen JP, Nissen P (2004) Localization of a K^{+} -binding site involved in dephosphorylation of the sarcoplasmic reticulum Ca^{2+} -ATPase. *J Biol Chem* 279: 46355–46358

Strehler EE (2015) Plasma membrane calcium ATPases: from generic Ca^{2+} sump pumps to versatile systems for fine-tuning cellular Ca^{2+}. *Biochem Biophys Res Commun* 460: 26–33

Strehler EE, Thayer SA (2018) Evidence for a role of plasma membrane calcium pumps in neurodegenerative disease: recent developments. *Neurosci Lett* 663: 39–47

Strehler EE, Zacharias DA (2001) Role of alternative splicing in generating isoform diversity among plasma membrane calcium pumps. *Physiol Rev* 81: 21–50

Strehler EE, Caride AJ, Filoteo AG, Xiong Y, Penniston JT, Enyedi A (2007a) Plasma membrane Ca^{2+} ATPases as dynamic regulators of cellular calcium handling. *Ann N Y Acad Sci* 1099: 226–236

Strehler EE, Filoteo AG, Penniston JT, Caride AJ (2007b) Plasma-membrane Ca (2+) pumps: structural diversity as the basis for functional versatility. *Biochem Soc Trans* 35: 919–922

Studier FW (2005) Protein production by auto-induction in high density shaking cultures. *Protein Expr Purif* 41: 207–234

Sulzer D, Edwards RH (2019) The physiological role of alpha-synuclein and its relationship to Parkinson's disease. *J Neurochem* 150: 475–486

Tapial J, Ha KCH, Sterne-Weiler T, Gohr A, Braunschweig U, Hermoso-Pulido A, Quesnel-Vallieres M, Permanyer J, Sodaei R, Marquez Y *et al* (2017) An atlas of alternative splicing profiles and functional associations reveals new regulatory programs and genes that simultaneously express multiple major isoforms. *Genome Res* 27: 1759–1768

Theillet FX, Binolfi A, Bekei B, Martorana A, Rose HM, Stuiver M, Verzini S, Lorenz D, van Rossum M, Goldfarb D *et al* (2016) Structural disorder of monomeric alpha-synuclein persists in mammalian cells. *Nature* 530: 45–50

Tidow H, Poulsen LR, Andreeva A, Knudsen M, Hein KL, Wiuf C, Palmgren MG, Nissen P (2012) A bimodular mechanism of calcium control in eukaryotes. *Nature* 491: 468–472

Tolosa de Talamoni N, Smith CA, Wasserman RH, Beltramino C, Fullmer CS, Penniston JT (1993) Immunocytochemical localization of the plasma membrane calcium pump, calbindin-D28k, and parvalbumin in Purkinje cells of avian and mammalian cerebellum. *Proc Natl Acad Sci USA* 90: 11949–11953

Ulmer TS, Bax A, Cole NB, Nussbaum RL (2005) Structure and dynamics of micelle-bound human alpha-synuclein. *J Biol Chem* 280: 9595–9603

Uversky VN, Eliezer D (2009) Biophysics of Parkinson's disease: structure and aggregation of alpha-synuclein. *Curr Protein Pept Sci* 10: 483–499

Vargas KJ, Schrod N, Davis T, Fernandez-Busnadiego R, Taguchi YV, Laugks U, Lucic V, Chandra SS (2017) Synucleins have multiple effects on presynaptic architecture. *Cell Rep* 18: 161–173

Vekrellis K, Xilouri M, Emmanouilidou E, Stefanis L (2009) Inducible over-expression of wild type alpha-synuclein in human neuronal cells leads to caspase-dependent non-apoptotic death. *J Neurochem* 109: 1348–1362

Virtanen P, Gommers R, Oliphant TE, Haberland M, Reddy T, Cournapeau D, Burovski E, Peterson P, Weckesser W, Bright J *et al* (2020) SciPy 1.0: fundamental algorithms for scientific computing in Python. *Nat Methods* 17: 261–272

Vranken WF, Boucher W, Stevens TJ, Fogh RH, Pajon A, Llinas M, Ulrich EL, Markley JL, Ionides J, Laue ED (2005) The CCPN data model for NMR spectroscopy: development of a software pipeline. *Proteins* 59: 687–696

Wu Y, Whiteus C, Xu CS, Hayworth KJ, Weinberg RJ, Hess HF, De Camilli P (2017) Contacts between the endoplasmic reticulum and other membranes in neurons. *Proc Natl Acad Sci USA* 114: E4859–E4867

Zvaritch E, James P, Vorherr T, Falchetto R, Modyanov N, Carafoli E (1990) Mapping of functional domains in the plasma membrane Ca^{2+} pump using trypsin proteolysis. *Biochemistry* 29: 8070–8076

License: This is an open access article under the terms of the Creative Commons Attribution License, which permits use, distribution and reproduction in any medium, provided the original work is properly cited.

Expanded View Figures

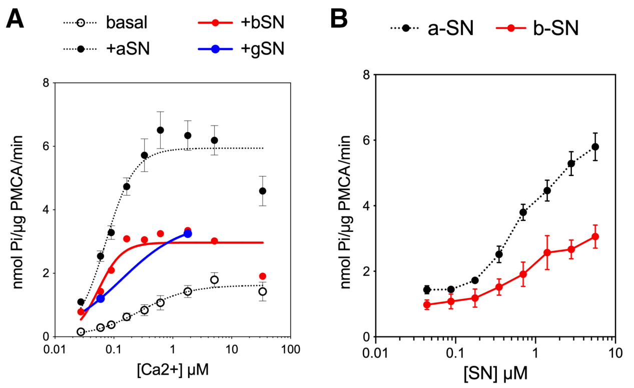

Figure EV1. PMCA1d activation by alpha-, beta-, and gamma-SN Beta- and gamma-synuclein stimulate PMCA but appear less potent than alpha-synuclein.

A Calcium titration measurement was performed with beta- (red) and gamma-synuclein (blue). PMCA1d was relipidated with brain lipid extract (BE). Data showing the activity without interaction partner and with alpha-synuclein are the same as in Fig 2, presented here for the comparison.

B Synuclein-titration experiment. Beta-synuclein titration was performed in presence of brain lipid extract (BE) in four technical replicates (n = 4). Data for alpha-synuclein titration are the same as in Fig 2, presented here for comparison. Data are mean ± SEM.

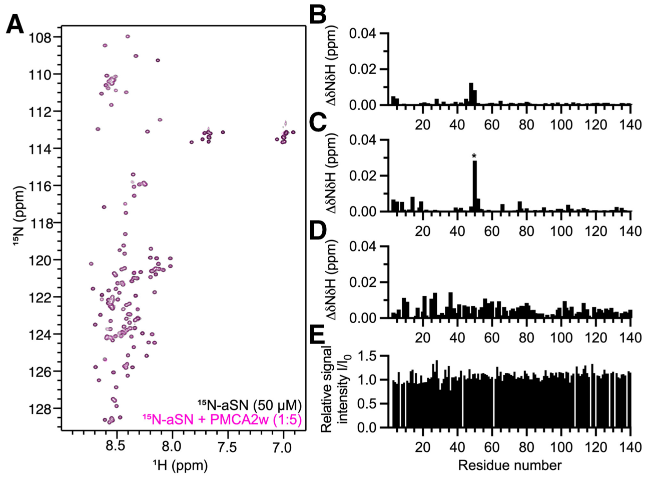

Figure EV2. NMR interaction studies.

A ^{15}N-HSQC spectra comparing ^{15}N-aSN alone (black) with that of ^{15}N-aSN added PMCA2w$_{A\text{-}TM3}$ loop (pink).

B Quantified amide chemical shift perturbations (CSPs) from (A).

C Amide CSPs caused by addition of PMCA2w$_{A\text{-}TM3}$ loop to ^{15}N-aSN in the presence of calcium (^{15}N-aSN:Ca^{2+} 1:500; ^{15}N-aSN:Ca^{2+}:PMCA2w 1:500:4). * indicates pH sensitivity observed for histidine.

D CSPs caused by full-length PMCA2w/a at a 1:1 molar ratio with ^{15}N-aSN.

E Peak intensity changes caused by addition of full-length PMCA2w/a at a 1:1 molar ratio with a^{15}N-aSN.

 © 2023 The Authors

Appendix

Table of Contents

Supplementary Figures 2

Appendix Figure S1. aSN increases calcium export from depolarized SH-SY5Y cells 2

Appendix Figure S2. Purification of recombinant PMCAs 3

Appendix Figure S3. Supplementary PMCA activity assays 4

Appendix Figure S4. Purity of alpha-synuclein preparations 5

Appendix Figure S5. Sequence alignment of the A-TM3 loop region and the TM3 domain of PMCA2w/aΔ298-372, PMCA2w/aΔ298-383, ACA8 and SERCA1a 5

Appendix Figure S6. Supplement to figure 1A – raw images of the western blots presented in Figure 1 6

Appendix Figure S7. Supplement to the figure 1B - pull-down of aSN with PMCA immobilized by Calmodulin-sepharose 7

Supplementary methods 8

A model for aSN-dependent calcium regulation 8

Appendix Table S1. Initial values and parameters for the calcium regulation model. ... 12

Appendix Figure S8. 14

References 14

Computer Code - Transcriptomics analysis 15

Supplementary Figures

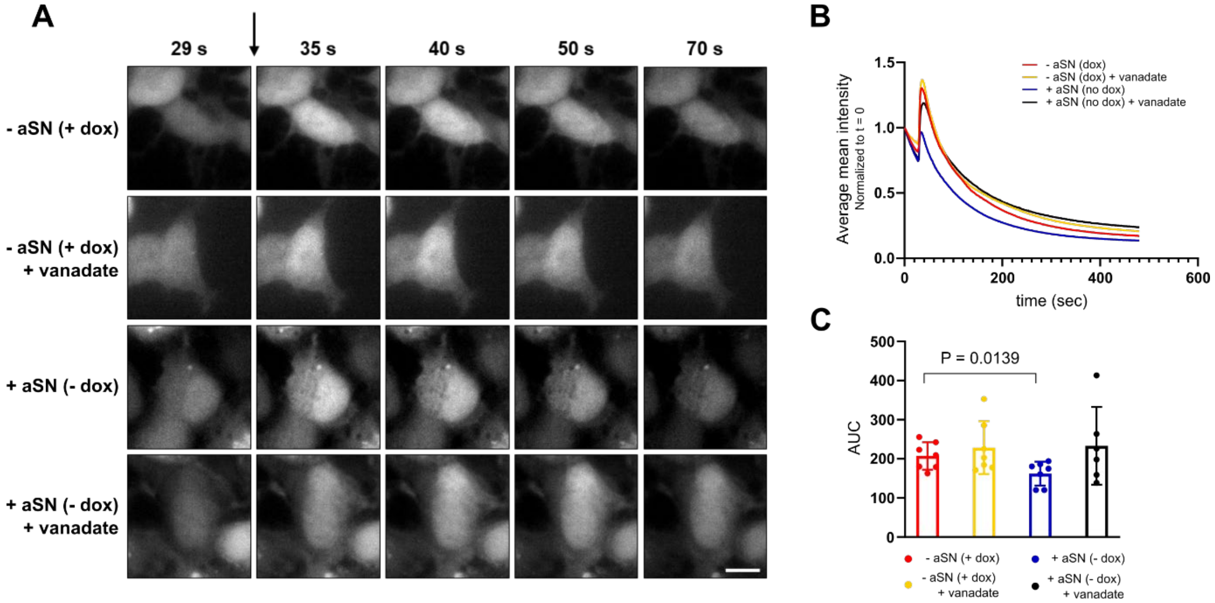

Appendix Figure S1. aSN increases calcium export from depolarized SH-SY5Y cells

Cytosolic calcium was monitored by Fluo-8 loading of SH-SY5Y cells with or without aSN. aSN expression was controlled by incubation with or without doxycycline (dox). Without dox present aSN is overexpressed and in the presence of dox aSN expression is repressed. Before recording, SERCA was inhibited by thapsigargin. Calcium influx was induced by addition of KCl. At the recording time of 30 sec. KCl was added to a final concentration of 90mM (indicated by arrows), depolarizing the SH-SY5Y cells. The calcium response was followed by time-lapse imaging.

A. Representative sequential images of Fluo-8 loaded cells at the time of depolarization. Arrow indicates time for addition of 90mM KCl and the time denoted is the experimental time. Scale bar: 10 µm.

B. Curve of average mean intensity from Fluo-8 loaded SH-SY5Y cells in: *blue* – SH-SY5Y cells without dox, expressing aSN, *black* - SH-SY5Y cells without dox, expressing aSN and treated with 1µM vanadate to inhibit ATPases, *red* - SH-SY5Y cells treated with dox, *yellow* - SH-SY5Y cells treated with dox and 1µM vanadate. The data is from 2 technical replicates with 2-4 cells per replicate, N (dox) = 8, N (dox + vanadate) = 7, N (- dox (aSN)) = 7, N (- dox (aSN) + vanadate) = 6.

C. Cytosolic Ca^{2+} level after the KCl-induced influx, quantified as the Area Under the Curve (AUC ± SEM). The response to KCl-induced influx was quantified as the Area Under Curve (AUC) from each measured SH-SY5Y cell. The data presented as mean ± SEM is from 2 technical replicates with 2-4 cells per replicate, N (dox) = 8, N (dox + vanadate) = 7, N (- dox (aSN)) = 7, N (- dox (aSN) + vanadate) = 6. The colors of the bars corresponds to the coloring of the curves in B. A two tailed students t-test was performed.

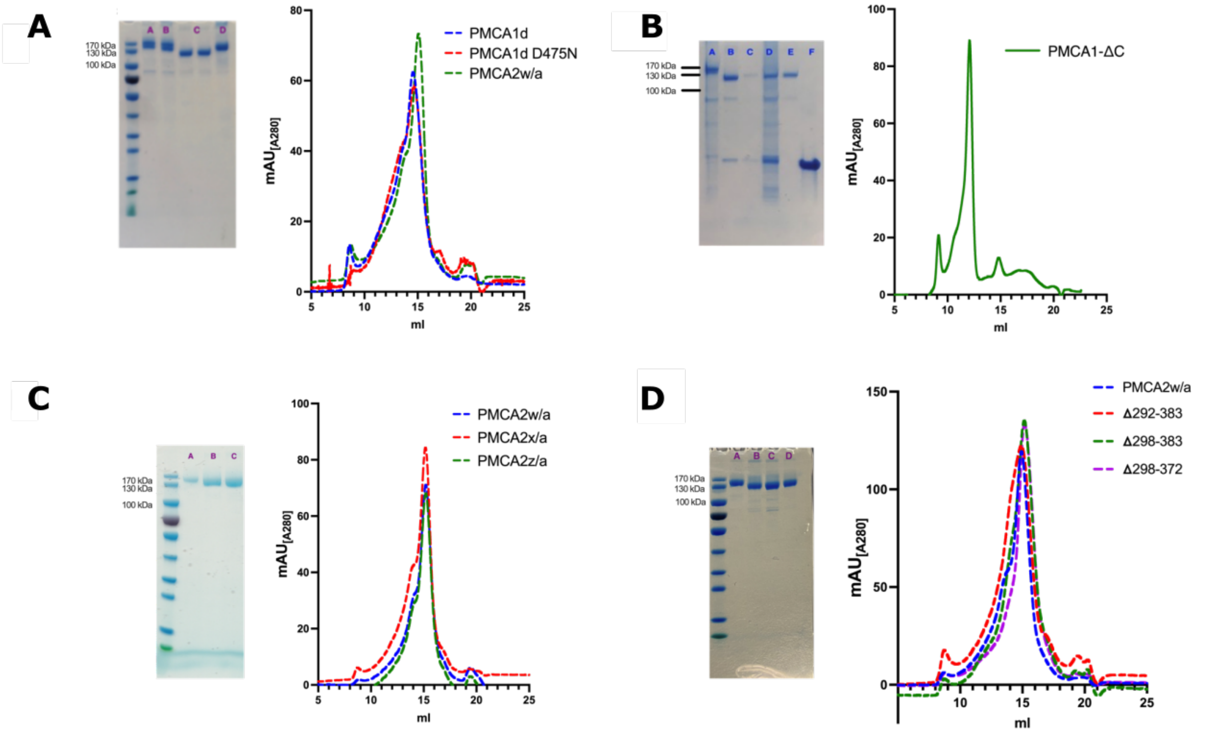

Appendix Figure S2. Purification of recombinant PMCAs

A - *Left* - SDS PAGE of purified PMCAs. (A) PMCA1d, PMCA1d D475N (inactive variant), PMCA1-ΔC, (D) PMCA2w/a. *Right* – Corresponding profiles of the size exclusion chromatography performed on the Superose 6 increase column.

B – *Left* – SDS-PAGE analysis of the purification of the PMCA1-ΔC. Full PMCA1d with internal TEV cleavage site at position 1074 was bound to CaM sepharose and washed (lane A – a sample of washed beads). After overnight incubation with His-tagged TEV flow-through 1 (FT1, lane B) was collected and beads were washed with buffer in a gravity-flow column (lane C - wash). FT1 was put through a Ni-Sepharose in a gravity-flow column to bind TEV and uncleaved PMCA1 (D – sample of washed sepharose beads). The flow-through from the nickel column was collected, concentrated to 500 µL, and subjected to size exclusion chromatography on Superdex 200 column. Lane E – peak fraction from the size-exclusion chromatography. Lane F – TEV protease. *Right* – Profile of the size exclusion chromatography of PMCA1-ΔC performed on the Superdex 200 increase column.

C – *Left* - SDS PAGE of purified PMCA2 splice variants (A-C) w/a, x/a, z/a. *Right* – Corresponding profiles of the size exclusion chromatography performed on the Superose 6 increase column.

D - *Left* - SDS PAGE of purified PMCA2w/a (A) and its deletion variants Δ292-383 (B), Δ298-383 (C), and Δ298-372 (D). *Right* – Corresponding profiles of the size exclusion chromatography performed on the Superose 6 increase column.

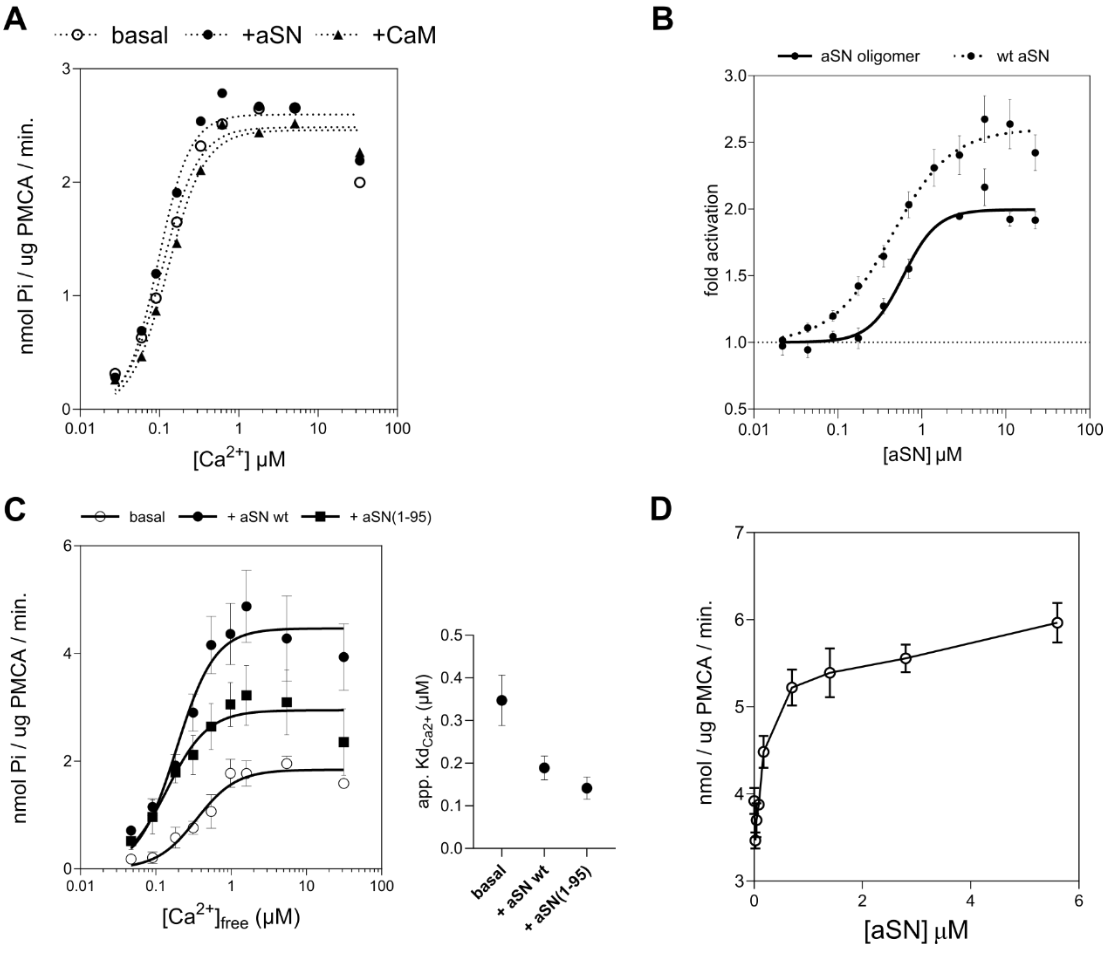

Appendix Figure S3. Supplementary PMCA activity assays

A. The activity of PMCA1-ΔC relipidated in brain PC lipids. Ca^{2+} titrations were performed in parallel in absence of the activating partner (basal activity), in the presence of 2.8 μM alpha-synuclein or in the presence of 1.2 μM CaM.

B. The effect of oligomeric aSN on the PMCA activity. PMCA1d fold activation by titrated full-length monomeric and oligomeric aSN. The assay was performed in presence of 1.8 μM free Ca^{2+} and brain lipid extract was used for the PMCA relipidation. Monomeric aSN data, presented here for comparison, is the same as in Figure 4. Oligomeric aSN experiment was performed in four independent replicates, where PMCA originated from single expression culture and aSN oligomers from two independent preparations.

All data shown as mean ± SEM.

C. The effect of full-length and C-terminally truncated $aSN_{(1\text{-}95)}$ on the calcium-dependent activity of PMCA1d. *On the left* – the calcium titration experiment was performed simultaneously in absence of activating partner (*empty circles*), in presence of the full-length monomeric a-SN (*filled circles*) and C-terminally truncated $aSN_{(1\text{-}95)}$ (*filled squares*). The pump was relipidated in brain lipid extract (BE). The lines are the best fit given by the Hill equation. *On the right* – apparent Kd_{Ca2+} values calculated from the fitted Hill plots with the apparent Kd values (μM) for Ca^{2+} as follows: PMCA1d basal (without aSN) – 0.347 ± 0.059, with aSNwt - 0.189 ± 0.028, with $aSN_{(1\text{-}95)}$ – 0.142 ± 0.026. The measurements were performed in three independent replicates, where the proteins originated from single expression culture.

D. The activity of C-terminally truncated PMCA4x (lacking the autoinhibitory domain) was measured as a function of monomeric a-SN concentration in presence of 1.8 μM free Ca^{2+}. PMCA was relipidated with brain extract (BE). The measurement was performed in independent duplicates, where proteins originated from single expression culture

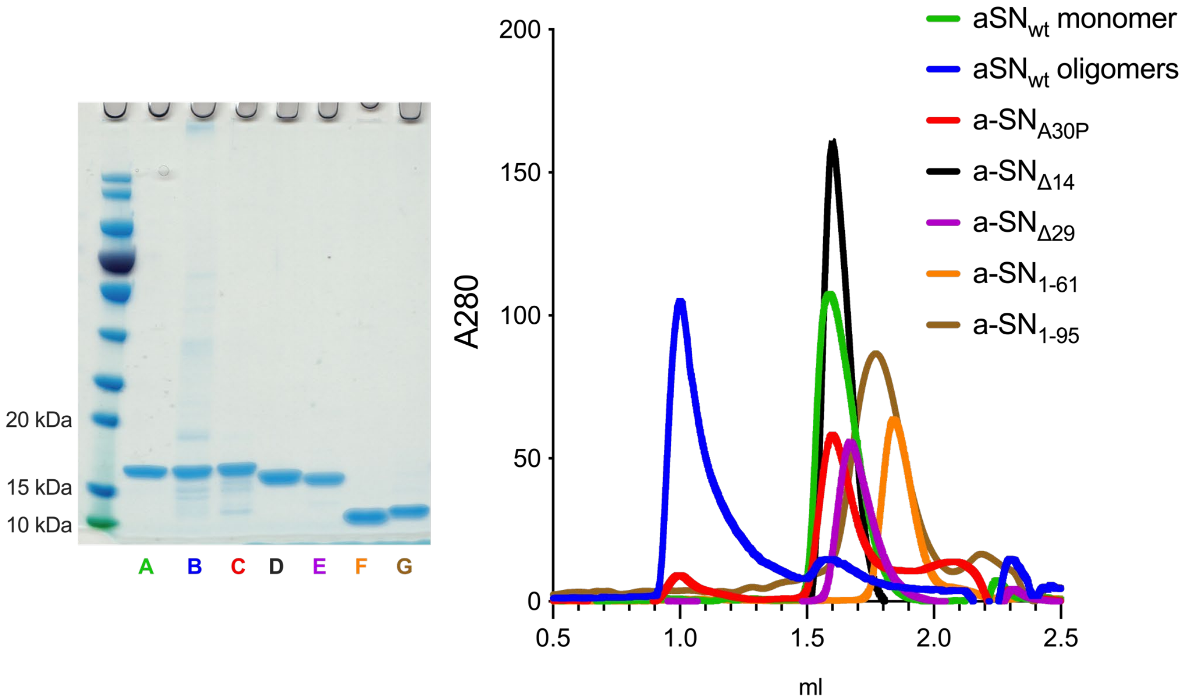

Appendix Figure S4. Purity of alpha-synuclein preparations.

On the left – SDS-PAGE analysis A – monomer; B – oligomers; C – A30P; D – Δ14; E – Δ29; F – (1-61); G – (1-95); *On the right* – size exclusion chromatography analysis performed on Superdex 200 increase 3.2/300 column. Monomeric forms were eluted at volume > 1.5 ml

```
                                A-TM3 linker region              TM3
PMCA2Δ298-372  273 - VTAVGVNSQTGIIFTLLGAGGEEEERKKASMHKKEKSVLQGKLTKLAVQIGKAGLVMSAI-TV
PMCA2Δ298-383  273 - VTAVGVNSQTGIIFTLLGAGGEEEE-----------SVLQGKLTKLAVQIGKAGLVMSAI-TV
ACA8           339 - VTGVGVNTEWGLLMASISEDNGEE------------TPLQVRLNGVATFIGSIGLAVAAAVLV
SERCA1a        223 - VATTGVSTEIGKIRDQMAAT------------EQDKTPLQQKLDEFGEQLSKVISLICVAVWL
```

Appendix Figure S5. Sequence alignment of the A-TM3 loop region and the TM3 domain of PMCA2w/aΔ298-372, PMCA2w/aΔ298-383, ACA8 and SERCA1a.

Positively charged residues marked blue, negatively – red. Italics show the acidic lipid binding site of PMCA2. Alignment of full sequences was performed with uniport.org, sequences from which presented fragments were taken have the following entry numbers in Uniprot: Q01814-2 (PMCA2w/a, modified for the alignment, to show both PMCA deletion variants), Q9LF79 (ACA8), and P04191 (SERCA1a).

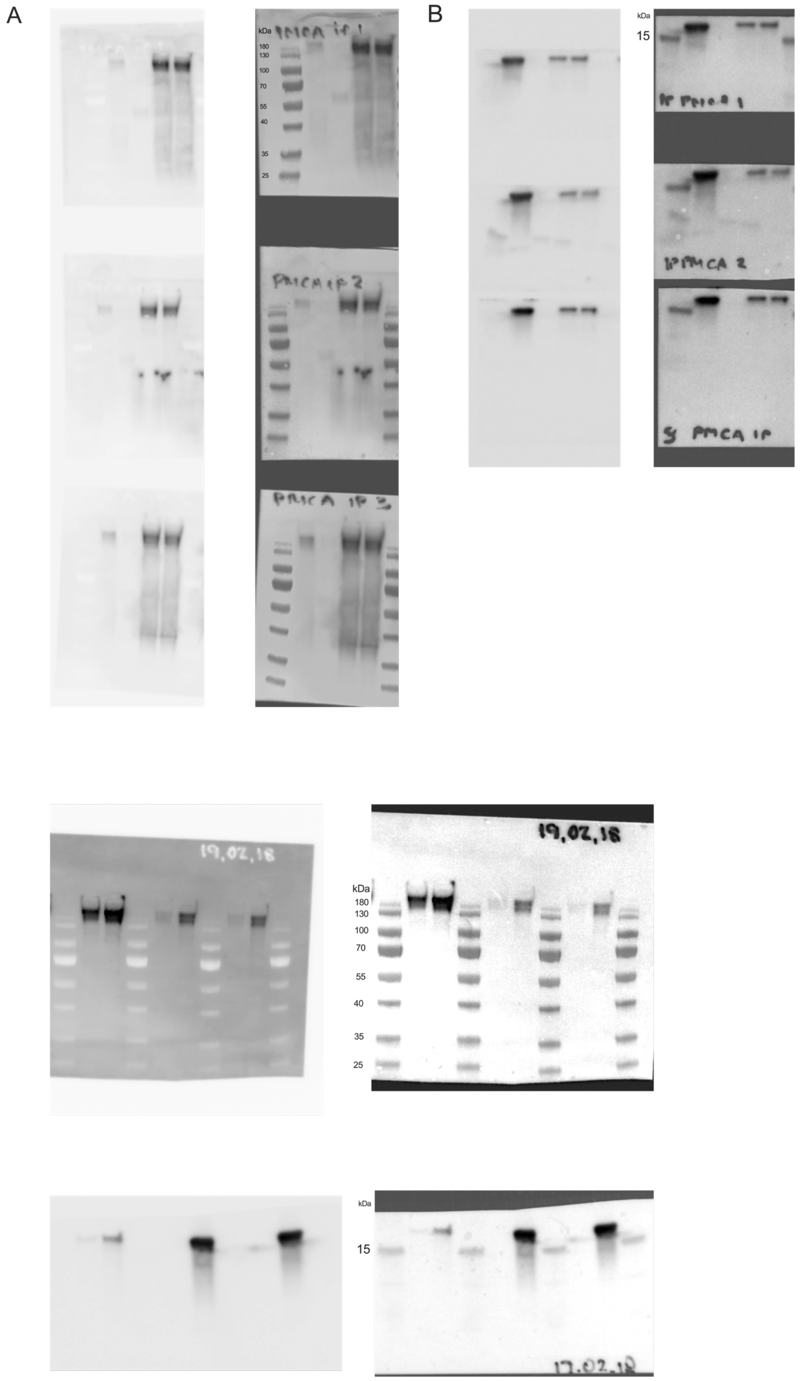

Appendix Figure S6. Raw images of the western blots presented on the Figure 1.

In all sections raw blots are presented on the left and blots merged with image displaying the molecular weight markers are on the right.

A and B. Co-IP assay, where total brain homogenates from aSN-knockout mice were incubated with exogeneous aSN and aSN-binding sepharose (ASY-1). Western blotting performed with anti-pan-PMCA (A) and anti-aSN antibodies (B).

The order of loading of samples is as follows from left: MW marker, input, CO-IP PBS, CO-IP mono, Co-IP oligo, marker

C and D. Co-IP assay, where detergent extracts of C57BL/6 mice were incubated with aSN-binding sepharose (ASY-1) to pull down endogenous aSN. Western blotting performed with anti-pan-PMCA (A) and anti-aSN antibodies (B). The order of samples is as follows: marker, input for IP with nonimmune IgG, input for IP with ASY-1, marker, IP with nonimmune IgG, IP with ASY-1, marker, IP with nonimmune IgG, IP with ASY-1, Marker.

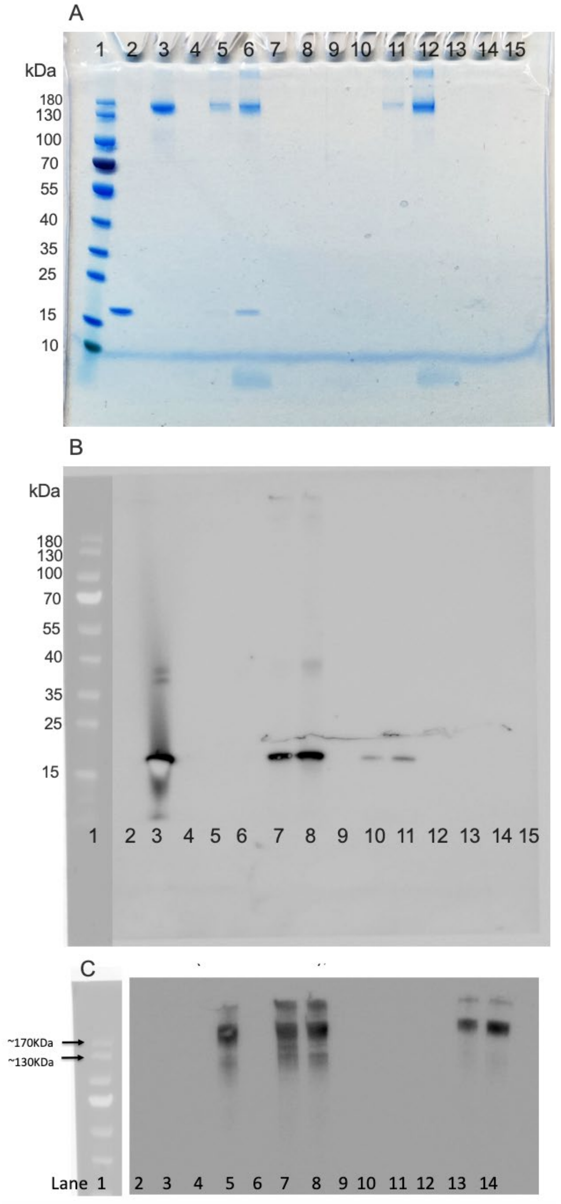

Appendix Figure S7. Pull-down of aSN with PMCA immobilized by Calmodulin-sepharose.

In this experiment pure protein preparations were used.

A. SDS-PAGE analysis, Coomassie staining

Lanes: 1 – MW marker, 2 – pure aSN, 3 – pure PMCA2w/a, 4 – empty lane, Following lanes contain samples incubated with CaM sepharose and eluted with EGTA-buffer: 5 – PMCA+aSN; 6 – PMCA2w/a+aSN+brain lipid extract (BE), 8 – aSN, 9 – aSN+BE, 11 – PMCA2w/a, 12 – PMCAw/a+BE; Lanes 7, 10, 13-15 do not contain any sample

B. Western blotting detection of aSN using anti-ASY-R1 rabbit primary antibodies and anti-rabbit HRP (Dako - P0217) as secondary antibodies

Lanes: Lanes: 1 – MW marker, 2 – pure aSN, 3-4 – empty laners, 5 – pure PMCA2w/a, 4-6 – empty lanes; Following lanes contain samples incubated with CaM sepharose and eluted with EGTA-buffer: 7 – PMCA+aSN; 8 – PMCA2w/a+aSN+brain lipid extract (BE), 10 – aSN, 11 – aSN+BE, 13 – PMCA2w/a, 14 – PMCAw/a+BE; Lanes 9, 13 and 15 do not contain any sample

C. Western blotting detection of PMCA using anti-PMCA (5F10, Abcam, ab2825) mouse primary antibodies and anti-mouse HRP secondary antibodies (Dako – P0260)

This figure shows the exact same membrane as shown on figure B, which was stripped and reprobed to detect PMCA.

Supplementary methods

A model for aSN-dependent calcium regulation

In order to conceptualize the findings of aSN-mediated PMCA activation and its influence on the Ca^{2+} concentration in the presynaptic terminal, we adapted an existing ODE-model published by Erler et al. (Erler, Meyer-Hermann et al., 2004), hereafter named the Erler-model, to take into account the new dependence of PMCA on aSN.

Here we briefly introduce the Erler model, before introducing the changes applied to take into account the aSN-dependence of PMCA.

The Erler-model describes Ca^{2+} - fluxes through PMCA, the H^{+}/Ca^{2+} exchanger NCX and the voltage gated Ca^{2+} channel VGCC and studies calcium dynamics during action potentials in absence and presence of external Ca^{2+} buffers like Ca^{2+} - fluorophores (Erler et al., 2004). Erler et al. made use of the Hodgkin-Huxley model (Hodgkin & Huxley, 1952, Keener & Sneyd, 2009) to describe the membrane potential dynamics during an action potential. The dynamics of the cytosolic Ca^{2+} ion concentration was described by the following differential equation:

$$\frac{d[Ca^{2+}]}{dt} = \frac{G}{z_{Ca}F}(J_{VGCC} - J_{PMCA} - J_{NCX} + J_{leak})\frac{1}{1 + T_{en} + T_{ex}}$$

Where J_{VGCC} denotes the influx of Ca^{2+} through voltage gated Ca^{2+} channels, J_{PMCA} and J_{NCX} denote Ca^{2+} effluxes mediated by PMCA and NCX respectively and J_{leak} denotes a leakage flux of Ca^{2+} ions across the membrane down its electrochemical gradient. The changes in Ca^{2+} concentration are given in $\frac{\mu M}{ms}$.

The term $\frac{G}{z_{Ca}F}$ is a conversion factor from current per unit of time to concentration per unit of time, which includes the geometry factor G in μm^{-1} representing the surface to volume ratio, the ion valence z_{Ca}, and Faraday's constant F in $\frac{As}{mol}$. As the term $\frac{1}{1+T_{en}+T_{ex}}$ is unitless, all fluxes J are defined in units of $\frac{aA}{\mu m^2}$. The term $\frac{1}{1+T_{en}+T_{ex}}$ represents calcium ion buffering through endogenous and exogenous buffers, where $T_{en}(c) = \frac{b_{en}^0 K_{en}}{(K_{en}+[Ca^{2+}])^2}$ and $T_{ex}(c) = \frac{b_{ex}^0 K_{ex}}{(K_{ex}+[Ca^{2+}])^2}$ with $K_{en} = \frac{k_{en}^-}{k_{en}^+}$ as the ratio of the rate constants, while b_{en}^0 and b_{ex}^0 describe the concentration of the total concentration of buffer proteins.

The calcium ion influx through the voltage-gated calcium channels (VGCC) is given by:

$$J_{VGCC} = \rho_V g_V(V) g_{Ca}(V_{ca} - V)$$

Where ρ_V denotes the surface density of VGCCs, g_V denotes the voltage dependent opening probability, g_{Ca} is the open pore conductivity, V is the membrane potential, and V_{Ca} is the reversal potential, described by the Nernst equation:

$$V_{Ca} = \frac{RT}{z_{Ca}F} ln\left(\frac{[Ca^{2+}]_{ext}}{[Ca^{2+}]}\right) - \Delta V_{eff}$$

Where R is the molar gas constant, T is the temperature, $[Ca^{2+}]_{ext}$ is the external calcium ion concentration and ΔV_{eff} is a correction factor for the linear approximation used for the single channel open current $g_{Ca}(V_{ca} - V)$ (Erler et al., 2004). The time dependence of voltage dependent opening probability is modeled by a single exponential approximation:

$$\frac{d}{dt} g_V = (g_\infty(V) - g_V))\frac{1}{\tau}$$

Where the opening probability reaches its asymptotic value $g_\infty(V)$ with time constant τ. The asymptotic value $g_\infty(V)$ can be described by a sigmoidal function:

$$g_\infty(V) = \frac{1}{exp\left((V_h - V)\frac{1}{\kappa}\right) + 1}$$

Where V_h is the half activation voltage and κ is the steepness at the inflection point of $g_\infty(V)$. The calcium ion efflux through PMCA and NCX are modeled as Hill equations:

$$J_{PMCA,Erler} = \rho_p I_p \frac{c^{n_p}}{c^{n_p} + {H_p}^{n_p}} \quad , \quad J_{NCX} = +\rho_x I_x \frac{c^{n_x}}{c^{n_x} + {H_x}^{n_x}} \; .$$

Where ρ_p and ρ_x are the specific surface densities, I_p and I_x are the universal maximum activity rates, H_p and H_x are the half activation concentrations, and n_p and n_x are the Hills coefficients of PMCA and NCX respectively. L is the leakage surface current density, which is determined by the steady state conditions, and ensures that the model can maintain equilibrium when unperturbed.

To account for the aSN concentration dependency of the PMCA activity, the flux through PMCA used by Erler et al. was replaced by another kinetic rate law, which can be derived from the following binding scheme, which allows for description of non-essential activation as seen by aSN. :

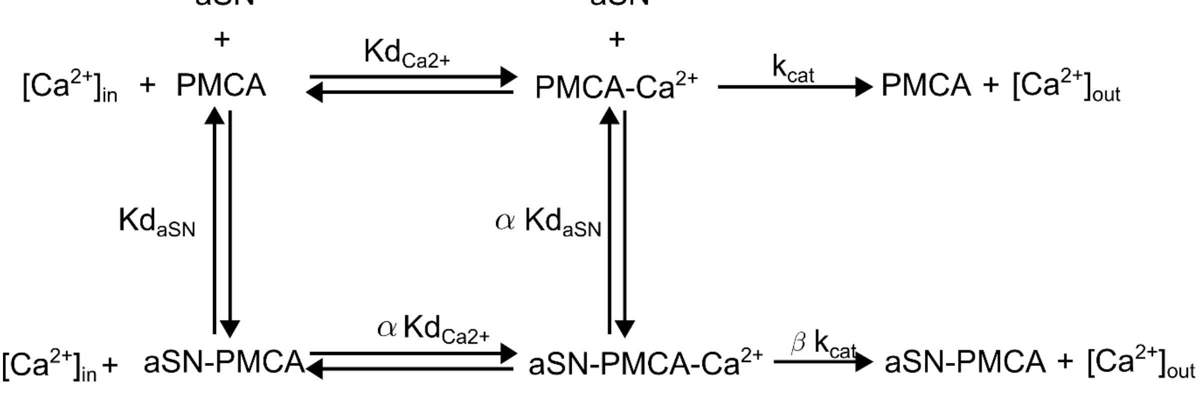

.

Where $K_{d,Ca}$ is the dissociation constant of calcium, $K_{d,aSN}$ is the dissociation constant of aSN, k_{cat} is the catalytic constant of PMCA, α is the reciprocal allosteric coupling constant, and β is the factor by which aSN affects the catalytic constant.

The resulting equation is (Baici, 2015):

$$J_{PMCA}\left(v_{max,PMCA}, \alpha, \beta, K_{d,Ca}, K_{d,aSN}\right) = \frac{v_{max,PMCA}\frac{[Ca^{2+}]}{K_{d,Ca}} + \beta\frac{v_{max,PMCA}[Ca^{2+}][aSN]}{\alpha K_{d,Ca}K_{d,aSN}}}{1 + \frac{[Ca^{2+}]}{K_{d,Ca}} + \frac{[aSN]}{K_{d,aSN}} + \frac{[Ca^{2+}][aSN]}{\alpha K_{d,Ca}K_{d,aSN}}} \quad \textbf{(S1)}$$

$$= v_{max,PMCA}\frac{\frac{[Ca^{2+}]}{K_{d,Ca}} + \beta\frac{[Ca^{2+}][aSN]}{\alpha K_{d,Ca}K_{d,aSN}}}{1 + \frac{[Ca^{2+}]}{K_{d,Ca}} + \frac{[aSN]}{K_{d,aSN}} + \frac{[Ca^{2+}][aSN]}{\alpha K_{d,Ca}K_{d,aSN}}}$$

In order to determine the kinetic parameters, the kinetic for PMCA was fitted to the PMCA1 activity in dependence of [Ca^{2+}] and [aSN] (**Figure 2A** left and **Figure 2B**), using the minimize function from scipy.optimize (VirtanenGommers et al., 2020) and the cost function:

$$\chi^2(v_{max}, \alpha, \beta, K_{d,Ca}, K_{d,aSN}) = \sum_i \left(\frac{y_i - J_{PMCA}(v_{max}, \alpha, \beta, K_{d,Ca}, K_{d,aSN})}{\sigma_i}\right)^2$$

With σ_i being the standard error of the mean corresponding to the data point y_i.

The optimal parameter set was given by:

$$v_{max,PMCA(fit)} = 1.93\,\frac{nmol\ Pi}{\mu g\ PMCA\ min}$$

$$\alpha = 0.29$$

$$\beta = 4.31$$

$$K_{d,aSN} = 5.5\ \mu M$$

$$K_{d,Ca} = 0.29\ \mu M$$

The resulting fit is shown in **Appendix Figure S8**. Note that the data points for high calcium concentrations were excluded from the fitting process.

Calculation of dependent parameters

The parameter $v_{max,PMCA(fit)}$ obtained by the fitting process is given in $\frac{nmol\ Pi}{\mu g\ PMCA\ min}$ and can not be translated directly into the units required by the model, i.e. $\frac{aA}{\mu m^2}$.

We therefore assumed that the flux mediated by PMCA in our updated version should equal the flux through the kinetic used by Erler et al. at the steady state Ca^{2+} concentration of 0.1 µM and an aSN concentration of 40 µM as measured by Wilhelm et al. (Wilhelm, Mandad et al., 2014), assuming that in the data used by Erler et al. also around 40 µM aSN were present. Thus, at $[Ca^{2+}]_{eq} = 0.1\ \mu M$ and $[aSN]_{eq} = 40\ \mu M$ the PMCA mediated fluxes should be equal, i.e.:

$$\rho_p I_p \frac{{[Ca^{2+}]_{eq}}^{n_p}}{{[Ca^{2+}]_{eq}}^{n_p} + {H_p}^{n_p}} \equiv J([Ca^{2+}]_{eq}, [aSN]_{eq}) =$$

$$v_{max,PMCA} \frac{\frac{[Ca^{2+}]_{eq}}{K_{d,Ca}} + \beta \frac{[Ca^{2+}]_{eq}[aSN]_{eq}}{\alpha K_{d,Ca} K_{d,aSN}}}{1 + \frac{[Ca^{2+}]_{eq}}{K_{d,Ca}} + \frac{[aSN]_{eq}}{K_{d,aSN}} + \frac{[Ca^{2+}]_{eq}[aSN]_{eq}}{\alpha K_{d,Ca} K_{d,aSN}}}$$

And hence:

$$v_{max,PMCA} = \rho_p I_p \frac{{[Ca^{2+}]_{eq}}^{n_p}}{{[Ca^{2+}]_{eq}}^{n_p} + {H_p}^{n_p}} \cdot \frac{1 + \frac{[Ca^{2+}]_{eq}}{K_{d,Ca}} + \frac{[aSN]_{eq}}{K_{d,aSN}} + \frac{[Ca^{2+}]_{eq}[aSN]_{eq}}{\alpha K_{d,Ca} K_{d,aSN}}}{\frac{[Ca^{2+}]_{eq}}{K_{d,Ca}} + \beta \frac{[Ca^{2+}]_{eq}[aSN]_{eq}}{\alpha K_{d,Ca} K_{d,aSN}}} .$$

The leakage flux $J_{leak,eq}$ is modeled as GHK-flux equation (Keener & Sneyd, 2009), which depends on the permeability of the membrane for Ca^{2+} (P_{Ca}), which was calculated using the steady state assumption:

$$J_{PMCA,eq} + J_{NCX,eq} + J_{VGCC,eq} + J_{leak,eq} \equiv 0$$

With $J_{PMCA,eq}$, $J_{NCX,eq}$ and $J_{VGCC,eq}$ being the fluxes mediated by PMCA, NCX and VGCC at equilibrium and

$$J_{leak,eq} = P_{Ca} \frac{z_{Ca}^2 V_{eq} F^2}{RT} \cdot \frac{[Ca^{2+}]_{eq} - [Ca^{2+}]_{ext} \cdot exp(-\frac{z_{Ca}^2 V_{eq} F}{RT})}{1 - exp(-\frac{z_{Ca}^2 V_{eq} F}{RT})} ,$$

It follows:

$$P_{Ca} = (J_{PMCA,eq} + J_{NCX,eq} + J_{VGCC,eq}) \cdot \frac{RT}{z_{Ca}^2 V_{eq} F^2} \cdot \frac{1 - exp(-\frac{z_{Ca}^2 V_{eq} F}{RT})}{[Ca^{2+}]_{eq} - [Ca^{2+}]_{exz} \cdot exp(-\frac{z_{Ca}^2 V_{eq} F}{RT})}$$

And finally:

$$J_{leak} = P_{Ca} \frac{z_{Ca}^2 V F^2}{RT} \cdot \frac{[Ca^{2+}] - [Ca^{2+}]_{ext} \cdot exp(-\frac{z_{Ca}^2 V F}{RT})}{1 - exp(-\frac{z_{Ca}^2 V F}{RT})} .$$

Complete ODE System

Finally, the entire system of ODEs is given by:

$$\frac{dm}{dt} = \alpha_m (1 - m) - \beta_m m$$

$$\frac{dn}{dt} = \alpha_n (1 - n) - \beta_n n$$

$$\frac{dh}{dt} = \alpha_h (1 - h) - \beta_h h$$

$$\frac{dV}{dt} = (-g_k\, n^4\,(V - V_k) - g_{Na}\, m^3\, h\,(V - V_{Na}) - g_{leak}\,(V - V_{leak}) + I_{app})\,/\,C_m$$

$$\frac{dg_V}{dt} = (g_\infty(V) - g_V))\frac{1}{\tau}$$

$$\frac{d[Ca^{2+}]}{dt} = \frac{G}{z_{Ca}F}(J_{VGCC} - J_{PMCA} - J_{NCX} + J_{leak})\frac{1}{1+T_{en}+T_{ex}},$$

with the first four equation describing the Hodgkin-Huxley model as described by Keener and Sneyd (Keener & Sneyd, 2009), the fifth describing the change of the open probability of the voltage-gated Ca^{2+} - channels, the sixth describing the change in the cytosolic Ca^{2+} concentration.
The initial values and parameters are shown in **Appendix Table S1**.

Appendix Table S1. Initial values and parameters for the calcium regulation model.

Parameter	Value	Unit	Reference
m_0	0.05296		(Keener & Sneyd, 2009)
n_0	0.317732		(Keener & Sneyd, 2009)
h_0	0.595995		(Keener & Sneyd, 2009)
V_0	-65	mV	(Keener & Sneyd, 2009)
$g_{v,0}$	$6.236 \cdot 10^{-5}$		steady state assumption
$[Ca^{2+}]_0$	0.1	µM	(Erler et al., 2004)
G	6	µm	(Erler et al., 2004)
z_{Ca}	2		
F	96485	$\frac{As}{mol}$	
R	8.314	$\frac{J}{mol \cdot K}$	
T	310	K	
$[Ca^{2+}]_{ext}$	1500	µM	(Erler et al., 2004)
I_p	$2.7 * 10^{-21}$	C/ms	(Erler et al., 2004)
n_p	2		(Erler et al., 2004)
H_p	0.09	µM	(Erler et al., 2004)

ρ_p	9200	μm^{-2}	(Erler et al., 2004)
I_x	$4.8 * 10^{-19}$	C/ms	(Erler et al., 2004)
n_x	1		(Erler et al., 2004)
H_x	1.8	μM	(Erler et al., 2004)
ρ_x	$0.033 \cdot \rho_p$	μm^{-2}	(Erler et al., 2004)
g_{ca}	$14 \cdot 10^3$	fS	(Erler et al., 2004)
τ	1	ms	(Erler et al., 2004)
κ	6.3	mV	(Erler et al., 2004)
ΔV_{eff}	47	mV	(Erler et al., 2004)
V_h	-4	mV	(Erler et al., 2004)
ρ_v	3.1	μm^{-2}	(Erler et al., 2004)
K_{en}	0.5	μM	(Erler et al., 2004)
b_{en}^0	120	μM	(Erler et al., 2004)
K_{ex}	6	μM	(Erler et al., 2004)
b_{ex}^0	0	μM	(Erler et al., 2004)
α	0.29		**Appendix Figure S8**
β	4.31		**Appendix Figure S8**
$K_{d,aSN}$	5.5	μM	**Appendix Figure S8**
$K_{d,Ca}$	0.29	μM	**Appendix Figure S8**

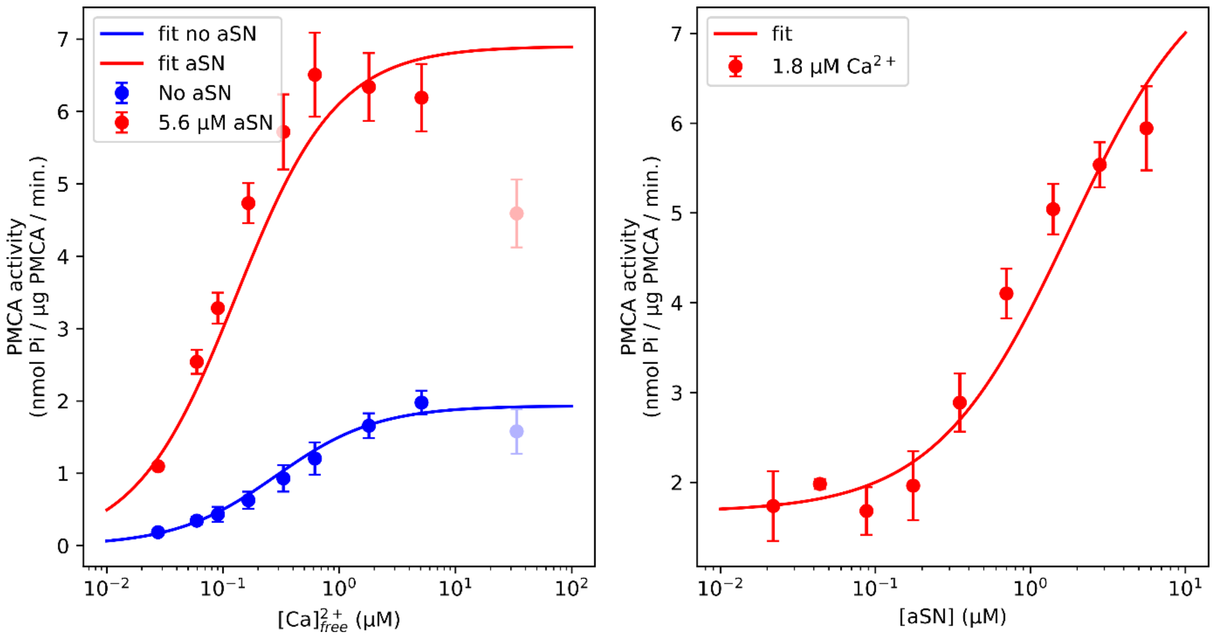

Appendix Figure S8. Monomeric alpha-synuclein acts as a non-essential activator of PMCA. The kinetic equation derived from the assumption of non-essential activation of PMCA by aSN was fitted to the Ca^{2+} and aSN dependence reported in **Figure 2**. The non-saturated data points (faded colors) were excluded from the fitting process.

References

Baici A (2015) Kinetics of Enzyme-Modifier Interactions: Selected Topics in the Theory and Diagnosis of Inhibition and Activation Mechanisms. *Vienna: Springer*

Erler F, Meyer-Hermann M, Soff G (2004) A quantitative model for presynaptic free Ca2+ dynamics during different stimulation protocols. *Neurocomputing* 61: 169-191

Hodgkin AL, Huxley AF (1952) A quantitative description of membrane current and its application to conduction and excitation in nerve. 117: 500-544

Keener J, Sneyd J (2009) Mathematical Physiology I: Cellular Physiology. *Springer, New York*

Virtanen P, Gommers R, Oliphant TE, Haberland M, Reddy T, Cournapeau D, Burovski E, Peterson P, Weckesser W, Bright J, van der Walt SJ, Brett M, Wilson J, Millman KJ, Mayorov N, Nelson ARJ, Jones E, Kern R, Larson E, Carey CJ et al. (2020) SciPy 1.0: fundamental algorithms for scientific computing in Python. *Nature Methods* 17: 261-272

Wilhelm BG, Mandad S, Truckenbrodt S, Kröhnert K, Schäfer C, Rammner B, Koo SJ, Claßen GA, Krauss M, Haucke V, Urlaub H, Rizzoli SO (2014) Composition of isolated synaptic boutons reveals the amounts of vesicle trafficking proteins. 344: 1023-1028

Computer Code – Transcriptomics analysis

The original code written in R for the purpose of the analysis of transcriptomics data.

The code relevant for the analysis displayed on the Figure 3A:

```
install.packages("tidyverse")
install.packages("BSDA")
library("tidyverse")

library("BSDA")

# Data import
PMCA3 <- read_tsv("PMCA3.csv")

PMCA1 <- read_tsv("PMCA1.csv")

PMCA4 <- read_tsv("PMCA4.csv")

asn <- read_tsv("alphasynuclein.csv")

# function grouping data by subgroup and calculating mean CRPKM within the
subgroup

CRPKMs_mean <- function(data) {
  data %>%
  group_by(Subgroup) %>%
  summarise(
    Order = mean(Order),
    CRPKMs = mean(CRPKMs),
    Group = Group)
}

# Applying the CRPKMs_mean function to the PMCA and aSN data

PMCA1_CRPKMs_mean <- CRPKMs_mean(PMCA1)

PMCA3_CRPKMs_mean <- CRPKMs_mean(PMCA3)

PMCA4_CRPKMs_mean <- CRPKMs_mean(PMCA4)

asn_CRPKMs_mean <- CRPKMs_mean(asn)

# Data import
exon_PMCA1 <- read_tsv("exon_incorporation_PMCA1.csv")

exon_PMCA3 <- read_tsv("exon_incorporation_PMCA3.csv")

exon_PMCA4 <- read_tsv("exon_incorporation_PMCA4.csv")

# function grouping data by subgroup and calculating mean exon incorporati
on value within the subgroup

exon_mean <- function(data) {
  data %>%
  group_by(EventID, Subgroup) %>%
  summarise(
```

```
    Order = mean(Order),
    Value = mean(Value, na.rm = TRUE),
    Group = Group)
}

PMCA1_exon_mean <- exon_mean(exon_PMCA1)

PMCA3_exon_mean <- exon_mean(exon_PMCA3)

PMCA4_exon_mean <- exon_mean(exon_PMCA4)

#filtering exon incorporation data for the relevant tissues - tissues with
actual PMCA expression

exsp_exon <- function(data1, data2) {
  exsp_tissue <- filter(data1, CRPKMs > 1.5)
    Tissue <- c(exsp_tissue$Subgroup)
    subset(data2, Subgroup %in% Tissue)
}

PMCA1_exsp_exon <- exsp_exon(PMCA1_CRPKMs_mean, PMCA1_exon_mean)
PMCA3_exsp_exon <- exsp_exon(PMCA3_CRPKMs_mean, PMCA3_exon_mean)
PMCA4_exsp_exon <- exsp_exon(PMCA4_CRPKMs_mean, PMCA4_exon_mean)


# filtering for relevant event IDs - events leading to the a variant at sp
lice site C variants:

#PMCA1c is achieved with HsaALTD0000577-1/2  (green)
#PMCA1a is achieved with HsaALTD0000577-2/2 (blue)
#PMCA1b is achieved without exon HsaEX0006882
#PMCA1a is achieved with exon HsaEX0006882 (yellow)
PMCA1_exsp_exon <- filter(PMCA1_exsp_exon, EventID == "HsaEX0006882")



#HsaEX0006895 with incorporation PMCA3x without PMCA3z, HsaEX0006897 with
then PMCA3a and without then PMCA3b
PMCA3_exsp_exon <- filter(PMCA3_exsp_exon,  EventID == "HsaEX0006897")

#PMCA4z is achieved without the HsaEX0006899 exon #PMCA4x is achieved with
the HsaEX0006899 exon #PMCA4a is achieved with the HsaEX0006904 exon #PMCA
4b is achieved without the HsaEX0006904 exon
PMCA4_exsp_exon <- filter(PMCA4_exsp_exon, EventID == "HsaEX0006904")

# function for calculation of mean incorporation of a specific splicing ev
ent for tissues within a designated interval of aSN expression

Event_mean_int_PMCA <- function(PMCA_data){

interval <- seq(0, 190, by=10)
exsp_asn <- list()
Event_mean_PMCA <- list()
Event_mean_all_tissues <- list()
Event_mean_brain <- list()
```

```
Event_mean_PMCA_tissue <- list()

i <- 1
while (i < length(interval)) {


  exsp_asn[[paste(interval[[i+1]])]] <- filter(asn_CRPKMs_mean, CRPKMs >=
interval[[i]] & CRPKMs < interval[[i+1]])
  Event_mean_PMCA[[paste(interval[[i+1]])]] <- subset(PMCA_data, Subgroup
%in% exsp_asn[[i]]$Subgroup)

  Event_mean_all_tissues[[i]] <- Event_mean_PMCA[[paste(interval[[i+1]])]]
%>%
    summarise(n_group = n())

  Event_mean_brain[[i]] <- Event_mean_PMCA[[paste(interval[[i+1]])]] %>%
  filter(Group == "Neural_crest" | Group == "Neural" | Group == "NPC" | Gr
oup == "Glia" | Group == "EmbrBrain") %>%
    summarise(n_group = n())

Event_mean_PMCA_tissue[[paste(interval[[i+1]])]] <- Event_mean_PMCA[[paste
(interval[[i+1]])]] %>%
  filter(Value >= 0) %>%
  group_by(EventID) %>%
  summarise(mean_PSI = mean(Value),
            sd_PSI = sd(Value),
            n_tissues = n()) %>%
  add_column(percent_brain = sum(Event_mean_brain[[i]]$n_group)/sum(Event_
mean_all_tissues[[i]]$n_group)*100) %>%
  add_column(asn_exp_interval = interval[[i+1]])


  i <- i + 1

}

Event_mean_PMCA_tissue <- data.table::rbindlist(Event_mean_PMCA_tissue)

}

Event_mean_int_PMCA1 <- Event_mean_int_PMCA(PMCA1_exsp_exon)

Event_mean_int_PMCA3 <- Event_mean_int_PMCA(PMCA3_exsp_exon)

Event_mean_int_PMCA4 <- Event_mean_int_PMCA(PMCA4_exsp_exon)


#Intervals for plotting mean incorporation of exons as a function of the a
SN expression interval

expression_interval <- c("0-10", "10-20", "20-30", "30-40", "40-50", "50-6
0", "60-70", "70-80", "80-90", "90-100", "100-110", "110-120", "120-130",
"130-140", "140-150", "150-160", "160-170", "170-180", "180-190")
```

```
PMCA1_a <- Event_mean_int_PMCA1
PMCA3_a <- Event_mean_int_PMCA3
PMCA4_a <- Event_mean_int_PMCA4

# The tissues within aSN intervals 110-120 and 130-140. These are outliers

asn_120 <- filter(asn_CRPKMs_mean, CRPKMs >= 110 & CRPKMs < 120)

asn_140 <- filter(asn_CRPKMs_mean, CRPKMs >= 130 & CRPKMs < 140)

labels <- tibble(x = c(95, 165),
                 y = c(5,0),
                 label = c(asn_120$Subgroup[1], asn_140$Subgroup[1]))

# Plotting mean incorporation of events leading to variant a at different
aSN intervals

ggplot() +
  geom_point(data = PMCA1_a, mapping = aes(x = asn_exp_interval, y = mean_
PSI, color = EventID), size = 2.5) +
  geom_point(data = PMCA4_a, mapping = aes(x = asn_exp_interval, y = mean_
PSI, color = EventID), size = 2.5) +
  geom_point(data = PMCA3_a, mapping = aes(x = asn_exp_interval, y = mean_
PSI, color = EventID), size = 2.5) +
  theme_bw() +
  theme(legend.title=element_blank()) +
  scale_color_manual(labels = c("PMCA1 \n HsaEX0006882", "PMCA3 \n HsaEX00
06897", "PMCA4 \n HsaEX0006904"), values=c("#FFCC33","#9900CC", "#0000FF")
) +
  labs(x = "aSN expression interval (CRPKMs)", y = "mean PSI \n PMCA exons
") +
  theme(axis.text.x = element_text(angle = 90, hjust = 1, vjust = 0.5)) +
  theme(legend.position="bottom") +
  scale_x_continuous(breaks = seq(10, 190, by = 10), labels = expression_i
nterval) +
  geom_label(data = labels, mapping = aes(x=x, y=y, label=label), size = 3
)+
  ggsave(width = 5, height = 4, filename = "mean_PSI_interval_PMCA_a_versi
on.png")

# Plotting with standard deviations

  ggplot() +
  geom_point(data = PMCA1_a, mapping = aes(x = asn_exp_interval, y = mean_
PSI, color = EventID), size = 2.5) +
  geom_point(data = PMCA4_a, mapping = aes(x = asn_exp_interval, y = mean_
PSI, color = EventID), size = 2.5) +
  geom_point(data = PMCA3_a, mapping = aes(x = asn_exp_interval, y = mean_
PSI, color = EventID), size = 2.5) +
  geom_errorbar(data = PMCA1_a, aes(x=asn_exp_interval, ymin=mean_PSI-sd_P
SI, ymax=mean_PSI+sd_PSI, width = 5))+
   geom_errorbar(data = PMCA3_a, aes(x=asn_exp_interval, ymin=mean_PSI-sd_
PSI, ymax=mean_PSI+sd_PSI, width = 5))+
   geom_errorbar(data = PMCA4_a, aes(x=asn_exp_interval, ymin=mean_PSI-sd_
```

```
PSI, ymax=mean_PSI+sd_PSI, width = 5))+
  theme_bw() +
  theme(axis.text.x = element_text(angle = 90, hjust = 1, vjust = 0.5)) +
  theme(legend.title=element_blank()) +
  scale_color_manual(labels = c("PMCA1 \n HsaEX0006882", "PMCA3 \n HsaEX00
06897", "PMCA4 \n HsaEX0006904"), values=c("#FFCC33","#9900CC", "#0000FF")
) +
  labs(x = "aSN expression interval (CRPKMs)", y = "mean PSI \n PMCA exons
") +
  theme(axis.text.x = element_text(angle = 90, hjust = 1, vjust = 0.5)) +
  theme(legend.position="bottom") +
    scale_x_continuous(breaks = seq(10, 190, by = 10), labels = expression
_interval) +
  geom_label(data = labels, mapping = aes(x=x, y=y, label=label), size = 3
)+
    ggsave(width = 5, height = 4, filename = "mean_PSI_interval_PMCA_a_ver
sion_error.png")

#t-test - calculate if there is a stastically significant difference betwe
en the mean PSI for tissues with aSN expression below 50 CRPKMs and above
50 CRPKMs.

interval_means <- function(PMCA_data, interval1, interval2) {
exsp_asn <- filter(asn_CRPKMs_mean, CRPKMs >= interval1 & CRPKMs < interva
l2)
  Event_mean_PMCA <- subset(PMCA_data, Subgroup %in% exsp_asn$Subgroup) %>
%
    group_by(EventID) %>%
    summarise(mean_PSI = mean(Value),
              sd_PSI = sd(Value),
              n_tissues = n())
}

#- For PMCA4
low_data_means <- interval_means(PMCA4_exsp_exon, 0, 50)

high_data_means <- interval_means(PMCA4_exsp_exon, 50, 190)


tsum.test(mean.x = (low_data_means[[1,2]]), s.x = (low_data_means[[1,3]]),
n.x = (low_data_means[[1,4]]), mean.y = (high_data_means[[1,2]]), s.y = (h
igh_data_means[[1,3]]), n.y = (high_data_means[[1,4]]))

##
##  Welch Modified Two-Sample t-Test
##
## data:  Summarized x and y
## t = -2.6275, df = 10.859, p-value = 0.02374
## alternative hypothesis: true difference in means is not equal to 0
## 95 percent confidence interval:
##  -53.460998  -4.680534
## sample estimates:
## mean of x mean of y
##  16.24105  45.31182
```

```r
#for PMCA1
low_data_means_1 <- interval_means(PMCA1_exsp_exon, 0, 50)

high_data_means_1 <- interval_means(PMCA1_exsp_exon, 50, 190)

tsum.test(mean.x = (low_data_means_1[[1,2]]), s.x = (low_data_means_1[[1,3
]]), n.x = (low_data_means_1[[1,4]]), mean.y = (high_data_means_1[[1,2]]),
s.y = (high_data_means_1[[1,3]]), n.y = (high_data_means_1[[1,4]]))

##
##  Welch Modified Two-Sample t-Test
##
## data:  Summarized x and y
## t = -3.3017, df = 10.4, p-value = 0.007589
## alternative hypothesis: true difference in means is not equal to 0
## 95 percent confidence interval:
##  -73.90402 -14.53230
## sample estimates:
## mean of x mean of y
##  7.147292 51.365455

## Adjusting tables for a more convenient look for publication

PMCA1a <- PMCA1_a %>% mutate(across(is.numeric, ~ round(., 2)))

PMCA3a <- PMCA3_a %>% mutate(across(is.numeric, ~ round(., 2)))
PMCA4a <- PMCA4_a %>% mutate(across(is.numeric, ~ round(., 2)))

aSN_exp_int <- c("0-10", "10-20", "20-30", "30-40", "40-50", "50-60", "80-
90", "110-120", "120-130", "130-140", "180-190")

PMCA1a_adjusted  <- PMCA1a  %>% add_column(aSN_exp_int)
PMCA1a_adjusted[,c(7,2,3,4,5)]

PMCA4a_adjusted  <- PMCA4a  %>% add_column(aSN_exp_int)
PMCA4a_adjusted[,c(7,2,3,4,5)]

aSN_exp_int <- c("0-10", "10-20", "40-50", "50-60", "80-90", "120-130", "1
80-190")

PMCA3a_adjusted  <- PMCA3a  %>% add_column(aSN_exp_int)
PMCA3a_adjusted[,c(7,2,3,4,5)]
```

The code relevant for the analysis displayed on the Figure 3B:

```
library(tidyverse)

library(BSDA)

PMCA2 <- read_tsv("PMCA2.csv")

PMCA2_CRPKMs_mean <- PMCA2 %>%
  group_by(Subgroup) %>%
  summarise(
    Order = mean(Order),
    CRPKMs = mean(CRPKMs))

asn <- read_tsv("alphasynuclein.csv")

asn_CRPKMs_mean <- asn %>%
  group_by(Subgroup) %>%
  summarise(
    Order = mean(Order),
    CRPKMs = mean(CRPKMs))

exon_PMCA2 <- read_tsv("exon_incorporation_PMCA2.csv")

exon_PMCA2

#filtering for the relevant tissues - tissues with actual PMCA2 expression
exsp_PMCA2 <- filter(PMCA2_CRPKMs_mean, CRPKMs > 1.5)
exsp_PMCA2

#taking the expression levels into account:
PMCA2_exon_tissues <- c(exsp_PMCA2$Subgroup)
PMCA2_exon_tissues

PMCA2_exon_mean <- exon_PMCA2 %>%
  group_by(EventID, Subgroup) %>%
  summarise(
    Order = mean(Order),
    Value = mean(Value, na.rm = TRUE),
    Group = Group)

PMCA2_exon_mean

PMCA2_exon_mean <- filter(PMCA2_exon_mean, EventID == "HsaEX0006891" |  Ev
entID == "HsaEX0006892" |  EventID == "HsaEX0006893" )




ggplot(data = PMCA2_exon_mean) +
  geom_point(mapping = aes(reorder(Subgroup, Order), y = Value, color = PM
CA2_exon_mean$EventID)) +
  theme_bw() +
  theme(axis.text.x = element_text(angle = 90, hjust = 1, vjust = 0.5)) +
```

```
  theme(legend.title=element_blank()) +
  scale_color_manual(values=c("#FF6600", "#FFCC33", "#000066"))
```

```
PMCA2_exsp_exon <- subset(PMCA2_exon_mean, Subgroup %in% PMCA2_exon_tissue
s)
PMCA2_exsp_exon

ggplot(data = PMCA2_exsp_exon) +
  geom_jitter(mapping = aes(reorder(Subgroup, Order), y = Value, color = P
MCA2_exsp_exon$EventID), width = 0.1, height = 0.1) +
  theme_bw() +
  theme(axis.text.x = element_text(angle = 90, hjust = 1, vjust = 0.5)) +
  theme(legend.title=element_blank()) +
  scale_color_manual(values=c("#FF6600", "#FFCC33", "#000066")) +
  xlab('') + ylab('PSI') +
  ggsave("exons.png")

#------------------------------- The mean incorporation values for all tis
sues --------------------------###

exsp_asn <- filter(asn_CRPKMs_mean, CRPKMs > 0)
Event_mean_PMCA2 <- subset(PMCA2_exsp_exon, Subgroup %in% exsp_asn$Subgrou
p) %>%
group_by(EventID) %>%
summarise(mean_PSI = mean(Value),
            sd_PSI = sd(Value))

Event_mean_PMCA2

expression_interval <- c("0-10", "10-20", "20-30", "30-40", "40-50", "50-6
0", "60-70", "70-80", "80-90", "90-100", "100-110", "110-120", "120-130",
"130-140", "140-150", "150-160", "160-170", "170-180", "180-190")


# function for calculation of mean incorporation of a specific splicing ev
ent for tissues within a designated interval of aSN expression

Event_mean_int_PMCA <- function(PMCA_data){

interval <- seq(0, 190, by=10)
exsp_asn <- list()
Event_mean_PMCA <- list()
Event_mean_all_tissues <- list()
Event_mean_brain <- list()
Event_mean_PMCA_tissue <- list()

i <- 1
while (i < length(interval)) {


  exsp_asn[[paste(interval[[i+1]])]] <- filter(asn_CRPKMs_mean, CRPKMs >=
interval[[i]] & CRPKMs < interval[[i+1]])
  Event_mean_PMCA[[paste(interval[[i+1]])]] <- subset(PMCA_data, Subgroup
```

```
%in% exsp_asn[[i]]$Subgroup)

  Event_mean_all_tissues[[i]] <- Event_mean_PMCA[[paste(interval[[i+1]])]]
%>%
    summarise(n_group = n())

  Event_mean_brain[[i]] <- Event_mean_PMCA[[paste(interval[[i+1]])]] %>%
  filter(Group == "Neural_crest" | Group == "Neural" | Group == "NPC" | Gr
oup == "Glia" | Group == "EmbrBrain") %>%
    summarise(n_group = n())

Event_mean_PMCA_tissue[[paste(interval[[i+1]])]] <- Event_mean_PMCA[[paste
(interval[[i+1]])]] %>%
  filter(Value >= 0) %>%
  group_by(EventID) %>%
  summarise(mean_PSI = mean(Value),
            sd_PSI = sd(Value),
            n_tissues = n()) %>%
  add_column(percent_brain = sum(Event_mean_brain[[i]]$n_group)/sum(Event_
mean_all_tissues[[i]]$n_group)*100) %>%
  add_column(asn_exp_interval = interval[[i+1]])


  i <- i + 1

}

Event_mean_PMCA_tissue <- data.table::rbindlist(Event_mean_PMCA_tissue)

}

Event_mean_int_PMCA2 <- Event_mean_int_PMCA(PMCA2_exsp_exon)
Event_mean_int_PMCA2

Event_mean_int_PMCA2 %>%
  ggplot() +
  geom_point(mapping = aes(x = asn_exp_interval, y = mean_PSI, color = Eve
ntID), size = 2.5) +
  geom_errorbar(aes(x=asn_exp_interval, ymin=mean_PSI-sd_PSI, ymax=mean_PS
I+sd_PSI, width = 5))+
  theme_bw() +
  #theme(axis.text.x = element_text(angle = 90, hjust = 1, vjust = 0.5)) +
  theme(legend.title=element_blank()) +
  scale_color_manual(values=c("#FF6600", "#FFCC33", "#000066")) +
  labs(x = "aSN expression interval (CRPKMs)", y = "mean PSI")
```

```
theme(axis.text.x = element_text(angle = 90, hjust = 1, vjust = 0.5))

Event_mean_int_PMCA2 %>%
  ggplot() +
  geom_point(mapping = aes(x = asn_exp_interval, y = mean_PSI, color = Eve
ntID), size = 2.5) +
  #geom_errorbar(aes(x=asn_exp_interval, ymin=mean_PSI-sd_PSI, ymax=mean_P
```

```
SI+sd_PSI), width=0.25)+
  theme_bw() +
  #theme(axis.text.x = element_text(angle = 90, hjust = 1, vjust = 0.5)) +
  theme(legend.title=element_blank()) +
  scale_color_manual(values=c("#FF6600", "#FFCC33", "#000066")) +
  labs(x = "aSN expression interval (CRPKMs)", y = "mean PSI \n PMCA2 exon
s") +
  theme(axis.text.x = element_text(angle = 90, hjust = 1, vjust = 0.5)) +
  scale_x_continuous(breaks = seq(10, 190, by = 10), labels = expression_i
nterval)+
  theme(legend.position="bottom") +

  ggsave(width = 5, height = 4, filename = "mean_PSI_interval_PMCA2_2.png"
)
```

```
#t-test - calculate if there is a stastically significant difference betwe
en the mean PSI for tissues with aSN expression below 50 CRPKMs and above
50 CRPKMs.

interval_means <- function(PMCA_data, interval1, interval2) {
exsp_asn <- filter(asn_CRPKMs_mean, CRPKMs >= interval1 & CRPKMs < interva
l2)
  Event_mean_PMCA <- subset(PMCA_data, Subgroup %in% exsp_asn$Subgroup) %>
%
    group_by(EventID) %>%
    summarise(mean_PSI = mean(Value),
              sd_PSI = sd(Value),
              n_tissues = n())
}

# For PMCA2
low_data_means <- interval_means(PMCA2_exsp_exon, 0, 50)

high_data_means <- interval_means(PMCA2_exsp_exon, 50, 190)

# low_data_means
# high_data_means

tsum.test(mean.x = (low_data_means[[1,2]]), s.x = (low_data_means[[1,3]]),
n.x = (low_data_means[[1,4]]), mean.y = (high_data_means[[1,2]]), s.y = (h
igh_data_means[[1,3]]), n.y = (high_data_means[[1,4]]))

##
##  Welch Modified Two-Sample t-Test
##
## data:  Summarized x and y
## t = 4.9465, df = 18.681, p-value = 9.394e-05
## alternative hypothesis: true difference in means is not equal to 0
## 95 percent confidence interval:
##  22.96939 56.73360
## sample estimates:
```

```
## mean of x mean of y
## 42.673158  2.821667

## Adjusting tables for a more convenient look for publication

PMCA2exons <- Event_mean_int_PMCA2 %>% mutate(across(is.numeric, ~ round(.
, 2)))

PMCA2exons

aSN_exp_int <- c("0-10", "10-20", "20-30", "30-40", "40-50", "50-60", "80-
90", "120-130", "180-190")

PMCA2_adjusted_HsaEX0006891  <- PMCA2exons  %>%
  filter(EventID == "HsaEX0006891") %>%
  add_column(aSN_exp_int)

PMCA2_adjusted_HsaEX0006891[,c(7,2,3,4,5,1)]

PMCA2_adjusted_HsaEX0006892  <- PMCA2exons  %>%
  filter(EventID == "HsaEX0006892") %>%
  add_column(aSN_exp_int)

PMCA2_adjusted_HsaEX0006892[,c(7,2,3,4,5,1)]

PMCA2_adjusted_HsaEX0006893  <- PMCA2exons  %>%
  filter(EventID == "HsaEX0006893") %>%
  add_column(aSN_exp_int)

PMCA2_adjusted_HsaEX0006893[,c(7,2,3,4,5,1)]
```
