# Supplementary material for: Monomeric α‐synuclein activates the plasma membrane calcium pump
Source: EMBO J. 2023 Nov 2;42(23):e111122. doi: 10.15252/embj.2022111122 (PMC10690453; doi:10.15252/embj.2022111122)
Supplement: Supplementary file 5 [file EMBJ-42-e111122-s002.pdf]

THE EMBO JOURNAL

Monomeric α-Synuclein activates the Plasma Membrane Calcium Pump

Antoni Kowalski, Cristine Betzer, Sigrid Larsen, Emil Gregersen, Estella Newcombe, Montana Bermejo, Annette Langkilde, Birthe Kragelund, Poul Henning JENSEN, Poul Nissen, Shweta Jain, Robert Edwards, Lene Nejsum, Christina Ernstsen, Viktor Bendtsen, Jorin Diemer, Edda Klipp, and Alicia Bou
DOI: 10.15252/embj.2022111122

Corresponding author(s): Poul Nissen (pn@mbg.au.dk) , Poul Henning JENSEN (phj@biomed.au.dk), Antoni Kowalski (antoni.kowalski@dandrite.au.dk)

Review Timeline:

Submission Date:	14th Mar 22
Editorial Decision:	8th Apr 22
Revision Received:	12th Jul 22
Editorial Decision:	15th Aug 22
Revision Received:	19th Sep 23
Accepted:	11th Oct 23

Editor: William Teale

Transaction Report:

(Note: With the exception of the correction of typographical or spelling errors that could be a source of ambiguity, letters and reports are not edited. Depending on transfer agreements, referee reports obtained elsewhere may or may not be included in this compilation. Referee reports are anonymous unless the Referee chooses to sign their reports.)

1st Editorial Decision 8th Apr 2022

Dear Poul,

Thank you again for the submission of your manuscript entitled "Monomeric α-Synuclein activates the Plasma Membrane Calcium Pump". We have now received the reports from the referees, which I copy below.

As you can see from their comments, your manuscript received a broad measure of support. However, all three referees raise their own questions and requests for clarification (some of which may require additional experimentation).

Based on the overall interest expressed in the reports, I would like to invite you to address the comments of all referees in a revised version of the manuscript. I should add that it is The EMBO Journal policy to allow only a single major round of revision and that it is therefore important to resolve the main concerns at this stage. I believe the concerns of the referees are reasonable and addressable, but please contact me if you have any questions, need further input on the referee comments or if you anticipate any problems in addressing any of their points. Please, follow the instructions below when preparing your manuscript for resubmission.

I would also like to point out that as a matter of policy, competing manuscripts published during this period will not be taken into consideration in our assessment of the novelty presented by your study ("scooping" protection). We have extended this 'scooping protection policy' beyond the usual 3 month revision timeline to cover the period required for a full revision to address the essential experimental issues. Please contact me if you see a paper with related content published elsewhere to discuss the appropriate course of action.

When preparing your letter of response to the referees' comments, please bear in mind that this will form part of the Review Process File, and will therefore be available online to the community. For more details on our Transparent Editorial Process, please visit our website: https://www.embopress.org/page/journal/14602075/authorguide#transparentprocess

Again, please contact me at any time during revision if you need any help or have further questions.

Thank you very much again for the opportunity to consider your work for publication. I look forward to your revision.

Best regards,

William

William Teale, Ph.D.
Editor
The EMBO Journal

When submitting your revised manuscript, please carefully review the instructions below and include the following items:

1) a .docx formatted version of the manuscript text (including legends for main figures, EV figures and tables). Please make sure that the changes are highlighted to be clearly visible.

2) individual production quality figure files as .eps, .tif, .jpg (one file per figure).

3) a .docx formatted letter INCLUDING the reviewers' reports and your detailed point-by-point response to their comments. As part of the EMBO Press transparent editorial process, the point-by-point response is part of the Review Process File (RPF), which will be published alongside your paper.

4) a complete author checklist, which you can download from our author guidelines (https://wol-prod-cdn.literatumonline.com/pb-assets/embo-site/Author Checklist%20-%20EMBO%20J-1561436015657.xlsx). Please insert information in the checklist that is also reflected in the manuscript. The completed author checklist will also be part of the RPF.

5) Please note that all corresponding authors are required to supply an ORCID ID for their name upon submission of a revised manuscript.

6) We require a 'Data Availability' section after the Materials and Methods. Before submitting your revision, primary datasets produced in this study need to be deposited in an appropriate public database, and the accession numbers and database listed under 'Data Availability'. Please remember to provide a reviewer password if the datasets are not yet public (see https://www.embopress.org/page/journal/14602075/authorguide#datadeposition). If no data deposition in external databases is needed for this paper, please then state in this section: This study includes no data deposited in external repositories. Note that

the Data Availability Section is restricted to new primary data that are part of this study.

Note - All links should resolve to a page where the data can be accessed.

7) When assembling figures, please refer to our figure preparation guideline in order to ensure proper formatting and readability in print as well as on screen:
http://bit.ly/EMBOPressFigurePreparationGuideline

Please remember: Digital image enhancement is acceptable practice, as long as it accurately represents the original data and conforms to community standards. If a figure has been subjected to significant electronic manipulation, this must be noted in the figure legend or in the 'Materials and Methods' section. The editors reserve the right to request original versions of figures and the original images that were used to assemble the figure.

8) For data quantification: please specify the name of the statistical test used to generate error bars and P values, the number (n) of independent experiments (specify technical or biological replicates) underlying each data point and the test used to calculate p-values in each figure legend. The figure legends should contain a basic description of n, P and the test applied. Graphs must include a description of the bars and the error bars (s.d., s.e.m.).

9) We would also encourage you to include the source data for figure panels that show essential data. Numerical data can be provided as individual .xls or .csv files (including a tab describing the data). For 'blots' or microscopy, uncropped images should be submitted (using a zip archive or a single pdf per main figure if multiple images need to be supplied for one panel). Additional information on source data and instruction on how to label the files are available at .

10) We replaced Supplementary Information with Expanded View (EV) Figures and Tables that are collapsible/expandable online (see examples in https://www.embopress.org/doi/10.15252/embj.201695874). A maximum of 5 EV Figures can be typeset. EV Figures should be cited as 'Figure EV1, Figure EV2" etc. in the text and their respective legends should be included in the main text after the legends of regular figures.

- For the figures that you do NOT wish to display as Expanded View figures, they should be bundled together with their legends in a single PDF file called *Appendix*, which should start with a short Table of Content. Appendix figures should be referred to in the main text as: "Appendix Figure S1, Appendix Figure S2" etc. See detailed instructions regarding expanded view here: .

- Additional Tables/Datasets should be labelled and referred to as Table EV1, Dataset EV1, etc. Legends have to be provided in a separate tab in case of .xls files. Alternatively, the legend can be supplied as a separate text file (README) and zipped together with the Table/Dataset file.

11) Our journal encourages inclusion of *data citations in the reference list* to directly cite datasets that were re-used and obtained from public databases. Data citations in the article text are distinct from normal bibliographical citations and should directly link to the database records from which the data can be accessed. In the main text, data citations are formatted as follows: "Data ref: Smith et al, 2001" or "Data ref: NCBI Sequence Read Archive PRJNA342805, 2017". In the Reference list, data citations must be labeled with "[DATASET]". A data reference must provide the database name, accession number/identifiers and a resolvable link to the landing page from which the data can be accessed at the end of the reference. Further instructions are available at .

Please make sure you upload a letter of response to the referees' comments together with the revised manuscript.

Please also check that the title and abstract of the manuscript are brief, yet explicit, even to non-specialists.

When assembling figures, please refer to our figure preparation guideline in order to ensure proper formatting and readability in print as well as on screen:
https://bit.ly/EMBOPressFigurePreparationGuideline
See also guidelines for figure legends: https://www.embopress.org/page/journal/14602075/authorguide#figureformat

IMPORTANT: When you send the revision we will require
- a point-by-point response to the referees' comments, with a detailed description of the changes made (as a word file).
- a word file of the manuscript text.
- individual production quality figure files (one file per figure)
- a complete author checklist, which you can download from our author guidelines (https://www.embopress.org/page/journal/14602075/authorguide).
- Expanded View files (replacing Supplementary Information)
Please see out instructions to authors
https://www.embopress.org/page/journal/14602075/authorguide#expandedview

Please remember: Digital image enhancement is acceptable practice, as long as it accurately represents the original data and conforms to community standards. If a figure has been subjected to significant electronic manipulation, this must be noted in the figure legend or in the 'Materials and Methods' section. The editors reserve the right to request original versions of figures and the original images that were used to assemble the figure.

Further information is available in our Guide For Authors: https://www.embopress.org/page/journal/14602075/authorguide

We realize that it is difficult to revise to a specific deadline. In the interest of protecting the conceptual advance provided by the work, we recommend a revision within 3 months (7th Jul 2022). Please discuss the revision progress ahead of this time with the editor if you require more time to complete the revisions. Use the link below to submit your revision:

https://emboj.msubmit.net/cgi-bin/main.plex

Referee #1:

EMBOJ-2022-111122
"Monomeric α-Synuclein activates the Plasma Membrane Calcium Pump"
Kowalski et al.

The authors report pull-down experiments that point to plasma membrane Ca2+-ATPase
(PMCA) as a potential interaction partner on monomeric alpha-synuclein (aSN). From proximity ligation assays we find that aSN and PMCA colocalize at neuronal synapses, and that calcium expulsion is activated by aSN and PMCA. From PMCA activity studies the authors show that soluble, monomeric aSN activates PMCA at par with CaM, yet independent of the autoinhibitory domain of PMCA, but highly dependent on acidic phospholipids and membrane-anchoring of aSN. On PMCA, the key site is mapped to the acidic lipid-binding site, located within a disordered PMCA-specific loop region connecting the cytosolic A domain and transmembrane segment 3. These studies point towards a novel physiological role of monomeric aSN as a stimulator of calcium clearance in neurons through activation of PMCA. Thus, aSN complements CaM in local compartments such as the presynapse. Overall, this is a comprehensive well done study and highlights a new role for monomeric aSN.

Comments

In Figure 1, can the authors pull down aSN with PMCA antibodies?

In the assays reported in Figure 2 can the authors examine the tetrameric form of alpha-syn described by the Selkoe group (PMID: 21841800 and related papers. It is clear that monomeric alpha-syn activates PMCA, while oligomeric does not. It would be of interest to determine the effect of tetrameric forms of alpha-syn and mutants 3K-aSN on the PMCA.

In Figure 4, It would be of interest to determine the effect of tetrameric forms of alpha-syn and mutants 3K-aSN on the PMCA.

It would be important to examine the effects of beta-synuclein and gamma-synuclein in comparison to monomeric alpha-syn activation of PMCA

Referee #2:

These are excellent studies reporting on a highly novel mechanism for activation of PMCA with broad relevance for understanding Ca2+ signaling and with immediate implication for understanding several forms of neurodegeneration. The authors report on activation of PMCA by Alpha-synuclein (aSN) and provide detailed mechanistical examination of the activation. The findings provide ample support for the conclusion of "a novel physiological role of monomeric aSN as a stimulator of calcium clearance in neurons through activation of PMCA".

I have fairly minor comments to strengthen a bit the relevance of the findings for non-neuronal cells and for the Ca2+ signaling community.

1. The cellular role of aSN is not sufficiently demonstrated. Since aSN activates both PMCA and SERCA their contribution to Ca2+ clearance should be evaluated. Further, the Ca2+ increase in the aSN expressing cells seems ablated compares to all others. The use of transfected aSN in generic cell, such as HeLa or HEK cells, and stimulation of Gq coupled receptor in the

presence of external Ca2+ and in the presence of absence of treatment with thapsigargin can address the relative importance of pumps to a receptor stimulated Ca2+ signal. Cells treated with thapsigargin in the presence of external Ca2+ and removal of external Ca2+ by perfusion with Ca2+-free solution containing EGTA can give a good and clear effect of aSN on PMCA. Finally, measurement of ER Ca2+ in such transfected cells can determine whether stimulation of SERCA by aSN contributes to the physiological Ca2+ signal.

2. Fig1: Since aSN activates both PMCA and SERCA, does aSN redistribute changes in response to increased cytoplasmic Ca2+ or cell stimulation.

3. The authors conclude that the plant PMCA is not stimulated by aSN, but close inspection suggests that the plant plant PMCA is stimulated by aSN when it is activated by CaM. This should be more carefully in the text.

Referee #3:

This review focuses on Fig. 1 which is the premise of the paper. Hopefully the other reviewers can give feedback on the other experiments.

- Fig. 1A: Please explain clearly how the co-ip was done, starting from the beginning. Cells, constructs, controls, inputs, pointing to what is what in the gel, etc. The writing is unclear, it seems there are two different kinds of IPs here? Also explain where exactly the bands are, and if they match with expected MWs. If not why? For example, in Fig. 1A blot on right, the PMCA bands seems to be squished (also, where are there 2 bands? Is this expected for PMCA?). Please show the entire blots in supp figs, so readers can make a learned judgement.

- Fig. 1B: Again, the PLA experiments are not described clearly. What was done, starting from the beginning? What is the rationale for using the a-syn -/-? Standard PLA controls (lacking one probe or the other, to rule out non-specific binding) are missing.

- Fig. 1C: No error bars. The main data are the mCherry only and mCherry + a-syn (is this human a-syn?). This needs to be shown separately. It is not clear if the Calcium uptake was similar in these two cases, because if not, that will surely affect the rate of K+ induced Ca++ release. What is the vanadate doing? Why was it used? There is some explanation but its hard to understand the rationale. Is the "KCl" arrow really pointing to when the KCl was added in these experiments, or where the peak of fluorescence is? (these should be two different things).

- Fig. 1D: Supposed to be quantification of Fura-2, but first, it is not mentioned in the main text, and it is unclear what it really being measured. Y-axis says cytosolic Ca level - when? at the peak? after complete release? At the end of experiment? None of these important things are mentioned. There are at least two things that should be quantified here - 1) extent of uptake (how well the Ca++ goes inside the neurons), and 2) the decay kinetics of release (what the rate of Ca++ release is).

Thoughts about statements made in the introduction:

- "Alpha-synuclein (aSN, 140 residues, and 14.5 kDa molecular weight) is an intrinsically disordered protein..." this statement is often thrown around but applies only to the C-term, and perhaps N-term in the absence of membranes, but maybe not as an overall descriptor of the protein.

- "However, the normal function of monomeric aSN remains an open question." Opinion is divided whether normally, a-syn is definitely monomeric in neurons, and certainly there is no data that can definitely say one way or another. So, should the question be framed like this?

- "Even at this level, the protein remains structurally disordered and monomeric in cells (Fauvet et al, 2012; Theillet et al., 2016)". This statement sides with papers that favor the monomer-centric view, but ignores many others that suggest that native a-synuclein is not monomeric.

- "The ability of aSN to interact with membranes is important for functions such as clustering of synaptic vesicles (Jo et al, 2000; Lautenschlager et al, 2018), regulation of the presynapse size (Vargas et al, 2017), and neurotransmitter release by promoting the formation of SNARE complexes (Burre et al, 2010)." Many of these "functions" of a-synuclein are essentially extrapolations from studies where all synucleins have been deleted. This should be written in this manner (what the experimenters actually did, not what they say it is), so readers can make up their own mind.

- "The C-terminal region, comprising residues 96-140 remains disordered regardless of interactions...". How do the authors know this? Has it been shown that interactors do not change disorder in this region after they are bound? If not, then this is a speculation and should not be presented as a fact (similar to previous point). Or could be presented as a speculation, but that is not how it sounds right now.

- "PMCA is a single polypeptide transmembrane protein" - Single pass?

EMBO Journal
William Teale, editor

Dear William Teale
We are pleased to submit our revised manuscript "*Monomeric α-Synuclein activates the Plasma Membrane Calcium Pump*" by Kowalski *et al*.
We are grateful for the constructive reviews and provide new data and revised text and figures. We hope the reviewers and you will agree that we have addressed the points satisfactorily and in a relevant manner. In short, we have provided new data on the direct interactions and modified the text when ambiguous. Generally, we have also revised the text for improved readability. We will upload a clean version and also include a mark-up version (which will be very busy) for reference.
On behalf of all authors

Poul Nissen
Aarhus, July 12th, 2022

Point-by-point response to reviewers
Reviewer comments
Our responses including *citations from the revised manuscript in italic*

Referee #1:

EMBOJ-2022-111122
"Monomeric α-Synuclein activates the Plasma Membrane Calcium Pump"
Kowalski et al.

The authors report pull-down experiments that point to plasma membrane Ca2+-ATPase (PMCA) as a potential interaction partner on monomeric alpha-synuclein (aSN). From proximity ligation assays we find that aSN and PMCA colocalize at neuronal synapses, and that calcium expulsion is activated by aSN and PMCA. From PMCA activity studies the authors show that soluble, monomeric aSN activates PMCA at par with CaM, yet independent of the autoinhibitory domain of PMCA, but highly dependent on acidic phospholipids and membrane-anchoring of aSN. On PMCA, the

key site is mapped to the acidic lipid-binding site, located within a disordered PMCA-specific loop region connecting the cytosolic A domain and transmembrane segment 3. These studies point towards a novel physiological role of monomeric aSN as a stimulator of calcium clearance in neurons through activation of PMCA. Thus, aSN complements CaM in local compartments such as the presynapse. Overall, this is a comprehensive well done study and highlights a new role for monomeric aSN.

Comments

In Figure 1, can the authors pull down aSN with PMCA antibodies?

Thank you for many valuable comments. To address this suggestion, we performed the co-immunoprecipitation assay. We decided to address this point both *in vitro* by immobilized, purified PMCA and purified aSN, and by coIP studies in cells. Using PMCA immobilized to CaM Sepharose resin, we found specific binding of aSN (see Fig. 1B). These are simple and quite strict experiments that reverse the order of the earlier and current coIP results with aSN antibodies. Furthermore, they are exempt of many indirect effects that may influence coIP studies in both positive and negative directions.

For coIP studies, we used red blood cell lysates, where both PMCA and aSN are abundant. However, the experiments did not bring conclusive results. This can be due to many reasons such as incompatible epitopes, conformational changes, and loss of functional integrity of immunoprecipitated PMCA, and we decided not to go further down that road, also knowing that the *in vitro* studies using CaM-sepharose immobilized PMCA were clear and conclusive. We fully agree that our findings merit further studies of the interactions *in vivo*, but we think for this report on the initial findings, our conclusions are firmly established with the purified *in vitro* interaction study.

In the assays reported in Figure 2 can the authors examine the tetrameric form of alpha-syn described by the Selkoe group (PMID: 21841800 and related papers. It is clear that monomeric alpha-syn activates PMCA, while oligomeric does not. It would be of interest to determine the effect of tetrameric forms of alpha-syn and mutants 3K-aSN on the PMCA.

In Figure 4, It would be of interest to determine the effect of tetrameric forms of alpha-syn and mutants 3K-aSN on the PMCA.

We thank for the suggestion; however, to our knowledge the tetrameric form of alpha-synuclein cannot be purified in a form available for biochemical assay, and the tetramers studied to date have been stabilized by cross-linking.

In our study we show that the lipid-binding capacity and availability of the N-terminal region of aSN is crucial for the activation of PMCA. We expect that any form where these features are limited or restrained, including tetramers, would show weakened effect on PMCA activity. We argue that the results showing the effect of oligomerization, A30P mutation or truncated variants of aSN are sufficient for the key conclusions and discussions of this report.

It would be important to examine the effects of beta-synuclein and gamma-synuclein in comparison to monomeric alpha-syn activation of PMCA

This is a good point, and we have included data and a figure showing the effect of beta- and gamma- synuclein on the activity of PMCA1d. Both isoforms activated the PMCA, although lower than alpha-synuclein. The results are consistent with our finding that the N-terminal region of aSN is of relevance for the PMCA stimulation as the N-terminus is highly conserved among the aSN isoforms. The results are presented on the Figure EV1.

Referee #2:

These are excellent studies reporting on a highly novel mechanism for activation of PMCA with broad relevance for understanding Ca2+ signaling and with immediate implication for understanding several forms of neurodegeneration. The authors report on activation of PMCA by Alpha-synuclein (aSN) and provide detailed mechanistical examination of the activation. The findings provide ample support for the conclusion of "a novel physiological role of monomeric aSN as a stimulator of calcium clearance in neurons through activation of PMCA".

I have fairly minor comments to strengthen a bit the relevance of the findings for non-

neuronal cells and for the Ca2+ signaling community.

1. The cellular role of aSN is not sufficiently demonstrated. Since aSN activates both PMCA and SERCA their contribution to Ca2+ clearance should be evaluated. Further, the Ca2+ increase in the aSN expressing cells seems ablated compares to all others. The use of transfected aSN in generic cell, such as HeLa or HEK cells, and stimulation of Gq coupled receptor in the presence of external Ca2+ and in the presence of absence of treatment with thapsigargin can address the relative importance of pumps to a receptor stimulated Ca2+ signal. Cells treated with thapsigargin in the presence of external Ca2+ and removal of external Ca2+ by perfusion with Ca2+-free solution containing EGTA can give a good and clear effect of aSN on PMCA. Finally, measurement of ER Ca2+ in such transfected cells can determine whether stimulation of SERCA by aSN contributes to the physiological Ca2+ signal.

This is indeed good suggestions for a more in-depth studies of the mechanisms, however we have focused on the biochemical/biophysical findings of an interaction, and so far qualified the observations in cells. aSN is predominantly found in neuronal terminals, explaining our choice to conduct the calcium measurements in primary neuronal cultures with or without transient expression of aSN. Since the expression of aSN is transient, it is produced over a few days, and we have no indications of oligomerization, or aggregation of aSN. SERCA is primarily stimulated by oligomeric, or aggregated aSN (Betzer et al., 2018, https://doi.org/10.15252/embr.201744617), and based also on the findings of the present report we think the effect of monomeric aSN on SERCA is minimal, also taking into account that the SERCA inhibitor, thapsigargin was present during our calcium recordings. Calcium measurements directly in the ER are not easily conducted since the calcium concentration in ER is in mM and the cytosol nM. We certainly agree that such studies are interesting and should be pursued, but it will not change the key conclusions and discussions presented in the current manuscript, and therefore we would argue that such experiments will be in the framework of new, downstream projects.

2. Fig1: Since aSN activates both PMCA and SERCA, does aSN redistribute changes in response to increased cytoplasmic Ca2+ or cell stimulation.

This is an interesting point, however in this study we only focus on activation of PMCA, which we believe is the important biological role of monomeric aSN, based on the biochemical observations. As shown in a previous study (Betzer et al., 2018 https://doi.org/10.15252/embr.201744617), SERCA is primarily stimulated by aggregated forms of aSN, and thus associated with a pathological state of aSN function. We consider the comment very valuable and worth taking into account when designing further studies on the effect of aSN on cellular calcium homeostasis.

3. The authors conclude that the plant PMCA is not stimulated by aSN, but close inspection suggests that the plant PMCA is stimulated by aSN when it is activated by CaM. This should be more carefully in the text.

Thank you for the remark. We agree, but also note that the effect is weak, and difficult to draw strong conclusions from. We made a correction to make the text correspond better with the data. Now it says:

In the presence of BE, ACA8 is not activated by aSN, but rather inhibited, and the effect is reversed by CaM. This may suggest an interplay between aSN, CaM and acidic lipids, that results in a different stimulation pattern, albeit weak, for the plant pump ***(Figure 5B)****.*

Referee #3

- Fig. 1A: Please explain clearly how the co-ip was done, starting from the beginning. Cells, constructs, controls, inputs, pointing to what is what in the gel, etc. The writing is unclear, it seems there are two different kinds of IPs here? Also explain where exactly the bands are, and if they match with expected MWs. If not why? For example, in Fig. 1A blot on right, the PMCA bands seems to be squished (also, where are there 2 bands? Is this expected for PMCA?). Please show the entire blots in supp figs, so readers can make a learned judgement.

Thanks for valuable comments on this part. We apologize for the unclear description. The text and figures have been modified for improved clarity and now states:

Co-immunoprecipitation experiments with brain homogenate from aSN-knockout mice supplemented with purified aSN monomer revealed aSN binding, and a similar experiment

*with aSN oligomer showed the same result indicating no discrimination on aSN conformation (**Figure 1A, left**). Co-immunoprecipitation and western blotting of endogenous aSN and PMCA from detergent extracts of total brain homogenates from wild-type C57BL/6 mice show that endogenous PMCA interacts with endogenous aSN (**Figure 1A, right**). PMCA appears with multiple bands, or a smear on western blots from the two co-IPs, likely due the varying amounts of post translational modifications of PMCA (Tolosa de Talamoni et al, 1993). Moreover, a pull-down assay performed with purified proteins showed aSN binding to PMCA immobilized on Calmodulin sepharose (**Figure 1B**).*

Full blots are now included in the Appendix – Figure S6. Two bands are expected as demonstrated, and probably represent different degrees of maturation in the biosynthetic pathway like glycosylation (figure posted here https://www.researchgate.net/publication/14932947_Immunocytochemical_Localization_of_the_Plasma_Membrane_Calcium_Pump_Calbindin-D28k_and_Parvalbumin_in_Purkinje_Cells_of_Avian_and_Mammalian_Cerebellum)

- Fig. 1B: Again, the PLA experiments are not described clearly. What was done, starting from the beginning? What is the rationale for using the a-syn -/-? Standard PLA controls (lacking one probe or the other, to rule out non-specific binding) are missing.

Thanks for pointing this out. The text has been modified for improved clarity and now states:

The interaction between aSN and PMCA was investigated further in primary hippocampal neurons derived from newborn C57BL/6 mice and with neurons from aSN-KO mice as negative controls. Primary hippocampal neurons were fixed and analyzed after 14 days in culture by immunofluorescence labeling of synapses by synaptophysin and an aSN/PMCA proximity ligation assay (PLA). The PLA is used for in situ detection of protein interactions and is based on two primary antibodies from different species, here ASY-1, a polyclonal aSN antibody generated in rabbit, and 5F10, a monoclonal pan PMCA antibody generated in mouse. The secondary antibodies were labeled with oligonucleotides that enzymatically can be ligated when in close proximity and then amplified, hence generating concatemeric sequences. These sequences can bind fluorescently labelled oligonucleotides yielding a red fluorescent signal when aSN and PMCA are located within approximately 40nm. PMCA and aSN were found to be in this proximity of each other and located to

*the synapses **(Figure 1C)**.*

- and in methods section:

Proximity ligation assay (PLA) enables in situ detection of protein-protein interaction using single-stranded oligonucleotides conjugated to specie specific antibodies. If the oligonucleotides are in close proximity (within 40 nm), they can be ligated to form circular DNA, which can be amplified by multiplying the binding site for fluorescently labelled complementary oligonucleotides and thus multiplying the fluorescent readout.

Furthermore, the traditional negative PLA control lacking ligase has been added. Note, that the figure has now the number 1C.

Fig. 1C:

- No error bars.

In the revised manuscript the figure has the number 1D. The figure is shown as representative curves of a single neuron from each condition. The curves from individual cells varied due to transient expression of aSN leading to varying amount of the protein at the time of analysis, and we believe a representative curve is the simplest and better presentation of the data. All data are integrated in Fig.1E, which we now present as a scatter-dot plot. We have now more clearly stated in the figure legend: ***D*** *– Representative curves of single neurons expressing....*

- The main data are the mCherry only and mCherry + a-syn (is this human a-syn?).

Yes

This needs to be shown separately. It is not clear if the Calcium uptake was similar in these two cases, because if not, that will surely affect the rate of K+ induced Ca++ release.

We thank for the constructive feedback. We agree that the presentation of the Ca2+ intake was not optimal in figure 1D. Therefore, we have made the lines thinner for better visualization of each condition and moved the mCherry/aSN on top of the other curves. However, we do believe the PMCA inhibition by vanadate are important data to show together with the mCherry only and mCherry + aSN, as these data highlight the PMCA-contribution in the aSN induced Ca2+ efflux-increase.

The mCherry/aSyn neuron does indeed not reach the same peak FURA2 levels as the other conditions. We believe this is due to the increased activity of PMCA already compensating for the high influx of Ca2+. This is supported by the addition of vanadate, which reverse the effect aSN expression in the neurons.

What is the vanadate doing? Why was it used? There is some explanation but its hard to understand the rationale.

Vanadate is a well-established inhibitor of P-type ATPases and used in previous studies to study the effect of PMCA. We used vanadate to support that the observed effect of aSN-expression was due to PMCA and not adverse effects of aSN expression. We agree that this was not communicated clearly in the manuscript, and the text has now been modified to say:

Vanadate is a well-known phosphatase inhibitor and a potent inhibitor of P-type ATPases (Bond & Hudgins, 1980; Cantley et al, 1978; Dupont & Bennett, 1982) and was used as a PMCA inhibitor in this experiment, since SERCA was inhibited by thapsigargin from the start. The vanadate decreased the calcium expulsion of aSN mCherry neurons to the same level as mCherry-only neurons treated with vanadate. This supports that increased efflux induced by aSN is driven by PMCA.

Is the "KCl" arrow really pointing to when the KCl was added in these experiments, or where the peak of fluorescence is? (these should be two different things).

We thank for the observation. The KCl arrow was indeed placed incorrectly at the peak of fluorescence. It has now been moved a bit to the left to indicate the correct timepoint for addition of KCl.

Fig. 1D: Supposed to be quantification of Fura-2, but first, it is not mentioned in the main text, and it is unclear what it really being measured. Y-axis says cytosolic Ca level - when? at the peak? after complete release? At the end of experiment? None of these important things are mentioned.

In the revised manuscript the figure has the number 1E. In the figure legend we state:
E – Cytosolic Ca2+ level after the KCl-induced influx, quantified as the area under the curve (AUC±SEM). The response upon K+ induced depolarization was quantified as the Area Under Curve (AUC) from each measured neuron in the 2-12 min. interval.

There are at least two things that should be quantified here - 1) extent of uptake (how well the Ca++ goes inside the neurons), and 2) the decay kinetics of release (what the rate of Ca++ release is). This is likely difficult because the uptake is countered by the extrusion through the pumps (PMCA but also SERCA).

These are very relevant comments and inputs. As explained, the aSN activated PMCA pool is most likely active already during the KCl evoked influx of Ca2+, which results in a lower maximum intake. We have thapsigargin present in excess amounts, so it should only be PMCA effect we see. This has been made clearer now in the text: *SERCA was inhibited by thapsigargin (4µM) before recording*

The calcium extrusion experiments are of qualitative nature, but with significant conclusion as shown in Fig. 1E. Establishing a quantitative model for PMCA activation and calcium fluxes in cells would be a different and far more elaborate study - we prefer in this report to focus the quantitative assessment of the interaction on the biochemical/biophysical studies, in particular the read-out of ATPase activity, which is tightly coupled to PMCA transport function.

Thoughts about statements made in the introduction:

- "Alpha-synuclein (aSN, 140 residues, and 14.5 kDa molecular weight) is an intrinsically disordered protein..." this statement is often thrown around but applies

only to the C-term, and perhaps N-term in the absence of membranes, but maybe not as an overall descriptor of the protein.

Thanks for the comments regarding the introduction. We have simplified the opening sentence by removing the phrase “intrinsically disordered”. Description of the structural features of aSN are better explained further down in the introduction.

- "However, the normal function of monomeric aSN remains an open question." Opinion is divided whether normally, a-syn is definitely monomeric in neurons, and certainly there is no data that can definitely say one way or another. So, should the question be framed like this?

We changed “monomeric” to “native” and added a short note regarding current hypotheses on normal functions.

- "Even at this level, the protein remains structurally disordered and monomeric in cells (Fauvet et al, 2012; Theillet et al., 2016)". This statement sides with papers that favor the monomer-centric view, but ignores many others that suggest that native a-synuclein is not monomeric.

We referred to reports suggesting the existence of native tetramer. The relevant paragraph now states:

Importantly, aSN is highly abundant in the presynaptic region of neurons, where concentrations reach 5-50 microM (Bodner et al, 2009; Perni et al, 2017; Theillet et al, 2016). It is likely involved in homeostasis of synaptic vesicle release (Sulzer & Edwards, 2019) although mechanisms remain unclear, but a chaperone function of SNARE proteins has been proposed (Burre et al, 2010). A tetrameric form was isolated from cells after in vivo cross-linking (Dettmer et al, 2013; Dettmer et al, 2015a); however, the intact aSN appears structurally disordered and monomeric in cells (Fauvet et al, 2012; Theillet et al., 2016).

- "The ability of aSN to interact with membranes is important for functions such as clustering of synaptic vesicles (Jo et al, 2000; Lautenschlager et al, 2018), regulation of the presynapse size (Vargas et al, 2017), and neurotransmitter release by promoting the formation of SNARE complexes (Burre et al, 2010)." Many of these "functions" of a-

synuclein are essentially extrapolations from studies where all synucleins have been deleted. This should be written in this manner (what the experimenters actually did, not what they say it is), so readers can make up their own mind.

We agree that the statement in the previous version was too short. We changed the text to indicate that these are proposed, not well-established functions:

The membrane interaction of aSN was suggested to play a role in clustering of synaptic vesicles (Jo et al, 2000; Lautenschlager et al, 2018), regulation of the presynapse size (Vargas et al, 2017), and neurotransmitter release (Burre et al., 2010).

- "The C-terminal region, comprising residues 96-140 remains disordered regardless of interactions...". How do the authors know this? Has it been shown that interactors do not change disorder in this region after they are bound? If not, then this is a speculation and should not be presented as a fact (similar to previous point). Or could be presented as a speculation, but that is not how it sounds right now.

We have changed the sentence to:

The C-terminal region, comprising residues 96-140, appears as constitutively disordered, has a strong negative charge, and binds calcium ions with low affinity (Eliezer et al, 2001; Lautenschlager et al., 2018; Nielsen et al, 2001).

- "PMCA is a single polypeptide transmembrane protein" - Single pass?

PMCA is a polytopic transmembrane protein, with 10 transmembrane alpha-helices. Addressing this feature in this sentence has minor relevance for the introduction, thus we decided to shorten the sentence to: *PMCA is a transmembrane protein of 130-140 kDa….* Readers can also get the information about the overall PMCA structure from the schematic figures in the results section.

1st Revision - Editorial Decision 15th Aug 2022

Dear Poul,

Thank you for submitting your revised manuscript to The EMBO Journal. As William is currently away on holidays, I have stepped in as 2nd editor on the manuscript to help move things along.

Your study has now been seen by the original three referees and their comments are provided below.

As you will see, referee 2 maintains a strong opinion that your study would benefit from a few additional experiments. In order to make as fair a decision as possible on whether to ask you to perform them, we also consulted referee 1, specifically on this point. Referee 1 was of the opinion that the experiments would be achievable and encourages their inclusion.

As adding this extra breadth would significantly increase the impact of your study and can be done in a realistic time-frame, I would also like these experiments (addressing the relative influence of aSN on PMCA and SERCA in HEK or Hela cells) to be included in the manuscript.

Referee #3 also raises a very relevant concern regarding Figure 1D. Do you have data to further address this point?

I would like to ask you to start thinking about the needed revisions for consideration here. William is back next week and please touch base with him then about the needed revisions.

With best wishes

Karin

Karin Dumstrei, PhD
Senior Editor
The EMBO Journal

Use the link below to submit your revision:

https://emboj.msubmit.net/cgi-bin/main.plex

Referee #1:

The authors have addressed my concerns or with reasonable rebuttals.

Referee #2:

The authors failed to address any of my concerns by stating we decided to focus on neuronal cells and all your suggestions are not relevant for us. I completely disagree. EMBO manuscripts are intended for a diverse audience and extending the studies to be relevant to the enormous Ca2+ signaling community interested in cells other than neuronal cells is a must. Moreover, the experiments are suggested are needed to clarify important point related to results in the manuscript. Claiming that measurement of ER Ca2+ is complicated is strange-to say the least- with many thousands of such studies reported in the literature! Therefore, unfortunately, I cannot support publication of this important manuscript in its current form.

Referee #3:

Unfortunately, the data in this paper do not support the authors' conclusions.

The key here is Fig. 1D (revision), where the effect of a-syn on Ca++ uptake is measured by Fura 2. The authors' hypothesis is that a-syn binds to PMCA and FACILITATES calcium intake. The Fura 2 data clearly show that in the presence of a-syn, there is LESS increase in Fura 2 fluorescence upon neuronal activation, which means that there is LESS Ca++ uptake, which is the opposite of what the authors are saying - "Neurons expressing aSN expelled calcium to a markedly higher degree than the mCherry expressing neurons (Figure 1D-E)." ("expelled" is probably wrong usage here, implying expelling from cells - the increase of Ca++ in intracellular - probably the authors mean expelled from organelles, but this is unclear).

Among all the noise in figure 1D (which I asked the authors to eliminate but they didn't), there are only two lines to consider

carefully - red (mCherry only) and blue (mCherry + aSN). Clearly, the peak of the blue curve (indicating how much Ca++ intake occurred in the presence of a-syn) is lower than the peak of the red curve (no a-syn, since these are a-syn -/- neurons). This means that in the presence of a-synuclein, LESS Ca++ entered the neurons when they were stimulated. The obfuscating statistics shown in Figure 1E are not helpful.

In the rebuttal, the authors say that this is the reason for the lower a-syn peak: "We believe this is due to the increased activity of PMCA already compensating for the high influx of Ca2+", which does not make any sense to this reviewer. All experiments were done in a-syn -/- neurons, and without neuronal activity, there should not be much Ca++ intake.

The simplest explanation of the data is that in the presence of a-syn, there is less activity-induced uptake of Ca++, which is the opposite of what is written in the paper.

Comments from the referees

comments from editor

Our responses

Editorial comments from Sep. 20th, 2023 (William Teale):

- We noticed that contributing author Alicia Espiña Bou has not been entered in our online system.
Could you please add an account for this author to ensure that we have the complete list?

Done

- Please provide the Point-by-point response to reviewers' comments

Presented here

- Please upload each of the main and EV Figures as an individual file, with sufficient resolution/quality for production.

Done

- On the abstract page of the manuscript, please include 4-5 general keyword terms to enhance searchability.

Done

- Rename the Conflict of Interest Section as the "DISCLOSURE AND COMPETING INTERESTS STATEMENT" according to the instructions on our website. Please add the following disclaimer: "Poul Nissen is a member of the Advisory Editorial Board of The EMBO Journal. This has no bearing on the editorial consideration of this article for publication."

Done

- As we are switching from a free-text author contribution statement towards a more formal statement based on Contributor Role Taxonomy (CRediT) terms, please specify each author's contribution(s) directly in the Author Information page of our submission system during upload of the final manuscript, and remove the current Author Contribution section in the text. See https://casrai.org/credit/ for more information.
Done

- Please double-check and make sure that the funding info in text and in submission system is congruent: missing in manuscript file: R155-2015-2666; Natur og Univers, Det Frie Forskningsråd (FNU, DFF). Missing in the submission system: R223-2015-4222; Novo Nordisk Foundation (#NNF18OC0033926 to B.B.K); The Berlin Mathematics Research Center MATH+ (EXC-2046/1, project ID: 390685689)

Done

- Please use 10 authors + et al. in the reference section

Done

- Please address: n=2 in Figure 5A

Done, we now write on page 11: "…in the same lipid-dependent manner as PMCA (**Figure 5A**). From the two experiments performed, the effect was only predominant in low (60 nM) free Ca2+ concentration, whereas at high 1.8 µM free Ca2+, SERCA was activated to a lower fold"

- Please adjust the in-text callouts for individual figures and figure panels: callouts for: Figure EV1 and Appendix figure panels are missing.
Done

- Please resize the Synopsis image to 550 pixels wide x 200-400 pixels high

Done

- Please provide suggestions for a short 'blurb' text prefacing and summing up the conceptual aspect of the study in two sentences (max. 250 characters), followed by 3-5 one-sentence 'bullet points' with brief factual statements of key results of the paper; they will form the basis of an editor-written 'Synopsis' accompanying the online version of the article.

Done, uploaded

- Table EV1 should be uploaded separately using the file type Expanded View. The legend should be also removed from the manuscript file and placed above the Table EV1

Done

- Please rename all "supplemental" figures and tables into "Appendix Figure S1/2/3.." or "Appendix Table S1/2/3...", both when referencing them in the text, and within the single Appendix PDF, in which they should all be combined. The Appendix should have page numbers, and start with a brief table of contents listing the included figures, legends and tables and their respective page numbers. Appendix Figure S2 panels should be A-D not I-IV.

Done

Editorial comments from Sep. 9th, 2022 (William Teale):

Firstly, I would like to thank you for the recent effort you have taken to clarify the current situation regarding manuscript EMBOJ-2022-111122 'Monomeric α-Synuclein activates the

Plasma Membrane Calcium Pump'. I have now had a chance to take a fresh look at the manuscript and have discussed it, along with your responses to the reviewers, again with the editorial team here at EMBO Journal. Your manuscript defines a role for monomeric α-Synuclein in stimulating PMCA to remove calcium ions from neurons. Furthermore, you show that this function is regulated by the anchoring of α-Synuclein by acidic membrane lipids. These are clearly conclusions of interest to many areas of research and I acknowledge that it would be of high interest to our readership.

thanks!

In his/her report, Reviewer 2 asked for new data to be included in the study which would place the regulation of PMCA by α-Synuclein in a more cellular context. It was suggested that you measure the relative effects of α-Synuclein on the ER calcium pump SERCA and the PM-localised PMCA. Unfortunately, non-scientific constraints mean that you are currently unable to perform the suggested experiment, or add any of the requested further context.

The study in primary neurons motivated the biochemical/biophysical study to show that aSN activates PMCA, similar to calmodulin, but at complementary lipid conditions. I think reviewer 2 takes it the other way round. Furthermore, we map the interactions sites on PMCA and aSN and find specificity to PMCA although with some effect also on SERCA. These are truly surprising, novel result on PMCA activation that obviously invite many new studies. Taking it further to cell biological studies and calcium dynamics across organelles is a certainly one good suggestion, but far from simple and easy for non-expert laboratories, and it represents a new, expanded study.

On that note, yes: all the young researchers involved were going into new positions or on maternity leave, so we also were not able to act fast and extensively on such a request to expand the study towards new experimental modalities, conclusions, and discussions.

In his/her report on the revised manuscript, Reviewer 2 maintains that, for the EMBO Journal readership fully to assess your results from their diverse perspectives, a broader context needs to be given. On this point, I agree. I therefore suggest I approach EMBO Reports, a venue much more suited to the type of concise and direct study you present, with a view to transferring your manuscript there. Our scoop protection policy will cover your work through this process. If you are able to arrange an extra small round of cell biological experiments to be done, I would be very happy to publish your manuscript in EMBO Journal.

We do not see the format of EMBO Reports fitting quite as well to the scale of our study. We therefore opted for added cell biological experiments, as described also in the current cover

letter and below. Unfortunately, the new experiments have taken us a long time to initiate and proceed.

We have applied the PMCA mediated Ca2+ expulsion experiment (SERCA blocked) in SH-SY5Y, PC12 and chromaffin cells with aSN expression. This is a good and sensitive assay that works well when preconditions are met. For SH-SY5Y, the trend was quite convincing for the few cells showing depolarization: aSN expression stimulates Ca2+ expulsion, although to a lower degree than primary hippocampal neurons, and the control experiments with vanadate blocking PMCA activity did not show significant alterations with the limited number of experiments accrued (many batches of the aSN inducible SH-SY5Y cells do not show a depolarization response to KCl , as also previously reported by Morton, Hammond et al., 1992, referenced in the revised version). For PC12, we saw no effect of aSN expression. Native aSN expression, poor PMCA expression or many other possible factors can be the reason; we did not pursue this further. For chromaffin cells, KCl depolarization did not stimulate Ca2+ influx.

Response from reviewers to the previously submitted version (Sep. 2022)

our responses

Referee #1:

The authors have addressed my concerns or with reasonable rebuttals.

We are grateful for this comment by reviewer 1

Referee #2:

The authors failed to address any of my concerns by stating we decided to focus on neuronal cells and all your suggestions are not relevant for us. I completely disagree.

We certainly do not find the comments irrelevant, but it was difficult for us to conduct these suggested experiments to expand the scope of the paper.

EMBO manuscripts are intended for a diverse audience and extending the studies to be relevant to the enormous Ca2+ signaling community interested in cells other than

neuronal cells is a must. Moreover, the experiments are suggested are needed to clarify important point related to results in the manuscript. Claiming that measurement of ER Ca2+ is complicated is strange-to say the least- with many thousands of such studies reported in the literature! Therefore, unfortunately, I cannot support publication of this important manuscript in its current form.

We were very pleased of the reviewer's first response to our manuscript that essentially approved our experimental findings, conclusion and discussion, and we interpreted the comments for further experiments and expanded scope as suggestions, not as critical points (quoting: "*I have fairly minor comments to strengthen a bit the relevance of the findings for non-neuronal cells and for the Ca2+ signaling community*").

We think the mechanism we present is most relevant in cells where aSN abundance is high and PMCA is critical for Ca2+ homeostasis, such as blood cells and neurons. We considered it unrealistic within the time range of a revision to set up an entirely new cell model with a GPCR coupled calcium signal and new imaging experiments, also given the practical restraints that the project grant is terminated, and the postdoc (and first-author) is leaving the laboratory.

We certainly agree that the Referee's suggestions to study aSN effects on calcium dynamics between ER and plasma membrane would be a great follow-up step to the Betzer et al., 2018 paper and the present study, but to make any firm conclusions on a new cell model and from experiments addressing those points will require very significant time and resources for us, also given the fact that for example HEK293 cells have a background aSN expression to account for.

We will argue that the key findings we report are original and well-established from the presented data, and that neurons (and blood cells) are the relevant cell models to address this functional link, and where the findings have the most immediate implications due to their strong dependence on PMCA activity for calcium homeostasis and large aSN expression (as discussed in the paper).

Referee #3:

Unfortunately, the data in this paper do not support the authors' conclusions.

Everything is consistent, see below

The key here is Fig. 1D (revision), where the effect of a-syn on Ca++ uptake is measured by Fura 2. The authors' hypothesis is that a-syn binds to PMCA and FACILITATES calcium intake.

The observation is that aSN activates PMCA and facilitates calcium extrusion, not intake.

The Fura 2 data clearly show that in the presence of a-syn, there is LESS increase in Fura 2 fluorescence upon neuronal activation, which means that there is LESS Ca++ uptake, which is the opposite of what the authors are saying - "Neurons expressing aSN expelled calcium to a markedly higher degree than the mCherry expressing neurons (Figure 1D-E)." ("expelled" is probably wrong usage here, implying expelling from cells - the increase of Ca++ in intracellular - probably the authors mean expelled from organelles, but this is unclear).

We report that plasma-membrane Ca2+-ATPase (PMCA - a calcium pump, not a channel), is stimulated by aSN to pump out ("expel") Ca2+ to the extracellular environment. This is evident everywhere in the title, abstract and text.

In our cell model, intracellular calcium levels revealed by FURA-2 fluorescence result from two oppositely oriented reactions: 1) calcium *influx* to the cell via voltage-gated channels responding to KCl induced depolarization, and 2) calcium *expulsion* by PMCA actively transporting Ca2+ from the cytosol to extracellular space (SERCA pumping Ca2+ to intracellular stores is blocked by thapsigargin). PMCA is a high-affinity pump and it contributes to calcium clearance down to a low nanomolar level. Calcium spikes will also be affected by its activity. Neurons express a lot of fast-reacting PMCA2 and PMCA3 isoforms. In the paper, we show that aSN stimulates PMCA's activity in neurons. Thus, the lower net influx of calcium in aSN-expressing cells is consistent, and we explain it as the effect of more rapid response due to activation of PMCA by aSN.

Among all the noise in figure 1D (which I asked the authors to eliminate but they didn't), there are only two lines to consider carefully - red (mCherry only) and blue (mCherry + aSN). Clearly, the peak of the blue curve (indicating how much Ca++ intake occurred in the presence of a-syn) is lower than the peak of the red curve (no a-syn, since these are a-syn -/- neurons). This means that in the presence of a-synuclein,

LESS Ca++ entered the neurons when they were stimulated. The obfuscating statistics shown in Figure 1E are not helpful.

We are puzzled about the Reviewer's take on figure 1D. In reply to the previous comments, we adjusted the figure to make it clearer, and all the representative curves are now easy to distinguish. The reason why we show the four representative curves (including also +/- vanadate) was explained earlier to show PMCA dependent and aSN stimulated Ca2+ extrusion. The bar graph in figure 1E is the scatter plot to show the single quantifications - it would be a mistake not to include this quantitative assessment of the pooled experiments. The scatter plots indicate some variability between measurements of individual cells and experiments, but the results are significant to show that aSN stimulates PMCA-dependent calcium extrusion.

PMCA counteracts the influx by an immediate response of activity to the increased free Ca2+ concentration in the cytosol. The blue line shows PMCA being more active with aSN than without (red line).

We will make a stronger point in the text of the fact that aSN activation is immediate and Ca2+ independent, in contrast to the Ca2+ dependent activation by calmodulin, and therefore cells/compartments with aSN activated PMCA respond instantly to calcium influx.

In the rebuttal, the authors say that this is the reason for the lower a-syn peak: "We believe this is due to the increased activity of PMCA already compensating for the high influx of Ca2+", which does not make any sense to this reviewer. All experiments were done in a-syn -/- neurons, and without neuronal activity, there should not be much Ca++ intake.

The simplest explanation of the data is that in the presence of a-syn, there is less activity-induced uptake of Ca++, which is the opposite of what is written in the paper.

Everything is consistent. The experiments were performed in living cells exhibiting neuronal activity. Cells were depolarized by KCl, which induces strong Ca2+ influx via voltage-gated Ca2+ channels. The experiments probe the dynamically changing levels of cytosolic free Ca2+, and the red curve is the control. The Ca2+ level observed when PMCA is inhibited by vanadate (black and yellow lines and bars) is therefore high, whereas cells with aSN-stimulated PMCA activity counteracting the calcium influx yield a lower Ca2+ level (blue line/bar).

2nd Revision - Editorial Decision 11th Oct 2023

Dear Poul,

Referee 2 has now replied with a note of qualified acquiescence; the report is attached below. I am therefore pleased to inform you that your manuscript has been accepted for publication in the EMBO Journal.

Congratulations to you and your co-authors. I am personally really glad this work will be published with us.

Referee #2:

I do not agree with the response of the authors or think that it is satisfactory- all the suggested simple experiments could have been done by collaboration within their institute and I know of several investigators that could have help. Nevertheless, I think the results are important and need to be published. Therefore, to prevent rejection, I will support publication.

Please note that it is EMBO Journal policy for the transcript of the editorial process (containing referee reports and your response letter) to be published as an online supplement to each paper. If you do NOT want this, you will need to inform the Editorial Office via email immediately. More information is available here:
https://www.embopress.org/page/journal/14602075/authorguide#transparentprocess

Your manuscript will be processed for publication in the journal by EMBO Press. Manuscripts in the PDF and electronic editions of The EMBO Journal will be copy edited, and you will be provided with page proofs prior to publication. Please note that supplementary information is not included in the proofs.

You will be contacted by Wiley Author Services to complete licensing and payment information. The required 'Page Charges Authorization Form' is available here: https://www.embopress.org/pb-assets/embo-site/tej_apc.pdf - please download and complete the form and return to embopressproduction@wiley.com

EMBO Press participates in many Publish and Read agreements that allow authors to publish Open Access with reduced/no publication charges. Check your eligibility: https://authorservices.wiley.com/author-resources/Journal-Authors/open-access/affiliation-policies-payments/index.html

Should you be planning a Press Release on your article, please get in contact with embojournal@wiley.com as early as possible, in order to coordinate publication and release dates.

If you have any questions, please do not hesitate to call or email the Editorial Office. Thank you for your contribution to The EMBO Journal.

Best wishes,

William

William Teale, PhD
Editor
The EMBO Journal
w.teale@embojournal.org

** Click here to be directed to your login page: https://emboj.msubmit.net

EMBO Press Author Checklist

Corresponding Author Name: Poul Nissen
Journal Submitted to: EMBO Journal
Manuscript Number: EMBOJ-2022-111122

USEFUL LINKS FOR COMPLETING THIS FORM
The EMBO Journal - Author Guidelines
EMBO Reports - Author Guidelines
Molecular Systems Biology - Author Guidelines
EMBO Molecular Medicine - Author Guidelines

Reporting Checklist for Life Science Articles (updated January
This checklist is adapted from Materials Design Analysis Reporting (MDAR) Checklist for Authors. MDAR establishes a minimum set of requirements in transparent reporting in the life sciences (see Statement of Task: 10.31222/osf.io/9sm4x). Please follow the journal's guidelines in preparing your
Please note that a copy of this checklist will be published alongside your article.

Abridged guidelines for figures
1. Data
The data shown in figures should satisfy the following conditions:
- the data were obtained and processed according to the field's best practice and are presented to reflect the results of the experiments in an accurate and unbiased manner.
- ideally, figure panels should include only measurements that are directly comparable to each other and obtained with the same assay.
- plots include clearly labeled error bars for independent experiments and sample sizes. Unless justified, error bars should not be shown for technical
- if n<5, the individual data points from each experiment should be plotted. Any statistical test employed should be justified.
- Source Data should be included to report the data underlying figures according to the guidelines set out in the authorship guidelines on Data

2. Captions
Each figure caption should contain the following information, for each panel where they are relevant:
- a specification of the experimental system investigated (eg cell line, species name).
- the assay(s) and method(s) used to carry out the reported observations and measurements.
- an explicit mention of the biological and chemical entity(ies) that are being measured.
- an explicit mention of the biological and chemical entity(ies) that are altered/varied/perturbed in a controlled manner.
- the exact sample size (n) for each experimental group/condition, given as a number, not a range;
- a description of the sample collection allowing the reader to understand whether the samples represent technical or biological replicates (including how many animals, litters, cultures, etc.).
- a statement of how many times the experiment shown was independently replicated in the laboratory.
- definitions of statistical methods and measures:
  - common tests, such as t-test (please specify whether paired vs. unpaired), simple χ^2 tests, Wilcoxon and Mann-Whitney tests, can be unambiguously identified by name only, but more complex techniques should be described in the methods section;
  - are tests one-sided or two-sided?
  - are there adjustments for multiple comparisons?
  - exact statistical test results, e.g., P values = x but not P values < x;
  - definition of 'center values' as median or average;
  - definition of error bars as s.d. or s.e.m.

Please complete ALL of the questions below.
Select "Not Applicable" only when the requested information is not relevant for your study.

Materials

Newly Created Materials	Information included in the manuscript?	In which section is the information available? (Reagents and Tools Table, Materials and Methods, Figures, Data Availability Section)
New materials and reagents need to be available; do any restrictions apply?	Not Applicable	

Antibodies	Information included in the manuscript?	In which section is the information available? (Reagents and Tools Table, Materials and Methods, Figures, Data Availability Section)
For **antibodies** provide the following information:<br>- Commercial antibodies: RRID (if possible) or supplier name, catalogue number and or/clone number<br>- Non-commercial: RRID or citation	Yes	Materials and methods

DNA and RNA sequences	Information included in the manuscript?	In which section is the information available? (Reagents and Tools Table, Materials and Methods, Figures, Data Availability Section)
**Short novel DNA or RNA including primers, probes**: provide the sequences.	Yes	Resource availability

Cell materials	Information included in the manuscript?	In which section is the information available? (Reagents and Tools Table, Materials and Methods, Figures, Data Availability Section)
**Cell lines:** Provide species information, strain. Provide accession number in repository **OR** supplier name, catalog number, clone number, and/**OR** RRID.	Not Applicable	
**Primary cultures:** Provide species, strain, sex of origin, genetic modification status.	Yes	Materials and methids
Report if the cell lines were recently **authenticated** (e.g., by STR profiling) and tested for mycoplasma contamination.	Not Applicable	

Experimental animals	Information included in the manuscript?	In which section is the information available? (Reagents and Tools Table, Materials and Methods, Figures, Data Availability Section)
**Laboratory animals or Model organisms:** Provide species, strain, sex, age, genetic modification status. Provide accession number in repository **OR** supplier name, catalog number, clone number, **OR** RRID.	Not Applicable	
**Animal observed in or captured from the field:** Provide species, sex, and age where possible**.**	Not Applicable	
Please detail **housing and husbandry conditions**.	Not Applicable	

Plants and microbes	Information included in the manuscript?	In which section is the information available? (Reagents and Tools Table, Materials and Methods, Figures, Data Availability Section)
**Plants:** provide species and strain, ecotype and cultivar where relevant, unique accession number if available, and source (including location for collected wild specimens).	Not Applicable	
**Microbes:** provide species and strain, unique accession number if available, and source.	Yes	Materials and methods

Human research participants	Information included in the manuscript?	In which section is the information available? (Reagents and Tools Table, Materials and Methods, Figures, Data Availability Section)
If collected and within the bounds of privacy constraints report on age, sex and gender or ethnicity for all study participants.	Not Applicable	

Core facilities	Information included in the manuscript?	In which section is the information available? (Reagents and Tools Table, Materials and Methods, Figures, Data Availability Section)
If your work benefited from core facilities, was their service mentioned in the acknowledgments section?	Yes	Acknowledgements

Design

Study protocol	Information included in the manuscript?	In which section is the information available? (Reagents and Tools Table, Materials and Methods, Figures, Data Availability Section)
If study protocol has been **pre-registered, provide DOI in the manuscript**. For clinical trials, provide the trial registration number **OR** cite DOI.	Not Applicable	
Report the **clinical trial registration number** (at ClinicalTrials.gov or equivalent), where applicable.	Not Applicable	

Laboratory protocol	Information included in the manuscript?	In which section is the information available? (Reagents and Tools Table, Materials and Methods, Figures, Data Availability Section)
Provide DOI OR other citation details if **external detailed step-by-step protocols** are available.	Not Applicable	

Experimental study design and statistics	Information included in the manuscript?	In which section is the information available? (Reagents and Tools Table, Materials and Methods, Figures, Data Availability Section)
Include a statement about **sample size** estimate even if no statistical methods were used.	Yes	Figures
Were any steps taken to minimize the effects of subjective bias when allocating animals/samples to treatment (e.g. **randomization procedure**)? If yes, have they been described?	Not Applicable	
Include a statement about **blinding** even if no blinding was done.	Not Applicable	
Describe **inclusion/exclusion criteria** if samples or animals were excluded from the analysis. Were the criteria pre-established?<br><br>If sample or data points were omitted from analysis, report if this was due to attrition or intentional exclusion and provide justification.	Not Applicable	
For every figure, are **statistical tests** justified as appropriate? Do the data meet the assumptions of the tests (e.g., normal distribution)? Describe any methods used to assess it. Is there an estimate of variation within each group of data? Is the variance similar between the groups that are being statistically compared?	Not Applicable	Figures

Sample definition and in-laboratory replication	Information included in the manuscript?	In which section is the information available? (Reagents and Tools Table, Materials and Methods, Figures, Data Availability Section)
In the figure legends: state number of times the experiment was **replicated** in laboratory.	Yes	Figure legends
In the figure legends: define whether data describe **technical or biological replicates**.	Yes	Figure legends

Ethics

Ethics	Information included in the manuscript?	In which section is the information available? (Reagents and Tools Table, Materials and Methods, Figures, Data Availability Section)
Studies involving **human participants**: State details of **authority granting ethics approval** (IRB or equivalent committee(s), provide reference number for approval.	Not Applicable	
Studies involving **human participants**: Include a statement confirming that **informed consent** was obtained from all subjects and that the experiments conformed to the principles set out in the WMA Declaration of Helsinki and the Department of Health and Human Services Belmont Report.	Not Applicable	
Studies involving **human participants:** For publication of **patient photos**, include a statement confirming that consent to publish was obtained.	Not Applicable	
Studies involving experimental **animals**: State details of **authority granting ethics approval** (IRB or equivalent committee(s), provide reference number for approval. Include a statement of compliance with ethical regulations.	Not Applicable	
Studies involving **specimen and field samples:** State if relevant **permits** obtained, provide details of authority approving study; if none were required, explain why.	Not Applicable	

Dual Use Research of Concern (DURC)	Information included in the manuscript?	In which section is the information available? (Reagents and Tools Table, Materials and Methods, Figures, Data Availability Section)
Could your study fall under dual use research restrictions? Please check biosecurity documents and list of **select agents and toxins** (CDC): https://www.selectagents.gov/sat/list.htm	Not Applicable	
If you used a select agent, is the security level of the lab appropriate and reported in the manuscript?	Not Applicable	
If a study is subject to dual use research of concern regulations, is the name of the **authority granting approval and reference number** for the regulatory approval provided in the manuscript?	Not Applicable	

Reporting

The MDAR framework recommends adoption of discipline-specific guidelines, established and endorsed through community initiatives. Journals have their own policy about requiring specific guidelines and recommendations to complement MDAR.

Adherence to community standards	Information included in the manuscript?	In which section is the information available? (Reagents and Tools Table, Materials and Methods, Figures, Data Availability Section)
State if relevant guidelines or checklists (e.g., **ICMJE, MIBBI, ARRIVE, PRISMA**) have been followed or provided.	Not Applicable	
For **tumor marker prognostic studies,** we recommend that you follow the **REMARK** reporting guidelines (see link list at top right). See author guidelines, under 'Reporting Guidelines'. Please confirm you have followed these guidelines.	Not Applicable	
For **phase II and III randomized controlled trials**, please refer to the **CONSORT** flow diagram (see link list at top right) and submit the CONSORT checklist (see link list at top right) with your submission. See author guidelines, under 'Reporting Guidelines'. Please confirm you have submitted this list.	Not Applicable	

Data Availability

Data availability	Information included in the manuscript?	In which section is the information available? (Reagents and Tools Table, Materials and Methods, Figures, Data Availability Section)
Have **primary datasets** been deposited according to the journal's guidelines (see 'Data Deposition' section) and the respective accession numbers provided in the Data Availability Section?	Not Applicable	
Were **human clinical and genomic datasets** deposited in a public access-controlled repository in accordance to ethical obligations to the patients and to the applicable consent agreement?	Not Applicable	
Are **computational models** that are central and integral to a study available without restrictions in a machine-readable form? Were the relevant accession numbers or links provided?	Not Applicable	
If publicly available data were reused, provide the respective **data citations in the reference list**.	Yes	Materials and methods
